# 3-[*N,N*-Bis(sulfonyl)amino]isoxazolines with Spiro-Annulated or 1,2-Annulated Cyclooctane Rings Inhibit Reproduction of Tick-Borne Encephalitis, Yellow Fever, and West Nile Viruses

**DOI:** 10.3390/ijms241310758

**Published:** 2023-06-28

**Authors:** Kseniya N. Sedenkova, Artem S. Sazonov, Dmitry A. Vasilenko, Kristian S. Andriasov, Marina G. Eremenko, Yuri K. Grishin, Evgeny V. Khvatov, Alexander S. Goryashchenko, Victoria I. Uvarova, Dmitry I. Osolodkin, Aydar A. Ishmukhametov, Elena B. Averina

**Affiliations:** 1 Department of Chemistry, Lomonosov Moscow State University, Moscow 119991, Russia; sedenkova@med.chem.msu.ru (K.N.S.); artem.sazonov@chemistry.msu.ru (A.S.S.); vda-ga@yandex.ru (D.A.V.); akristian@mail.ru (K.S.A.); eremenkomg@gmail.com (M.G.E.); grishin@nmr.chem.msu.ru (Y.K.G.); 2 FSASI “Chumakov FSC R&D IBP RAS” (Institute of Poliomyelitis), Moscow 108819, Russia; khvatov_ev@chumakovs.su (E.V.K.); goryaschenko_as@chumakovs.su (A.S.G.); uvarova_vi@chumakovs.su (V.I.U.); osolodkin_di@chumakovs.su (D.I.O.); ishmukhametov@chumakovs.su (A.A.I.); 3 Institute of Translational Medicine and Biotechnology, Sechenov Moscow State Medical University, Moscow 119991, Russia

**Keywords:** tick-borne encephalitis virus, yellow fever virus, West Nile virus, antivirals, isoxazolines, spiro-compounds, 1,2-annulated compounds, sulfonylation

## Abstract

Spirocyclic compounds containing heterocyclic moieties represent promising 3D scaffolds for modern drug design. In the search for novel anti-flaviviral agents, we have obtained a series of 3-[*N,N*-bis(sulfonyl)amino]isoxazolines containing spiro-annulated cyclooctane rings and assessed their antiviral activity against tick-borne encephalitis (TBEV), yellow fever (YFV), and West Nile (WNV) viruses. The structural analogs of spirocyclic compounds with a single sulfonyl group or 1,2-annulated cyclooctane ring were also investigated. Almost all the studied 3-[*N,N*-bis(sulfonyl)amino]isoxazolines revealed antiviral activity against TBEV and WNV. The most active against TBEV was spiro-isoxazoline derivative containing *p*-nitrophenyl groups in the sulfonyl part (EC_50_ 2.0 ± 0.5 μM), while the highest potency against WNV was found for the compounds with lipophilic substituents in sulfonyl moiety, naphtyl being the most favorable one (EC_50_ 1.3 ± 0.5 μM). In summary, two novel scaffolds of anti-flaviviral agents based on *N,N*-bis(sulfonyl)amino]isoxazoline were proposed, and the compounds of this type demonstrated activity against TBEV and WNV.

## 1. Introduction

**Figure 1:**
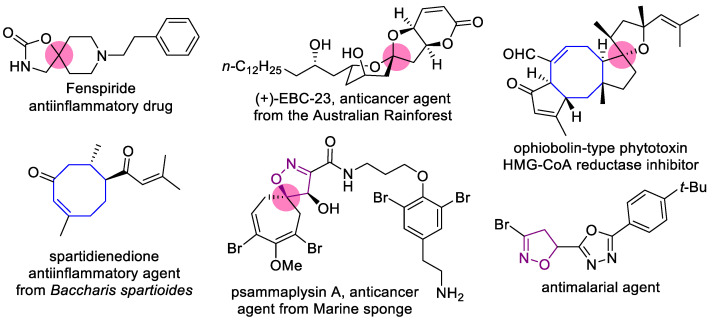
Examples of approved drugs, natural and bioactive compounds with spirocyclic motifs, medium rings, isoxazoline moieties, and their combinations.

In the past two decades, spirocyclic compounds appeared at the focus of research in the field of drug design and discovery as promising 3D scaffolds, enlarging the chemical space, allowing the construction of new types of lead compounds, and improving their potency, selectivity, and pharmacokinetic properties [1]. Spiroheterocyclic motifs are present in a number of approved drugs and natural compounds [1,2,3,4] (Figure 1). Such an interest in spirocyclic structures echoed in the field of synthetic organic chemistry, giving rise to a number of elegant preparative approaches to compounds of this type [5,6,7,8,9,10,11].

On the other hand, medium-sized rings (8–11 atoms) are rarely used for the construction of spirocyclic structures, reflecting the general tendency concerning medium rings. Although they possess appealing properties, such as a unique balance between rigidity and flexibility and diverse geometry, improve binding affinity to target enzymes, and are present in both bioactive and natural compounds (Figure 1) [12,13,14], application of medium rings in drug design is limited by the lack of synthetic approaches. Successful efforts to obtain the compounds containing medium rings, in most cases with heteroatoms, have been recently reported, illustrating the arising interest in such structures and the need for synthetic methods for them [15,16,17].

**Figure 2:**
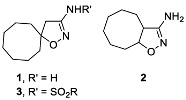
Previously obtained aminoisoxazoline derivatives **1–3 [26]**.

Isoxazoles and isoxazolines reveal a broad range of biological activities, including antitumor, antibacterial, antimalarial, antisclerotic, antidiabetic, and others (Figure 1), and a number of examples exist of how the insertion of an isoxazoline moiety increases the potency of the bioactive compounds [18,19,20,21,22,23]. The most general approach to isoxazoline derivatives is 1,3-dipolar cycloaddition, though other methods, taking advantage of metal-mediated and radical processes, are constantly gaining more significance [24,25]. Recently we have elaborated the preparative approach to spiro- and 1,2-annulated 3-aminoisoxazoles **1**,**2**, containing an 8-membered ring, and demonstrated their potent antiviral activity against influenza A virus (Figure 2) [26]. A series of 3-(*N-*sulfonylamino)isoxazolines **3** was also obtained and found to possess low-to-no cytotoxicity, making them attractive for the search of other applications in lead discovery [26].

**Figure 3:**
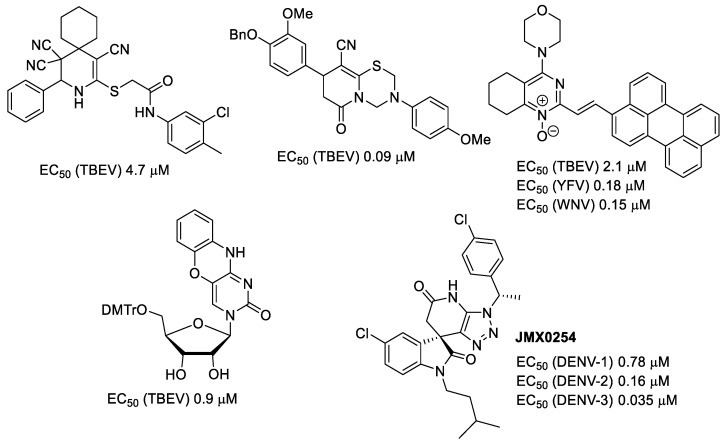
Examples of compounds with antiviral activity against TBEV, YFV, WNV, and DENV [31,32,33,35,39].

Flaviviruses are arthropod-borne viruses that are mainly transmitted between birds and wild animals by the bite of infected blood-sucking arthropods, such as mosquitoes or ticks. Unfortunately, humans could be bitten and infected too, resulting in fevers and encephalitides of different severity, from mild to even lethal, therefore representing a constant epidemic threat [27]. Currently, there are no specific medications for the treatment of flavivirus-borne diseases, so patient care is usually symptomatic, and the main way of preventing infection is vaccination [28]. Nevertheless, vaccines do not provide complete protectivity [29], and there is still no human vaccine against West Nile virus [30]. Thus, the development of specific anti-flaviviral drugs is still very important. Previously we have found several structural types of compounds showing antiviral activity against tick-borne encephalitis (TBEV), yellow fever (YFV), and West Nile (WNV) viruses [28,31,32,33,34,35,36,37,38]. As can be seen in examples (Figure 3), they possess quite a diverse structure.

**Figure 4:**
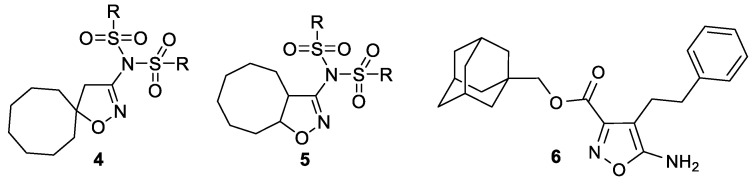
Structures of 3-[*N,N*-bis(sulfonyl)amino]isoxazolines **4**,**5**, investigated in the present work, and previously studied isoxazole derivative **6** that was used as a positive control **[41]**.

A heterocyclic core, spiro-annulated or 1,2-annulated to carbocycle and furnished with lipophilic substituents, may be distinguished as a peculiar pattern. For example, spiropyrazolopyridone derivatives were identified as dengue virus type 2 (DENV-2) inhibitors [40], while substituted 4,6-dihydrospiro[[1,2,3]triazolo[4,5-*b*]pyridine-7,3′-indoline]-2′,5(3*H*)-dione analogs such as JMX0254 (Figure 3) effectively inhibit NS4B protein activity of dengue virus type 1 (DENV-1) [39]. That inspired us to synthesize a series of novel 3-[*N,N*-bis(sulfonyl)amino]isoxazolines **4**,**5**, containing spiro-annulated or 1,2-annulated cyclooctane rings (Figure 4), and probe them as novel anti-flaviviral agents along with the previously obtained series of 3-(*N-*sulfonylamino)isoxazolines **3**.

## 2. Results and Discussion

### 2.1. Chemistry

Target 3-[*N,N*-bis(sulfonyl)amino]isoxazolines were obtained via sulfonylation of 3-aminoisoxazolines **1**,**2** or, in some cases, 3-(sulfonylamino)isoxazolines **3** upon treatment with corresponding sulfonyl chlorides (Table 1). Depending on the starting amine and sulfonyl chloride, the chosen method, reaction time, and reagent ratio were varied in order to improve the preparative yield (see Section 3.2).

The interaction of amines **1** and **2** with mesyl chloride and benzyl sulfonyl chloride in the presence of *N*,*N*-diisopropylethylamine (DIPEA) (Method A) gave hardly separable mixtures of the products of one- and two-fold sulfonylation, even when an excess of sulfonyl chloride was used. That resulted in lowering the yields of compounds **4a**,**b**, and **5b**, and despite all the efforts, we were not able to obtain compound **5a** in analytically pure form.

Spirocyclic isoxazoline **1** interacted with aryl sulfonyl chlorides in the presence of DIPEA (Method A), affording the products of two-fold sulfonylation, generally in moderate yields. Nevertheless, bis(sulfonyl)amines **4e**,**h** could not be obtained in these conditions. Compound **4e** was successfully obtained via sulfonylation of 3-(sulfonylamino)isoxazoline **3e** using the mixture of triethylamine (TEA) and 4-dimethylaminopyridine (DMAP) as the bases (Method C); this method was also probed for the synthesis of bis(sulfonyl)amine **4f** more the doubling the yield of the target compound. 2-Nitrobenzenesulfonyl chloride did not give products of two-fold sulfonylation in the presence of *N*-bases (DIPEA, TEA, DMAP). We managed to obtain compound **4h**, containing *o*-nitrophenyl substituents, only using pyridine as a solvent (Method B).

The reactions of isoxazoline **2** with both EWG- and EDG-substituted aryl sulfonyl chlorides in the presence of DIPEA (Method A) in most cases proceeded smoothly, giving 3-[*N,N*-bis(sulfonyl)amino]isoxazolines **5c–l** in the yields up to quantitative. The exceptions were sterically hindered *o*-substituted aryl sulfonyl and naphtyl sulfonyl chlorides—the reactions of compound **2** with the latter afforded target heterocycles **5e**,**h**,**j** in moderate yields.

Such a difference in reactivity between spirocyclic and bicyclic amines **1**,**2** could be explained according to their geometry. The spiro-annulated carbocycle in compound **1** hinders both sides of the isoxazoline ring, while 1,2-annulated cyclooctane leaves one side of the ring unhindered, thus not preventing the amino group from serving as a nucleophile.

### 2.2. Antiviral Activity Assessment

For two series of 3-[*N,N*-bis(sulfonyl)amino]isoxazolines **4a–l** and **5b–l**, antiviral activity was investigated against TBEV on porcine embryo kidney (PEK) cells using plaque reduction assay based on the estimation of the plaque-forming efficiency of a virus in the presence of different concentrations of a compound. Acute and chronic cytotoxicity of these compounds was studied using resazurin cell viability assay—viable cells turn resazurin into fluorescent resorufin (Table 2; see also Supplementary Materials). In order to compare the bioactivity of mono- and bissulfonylated derivatives, the same tests were performed for the series of previously obtained [26] 3-(*N*-sulfonylamino)isoxazolines **3a–h**,**j–l**. Previously studied isoxazole derivative **6** (Figure 4) that showed anti-TBEV activity [41] was used as a positive control. Despite it not being structurally similar to the compounds studied in this work, it allowed us to confirm the validity of the obtained EC_50_ values.

Most of bis(sulfonyl)amines **4** and **5** and several sulfonylamines **3** revealed anti-TBEV activity in micromolar concentrations. The most favorable substituents in sulfonyl moiety were found to be trimethylphenyl (**3–5e**), 2- or 4-nitrophenyl (**3–5h**, **4–5i**) and naphtyl (**3–5j**)—all the types of isoxazolines containing these groups demonstrated antiviral activity.

Comparing spiro-annulated sulfonyl- and bis(sulfonyl)amines **3c–h**,**j–l** and **4c–h**,**j–l**, containing aromatic substituents, one can observe that bis(sulfonyl) derivatives **4** systematically show higher potency than their mono-substituted analogs **3** (**3e**,**h**,**j** vs. **4e**,**h**,**j**) or demonstrate antiviral activity in the cases where compounds **3** are inactive (**3c**,**d**,**f**,**l** vs. **4c**,**d**,**f**,**l**). On the contrary, mono-benzyl substituted sulfonylamine **3b** was more potent than its bis-substituted congener **4b**.

Compounds **4b–l** and **5b–l**, containing spiro-annulated or 1,2-annulated cyclooctane moieties, did not show a clear relation between antiviral activity and the configuration of the two-ring system. Spiro-isoxazolines **4c**,**d**,**f**,**i**,**l** were more potent than 1,2-annulated analogs, while for compounds **4b**,**g**,**h**,**j**,**k**, the relation is opposite. Nevertheless, the most potent compounds of all the investigated species, namely **4c** and **4i**, belong to the spiro-isoxazoline series.

For all the compounds **3–5**, we have also determined the antiviral activity against WNV on African green monkey kidney (Vero) cells using the plaque reduction assay. The acute and chronic cytotoxicity of these compounds was studied using the resazurin cell viability assay (Table 3). Favipiravir was used as a positive control in this screening [42].

Almost all the tested compounds, except **5l**, inhibited WNV reproduction. In sulfonylamine series **3**, the most active were benzyl- and thiophenyl-substituted derivatives **3b** and **3k**. All the bis(sulfonyl)amines **4** were active in micromolar concentrations. Bis(sulfonyl)amines **4c–l** bearing aromatic or heteroaromatic substituents demonstrated higher potency than methyl or benzyl substituted compounds **4a**,**b**. The highest potency was found for compounds **4c–e**,**j** with lipophilic substituents in sulfonyl moiety, naphtyl being the most favorable one. Isoxazolines **5** containing the 1,2-annulated cyclooctane moiety generally demonstrated lesser anti-WNV activity than their spiro-annulated analogs **4**, except benzyl derivative **5b**, that was more active than its analog **4b**, and almost equally active compounds **4e** and **5e**. It is also interesting that the addition of the second pyridyl group to the sulfonylamine **3l**, resulting in the compound **4l**, did not affect the EC_50_ value, while a change in the position of cyclooctane moiety from spiro-annulated to 1,2-annulated one (compounds **4l** and **5l**, respectively) resulted in the complete loss of anti-WNV activity.

Finally, all compounds **3–5** have been tested for anti-YFV activity. Spiro-isoxazoline **3b**, bearing benzylsulfonyl moiety, was found to be active with EC_50_ 24 ± 11 μM, while other isoxazolines demonstrated no activity in concentrations up to 50 μM. Compound **6** was used as a positive control.

It should be noted that all the tested compounds demonstrated no acute cytotoxicity against PEK cells (see Supplementary Materials), and only compound **5i** was slightly toxic during 7 days of incubation. In the case of Vero cells, compounds **3j**, **4i**, and **5k** showed both acute and chronic cytotoxicity, while **4h** was only acute toxic (Table 3; see also Supplementary Materials). Thus, the compounds studied here represent a substantially novel class of non-toxic antivirals, deserving further detailed studies of the mechanism of action and antiviral efficiency in vivo.

## 3. Materials and Methods

### 3.1. General Remarks 

^1^H and ^13^C NMR spectra were recorded on a 400 MHz spectrometer Agilent 400-MR (400.0 and 100.6 MHz for ^1^H and ^13^C, respectively; Agilent Technologies, Santa Clara, CA, USA) at r.t. in CDCl_3_; chemical shifts *δ* were measured with reference to the solvent (CDCl_3_, *δ*_H_ = 7.26 ppm, *δ*_C_ = 77.16 ppm). When necessary, assignments of signals in NMR spectra were made using 2D techniques. Accurate mass measurements (HRMS) were obtained on Bruker micrOTOF II (Bruker Daltonik GmbH, Bremen, Gemany) with electrospray ionization (ESI). Analytical thin-layer chromatography was carried out with silica gel plates supported on aluminum (ALUGRAM^®^ Xtra SIL G/UV_254_, Macherey-Nagel, Duren, Germany); the detection was performed by a UV lamp (254 nm). Column chromatography was performed on silica gel (Silica 60, 0.015–0.04 mm, Macherey-Nagel, Duren, Germany). 3-Aminoisoxazolines **1**,**2** and compounds **3a–l** were obtained via described methods [26]. All other starting materials were commercially available. All reagents except commercial products of satisfactory quality were purified according to literature procedures prior to use.

Stock solutions of the compounds with a concentration of 5 mM were prepared in DMSO (Amresco, Cleveland, OH, USA).

### 3.2. Synthesis of Compounds **4**,**5** (General Methods)

**Method A.** To the solution of 3-aminoisoxazoline **1** or **2** (0.18 mmol) in dry DCM (2 mL), DIPEA (4 equiv, 0.72 mmol, 93 mg, 125 μL) was added under argon. The reaction mixture was cooled down to 0 °C, and sulfonyl chloride (0.9–5.5 equiv, 0.16–1.0 mmol) was added. Then the mixture was allowed to warm up to r.t., stirred for 3 h–8 days, and quenched with water (6 mL). The organic layer was separated, and the water layer was extracted with DCM (3 × 6 mL). Combined organic layers were washed with saturated aqueous NaHCO_3_ (9 mL) and brine (9 mL) and dried over MgSO_4_. The solvent was evaporated under reduced pressure. The product was isolated via preparative column chromatography (SiO_2_).

**Method B.** To the solution of 3-aminoisoxazoline **1** (0.28 mmol, 50 mg) in dry pyridine (1 mL), DIPEA (7.9 equiv, 2.2 mmol, 284 mg, 0.38 mL) was added under argon. The reaction mixture was cooled down to 0 °C, and 2-nitrobenzenesulfonyl chloride (5.9 equiv, 1.65 mmol, 365 mg) in dry pyridine (0.5 mL) was added. Then the mixture was allowed to warm up to r.t., stirred for 3 days, and quenched with 1M HCl (6 mL). The organic layer was separated, and the water layer was extracted with DCM (3 × 6 mL). Combined organic layers were washed with saturated aqueous NaHCO_3_ (9 mL) and brine (9 mL) and dried over MgSO_4_. The solvent was evaporated under reduced pressure. The product was isolated via preparative column chromatography (SiO_2_).

**Method C.** To the solution of 3-(sulfonylamino)isoxazoline **3e** or **3f** (0.1 mmol) in dry DCM (1.2 mL), TEA (3 equiv, 0.3 mmol, 0.04 mL, 30 mg) and DMAP (0.2 equiv, 0.02 mmol, 2.5 mg) were added under argon. The reaction mixture was cooled down to 0 °C, and sulfonyl chloride (1.1–2.4 equiv, 0.11–0.24 mmol) was added. Then the mixture was allowed to warm up to the r.t., stirred for 1–2 days, and quenched with water (5 mL). The organic layer was separated, and the water layer was extracted with DCM (3 × 5 mL). Combined organic layers were washed with brine (9 mL) and dried over MgSO_4_. The solvent was evaporated under reduced pressure. The product was isolated via preparative column chromatography (SiO_2_).

#### 3.2.1. *N*-(Methylsulfonyl)-*N*-(1-oxa-2-azaspiro[4.7]dodec-2-en-3-yl)methanesulfonamide (**4a**)

Yield 14 mg (23%), method A; reaction time 2 days; 1 mmol of sulfonyl chloride was used. White crystals, m.p. 139–141 °C. R*_f_* = 0.20 (petroleum ether:EtOAc = 8:1).

^1^H NMR (*δ*, ppm): 1.40–1.85 (m, 12H, 7CH_2_); 2.09–2.17 (m, 2H, 2CH_2_); 2.81 (s, 2H, CH_2_, Isox); 3.46 (s, 6H, 2CH_3_).

^13^C NMR (*δ*, ppm): 22.0 (2CH_2_), 24.4 (CH_2_), 28.0 (2CH_2_), 35.0 (2CH_2_), 43.8 (CH_2_, Isox), 45.6 (2CH_3_), 95.3 (C_spiro_), 149.4 (C, Isox).

HRMS (ESI^+^, *m*/*z*): calculated for C_12_H_22_N_2_O_5_S_2_ [M+H]^+^, 339.1043; found, 339.1042.

#### 3.2.2. *N*-(Benzylsulfonyl)-1-phenyl-*N*-(1-oxa-2-azaspiro[4.7]dodec-2-en-3-yl)methanesulfonamide (**4b**)

Yield 15 mg (19%), method A; reaction time 3 days; 0.16 mmol of sulfonyl chloride was used. White crystals, m.p. 176–177 °C. R*_f_* = 0.28 (petroleum ether:DCM = 1:2).

^1^H NMR (*δ*, ppm): 1.19–1.30 (m, 2H, 2CH_2_); 1.30–1.44 (m, 2H, 2CH_2_); 1.44–1.66 (m, 8H, 7CH_2_); 1.49 (s, 2H, CH_2_, Isox); 1.85–1.96 (m, 2H, 2CH_2_); 4.87 (s, 4H, 2CH_2_, Bn); 7.36–7.46 (m, 6H, 6CH, Ph); 7.47–7.57 (m, 4H, 4CH, Ph).

^13^C NMR (*δ*, ppm): 21.9 (2CH_2_), 24.3 (CH_2_), 27.9 (2CH_2_), 34.7 (2CH_2_), 43.5 (CH_2_, Isox), 61.8 (2CH_2_, Bn), 94.8 (C_spiro_), 126.8 (C, Isox), 129.2 (4CH, Ph), 129.7 (2CH, Ph), 131.8 (4CH, Ph), 150.4 (2C, Ph).

HRMS (ESI^+^, *m*/*z*): calculated for C_24_H_30_N_2_O_5_S_2_ [M+H]^+^, 491.1669; found, 491.1667.

#### 3.2.3. *N*-(Phenylsulfonyl)-*N*-(1-oxa-2-azaspiro[4.7]dodec-2-en-3-yl)benzenesulfonamide (**4c**)

Yield 59 mg (71%), method A; reaction time 2 days; 1 mmol of sulfonyl chloride was used. White crystals, m.p. 139–141 °C. R*_f_* = 0.21 (petroleum ether:EtOAc = 3:1).

^1^H NMR (*δ*, ppm): 1.34–1.65 (m, 5H, 5CH_2_); 1.66–1.81 (m, 3H, 3CH_2_); 1.84–2.00 (m, 4H, 4CH_2_); 2.44–2.58 (m, 2H, 2CH_2_); 3.03 (s, 2H, CH_2_, Isox); 7.30–7.41 (m, 4H, 4CH, Ph); 7.51–7.60 (m, 2H, 2CH, Ph); 7.69–7.77 (m, 4H, 4CH, Ph).

^13^C NMR (*δ*, ppm): 22.7 (2CH_2_), 25.0 (CH_2_), 28.0 (2CH_2_), 28.4 (CH_2_, Isox), 33.2 (2CH_2_), 93.1 (C_spiro_), 118.0 (C, Isox), 128.9 (4CH, Ph), 129.6 (4CH, Ph), 134.5 (2CH, Ph), 135.3 (2C(SO_2_)).

HRMS (ESI^+^, *m*/*z*): calculated for C_22_H_26_N_2_O_5_S_2_ [M+NH_4_]^+^, 480.1621; found, 480.1622.

#### 3.2.4. 4-Methyl-*N*-(1-oxa-2-azaspiro[4.7]dodec-2-en-3-yl)-*N*-tosylbenzenesulfonamide (**4d**)

Yield 49 mg (56%), method A; reaction time 2 days; 1 mmol of sulfonyl chloride was used. White crystals, m.p. 151–153 °C. R*_f_* = 0.41 (petroleum ether:DCM = 1:3).

^1^H NMR (*δ*, ppm): 1.34–1.65 (m, 5H, 5CH_2_); 1.66–1.81 (m, 3H, 3CH_2_); 1.84–2.00 (m, 4H, 4CH_2_); 2.40 (s, 6H, 2CH_3_); 2.43–2.56 (m, 2H, 2CH_2_); 3.02 (s, 2H, CH_2_, Isox); 7.08–7.19 (m, 4H, 4CH, Ar); 7.57–7.67 (m, 4H, 4CH, Ar).

^13^C NMR (*δ*, ppm): 21.9 (2CH_3_), 22.7 (2CH_2_), 25.0 (CH_2_), 28.0 (2CH_2_), 28.3 (CH_2_, Isox), 33.2 (2CH_2_), 92.8 (C_spiro_), 118.1 (C, Isox), 129.4 (4CH, Ar), 129.7 (4CH, Ar), 132.4 (2C(SO_2_)), 145.8 (2C, Ar).

HRMS (ESI^+^, *m*/*z*): calculated for C_24_H_30_N_2_O_5_S_2_ [M+NH_4_]^+^, 508.1934; found, 508.1939.

#### 3.2.5. *N*-(Mesitylsulfonyl)-2,4,6-trimethyl-*N*-(1-oxa-2-azaspiro[4.7]dodec-2-en-3-yl)benzenesulfonamide (**4e**)

Yield 33 mg (60%), method C; 0.11 mmol of sulfonyl chloride was used. White crystals, m.p. 180–182 °C. R*_f_* = 0.21 (petroleum ether:EtOAc = 7:1).

^1^H NMR (*δ*, ppm): 1.20–1.73 (m, 14H, 7CH_2_); 2.33 (s, 6H, 2CH_3_); 2.61 (s, 2H, CH_2_, Isox); 2.74 (s, 12H, 4CH_3_); 7.01 (s, 4H, 4CH, Ar).

^13^C NMR (*δ*, ppm): 21.4 (2CH_3_), 22.2 (2CH_2_), 23.6 (4CH_3_), 24.1 (CH_2_), 27.8 (2CH_2_, cy-Oct + CH_2_, Isox), 33.3 (2CH_2_), 91.4 (C_spiro_), 117.3 (C, Isox), 132.3 (4CH, Ar + 2C(SO_2_)), 142.8 (4C, Ar), 145.2 (2C, Ar).

HRMS (ESI^+^, *m*/*z*): calculated for C_28_H_38_N_2_O_5_S_2_ [M+NH_4_]^+^, 546.2560; found, 546.2553.

#### 3.2.6. *N*-((2,3-Dihydrobenzo[*b*][1,4]dioxin-6-yl)sulfonyl)-*N*-(1-oxa-2-azaspiro[4.7]dodec-2-en-3-yl)-2,3-dihydrobenzo[*b*][1,4]dioxine-6-sulfonamide (**4f**)

Yield 38 mg (36%), method A; reaction time 2 days, 1 mmol of sulfonyl chloride was used; 35 mg (61%), method C; 0.24 mmol of sulfonyl chloride was used. White crystals, m.p. 191–193 °C. R*_f_* = 0.32 (petroleum ether:EtOAc = 1:1).

^1^H NMR (*δ*, ppm; *J*, Hz): 1.34–1.65 (m, 5H, 5CH_2_); 1.66–1.81 (m, 3H, 3CH_2_); 1.84–2.00 (m, 4H, 4CH_2_); 2.46–2.58 (m, 2H, 2CH_2_); 3.03 (s, 2H, CH_2_, Isox); 4.20–4.36 (m, 8H, 4CH_2_O); 6.77 (br.d, ^3^*J* 8.6, 1H, CH, Ar); 7.20 (br.d, ^4^*J* 2.3, 1H, CH, Ar); 7.12–7.20 (m, 2H, 2CH, Ar); 7.23 (dd, ^3^*J* 8.6, ^4^*J* 2.3, 1H, CH, Ar).

^13^C NMR (*δ*, ppm): 22.2 (2CH_2_), 25.0 (CH_2_), 28.0 (2CH_2_), 28.4 (CH_2_, Isox), 33.1 (2CH_2_), 64.1 (2CH_2_O), 64.8 (2CH_2_O), 92.9 (C_spiro_), 117.3 (2CH, Ar), 118.2 (C, Isox), 119.2 (2CH, Ar), 123.5 (2CH, Ar), 127.1 (2C, Ar), 143.1 (2C, Ar), 149.0 (2C, Ar).

HRMS (ESI^+^, *m*/*z*): calculated for C_26_H_30_N_2_O_9_S_2_ [M+Na]^+^, 601.1285; found, 601.1280.

#### 3.2.7. *N*-((2,4-Difluorophenyl)sulfonyl)-2,4-difluoro-*N*-(1-oxa-2-azaspiro[4.7]dodec-2-en-3-yl)benzenesulfonamide (**4g**)

Yield 45 mg (47%), method A; reaction time 4 days, 0.54 mmol of sulfonyl chloride was used. White crystals, m.p. 61–62 °C. R*_f_* = 0.56 (DCM).

^1^H NMR (*δ*, ppm; *J*, Hz): 1.32–1.53 (m, 3H, 3CH_2_); 1.54–1.66 (m, 2H, 2CH_2_); 1.68–1.78 (m, 3H, 3CH_2_); 1.78–1.90 (m, 2H, 2CH_2_); 1.95 (ddd, ^2^*J*_HH_ 15.2, ^3^*J*_HH_ 8.1, ^3^*J*_HH_ 2.2, 2H, 2CH_2_); 2.45 (ddd, ^2^*J*_HH_ 15.2, ^3^*J*_HH_ 10.6, ^3^*J*_HH_ 2.1, 2H, 2CH_2_); 3.00 (s, 2H, CH_2_, Isox); 6.98 (ddd, ^3^*J*_HF_ 10.2, ^3^*J*_HF_ 8.5, ^4^*J*_HH_ 4.0, 2H, 2CH, Ar); 7.08 (dddd, ^3^*J*_HF_ 7.4, ^5^*J*_HF_ 1.1, ^3^*J*_HH_ 9.0, ^4^*J*_HH_ 2.5, 2H, 2CH, Ar); 8.02 (ddd, ^4^*J*_HF_ 7.8, ^4^*J*_HF_ 6.0, ^3^*J*_HH_ 9.0, 2H, 2CH, Ar).

^13^C NMR (*δ*, ppm; *J*, Hz): 22.7 (2CH_2_); 25.1 (CH_2_); 28.0 (2CH_2_); 28.2 (CH_2_, Isox); 33.1 (2CH_2_); 93.5 (C_spiro_); 106.2 (dd, ^2^*J*_CF_ 26, ^2^*J*_CF_ 26, 2CH, Ar); 112.4 (dd, ^2^*J*_CF_ 22, ^4^*J*_CF_ 4, 2CH, Ar); 117.7 (C, Isox); 120.0 (dd, ^2^*J*_CF_ 13, ^2^*J*_CF_ 4, 2C(SO_2_)); 135.5 (d, ^3^*J*_CF_ 11, 2CH, Ar); 161.2 (dd, ^1^*J*_CF_ 265, ^3^*J*_CF_ 14, 2CF, Ar); 167.7 (dd, ^1^*J*_CF_ 262, ^3^*J*_CF_ 12, 2CF, Ar).

^19^F NMR (*δ*, ppm; *J*, Hz): −98.03 (dddd, ^4^*J*_FF_ 15.3, ^3^*J*_HF_ 10.2, ^4^*J*_HF_ 7.8, ^5^*J*_HF_ 1.1, 2F, CF, Ar); −95.27 (dddd, ^4^*J*_FF_ 15.3, ^3^*J*_HF_ 8.5, ^3^*J*_HF_ 7.4, ^4^*J*_HF_ 6.0, 2F, CF, Ar).

HRMS (ESI^+^, *m*/*z*): calculated for C_22_H_22_F_4_N_2_O_5_S_2_ [M+Na]^+^, 557.0798; found, 557.0795.

#### 3.2.8. 2-Nitro-*N*-((2-nitrophenyl)sulfonyl)-*N*-(1-oxa-2-azaspiro[4.7]dodec-2-en-3-yl)benzenesulfonamide (**4h**)

Yield 30 mg (20%), method B. Light-yellow crystals, m.p. 155–157 °C. R*_f_* = 0.23 (petroleum ether:EtOAc = 2:1).

^1^H NMR (*δ*, ppm): 1.33–1.95 (m, 12H, 7CH_2_); 2.33–2.42 (m, 2H, 2CH_2_); 2.95 (s, 2H, CH_2_, Isox); 7.61–7.66 (m, 2H, 2CH, Ar); 7.71–7.79 (m, 4H, 4CH, Ar); 8.31–8.37 (m, 2H, 2CH, Ar).

^13^C NMR (*δ*, ppm): 22.6 (2CH_2_), 25.1 (CH_2_), 27.9 (2CH_2_), 28.3 (CH_2_, Isox), 32.9 (2CH_2_), 94.6 (C_spiro_), 117.5 (C, Isox), 124.5 (2CH), 130.1 (2C(SO_2_)), 132.1 (2CH), 132.5 (2CH), 135.7 (2CH), 148.4 (2C(NO_2_)).

HRMS (ESI^+^, *m*/*z*): calculated for C_22_H_24_N_4_O_9_S_2_ [M+NH_4_]^+^, 570.1323; found, 570.1322.

#### 3.2.9. 4-Nitro-*N*-((4-nitrophenyl)sulfonyl)-*N*-(1-oxa-2-azaspiro[4.7]dodec-2-en-3-yl)benzenesulfonamide (**4i**)

Yield 19 mg (57%), method A; reaction time 3 h, 0.63 mmol of sulfonyl chloride was used. White crystals, m.p. 178–180 °C. R*_f_* = 0.33 (petroleum ether:EtOAc = 4:1).

^1^H NMR (*δ*, ppm): 1.33–1.46 (m, 3H, 3CH_2_); 1.51–1.64 (m, 2H, 2CH_2_); 1.68–1.90 (m, 7H, 7CH_2_); 2.36–2.49 (m, 2H, 2CH_2_); 2.97 (s, 2H, CH_2_, Isox); 8.21–8.29 (m, 4H, 4CH); 8.35–8.43 (m, 4H, 4CH).

^13^C NMR (*δ*, ppm): 22.7 (2CH_2_), 25.2 (CH_2_), 27.9 (2CH_2_), 28.1 (CH_2_, Isox), 33.3 (2CH_2_), 94.0 (C_spiro_), 117.7 (C, Isox), 124.2 (4CH), 131.6 (4CH), 141.2 (2C(SO_2_)), 151.5 (2C(NO_2_)).

HRMS (ESI^+^, *m*/*z*): calculated for C_22_H_24_N_4_O_9_S_2_ [M+Na]^+^, 575.0877; found, 575.0876.

#### 3.2.10. *N*-(Naphthalen-2-ylsulfonyl)-*N*-(1-oxa-2-azaspiro[4.7]dodec-2-en-3-yl)naphthalene-2-sulfonamide (**4j**)

Yield 73 mg (72%), method A; reaction time 8 days, 0.54 mmol of sulfonyl chloride was used. White crystals, m.p. 160–161 °C. R*_f_* = 0.39 (petroleum ether:DCM = 1:4).

^1^H NMR (*δ*, ppm; *J*, Hz): 1.36–1.88 (m, 8H, 5CH_2_); 1.92–2.15 (m, 4H, 4CH_2_); 2.61–2.75 (m, 2H, 2CH_2_); 3.14 (s, 2H, CH_2_, Isox); 7.36 (d, ^3^*J* 8.7, 2H, 2CH); 7.38–7.42 (m, 2H, 2CH); 7.45–7.50 (m, 2H, 2CH); 7.50–7.53 (m, 4H, 4CH); 7.55 (dd, ^3^*J* 8.7, ^4^*J* 1.9, 2H, 2CH); 8.09 (d, ^4^*J* 1.9, 2H, 2CH).

^13^C NMR (*δ*, ppm): 22.7 (2CH_2_), 25.0 (CH_2_), 28.1 (2CH_2_), 28.6 (CH_2_, Isox), 33.2 (2CH_2_), 93.6 (C_spiro_), 118.2 (C, Isox), 123.2 (2CH), 127.65 (2CH), 127.67 (2CH), 128.7 (2CH), 129.3 (2CH), 129.7 (2CH), 131.31 (2C), 131.38 (2C), 131.40 (2CH), 135.2 (2C).

HRMS (ESI^+^, *m*/*z*): calculated for C_30_H_30_N_2_O_5_S_2_ [M+Na]^+^, 585.1488; found, 585.1477.

#### 3.2.11. *N*-(1-Oxa-2-azaspiro[4.7]dodec-2-en-3-yl)-*N*-(thiophen-2-ylsulfonyl)thiophene-2-sulfonamide (**4k**)

Yield 27 mg (32%), method A; reaction time 2 days; 1 mmol of sulfonyl chloride was used. White crystals, m.p. 147–147 °C. R*_f_* = 0.2 (petroleum ether:DCM = 1:3)

^1^H NMR (*δ*, ppm; *J*, Hz): 1.34–2.03 (m, 12H, 7CH_2_); 2.48–2.61 (m, 2H, CH_2_); 3.01 (s, 2H, CH_2_, Isox); 6.95–6.99 (m, 2H, 2CH, Tioph); 7.57–7.60 (m, 2H, 2CH, Tioph); 7.65–7.68 (m, 2H, 2CH, Tioph).

^13^C NMR (*δ*, ppm): 22.8 (2CH_2_), 25.1 (CH_2_), 28.1 (2CH_2_), 28.5 (CH_2_, Isox), 33.2 (2CH_2_), 93.6 (C_spiro_), 117.8 (C, Isox), 127.2 (2CH, Tioph), 133.6 (2C(SO_2_)), 136.3 (2CH, Tioph), 137.2(2CH, Tioph).

HRMS (ESI^+^, *m*/*z*): calculated for C_18_H_22_N_2_O_5_S_4_ [M+Na]^+^, 497.0304; found, 497.0300.

#### 3.2.12. *N*-(Pyridin-3-ylsulfonyl)-*N*-(1-oxa-2-azaspiro[4.7]dodec-2-en-3-yl)pyridine-3-sulfonamide (**4l**)

Yield 77 mg (92%), method A; reaction time 4 days, 0.54 mmol of sulfonyl chloride was used. White crystals, m.p. 151–152 °C. R*_f_* = 0.41 (DCM:MeOH = 25:1).

^1^H NMR (*δ*, ppm; *J*, Hz): 1.34–1.52 (m, 3H, 3CH_2_); 1.54–1.66 (m, 2H, 2CH_2_); 1.69–1.97 (m, 7H, 7CH_2_); 2.43–2.55 (m, 2H, 2CH_2_); 3.00 (s, 2H, CH_2_, Isox); 7.45 (ddd, ^3^*J*_HH_ 8.2, ^3^*J*_HH_ 4.9, ^5^*J*_HH_ 0.8, 2H, 2CH, Py); 8.25 (ddd, ^3^*J*_HH_ 8.2, ^4^*J*_HH_ 2.4, ^4^*J*_HH_ 1.6, 2H, 2CH, Py); 8.86 (dd, ^3^*J*_HH_ 4.9, ^4^*J*_HH_ 1.6, 2H, 2CH, Py); 9.02 (dd, ^4^*J*_HH_ 2.4, ^5^*J*_HH_ 0.8, 2H, 2CH, Py).

^13^C NMR (*δ*, ppm): 22.7 (2CH_2_), 25.1 (CH_2_), 27.9 (2CH_2_), 28.2 (CH_2_, Isox), 33.2 (2CH_2_), 93.9 (C_spiro_), 117.8 (C, Isox), 123.8 (2CH, Py), 132.3 (2C(SO_2_)), 137.6 (2CH, Py), 150.1 (2CH, Py), 155.2 (2CH, Py).

HRMS (ESI^+^, *m*/*z*): calculated for C_20_H_24_N_4_O_5_S_2_ [M+H]^+^, 465.1261; found, 465.1256.

#### 3.2.13. *N*-(Benzylsulfonyl)-*N*-(3a,4,5,6,7,8,9,9a-octahydrocycloocta[*d*]isoxazol-3-yl)-1-phenylmethanesulfonamide (**5b**)

Yield 25 mg (29%), method A; reaction time 1 day, 0.54 mmol of sulfonyl chloride was used. White crystals, m.p. 198–199 °C. R*_f_* = 0.45 (petroleum etherl:EtOAc = 8:1).

^1^H NMR (*δ*, ppm; *J*, Hz): 0.30–0.48 (m, 1H, CH_2_); 0.92–1.03 (m, 2H, CH_2_); 1.10–1.62 (m, 7H, 4CH_2_); 1.82–1.95 (m, 2H, CH_2_); 2.43–2.55 (m, 1H, CH, cy-Oct); 4.53–4.64 (m, 1H, CH-O, cy-Oct); 4.89 (d, ^3^*J* 13.9, 2H, 2CH_2_, Bn); 5.09 (d, ^3^*J* 13.9, 2H, 2CH_2_, Bn); 7.37–7.47 (m, 6H, 6CH, Ph); 7.47–7.55 (m, 4H, 4CH, Ph).

^13^C NMR (*δ*, ppm): 22.7 (CH_2_), 25.1 (CH_2_), 25.2 (CH_2_), 25.6 (CH_2_), 26.1 (CH_2_), 29.8 (CH_2_), 49.5 (CH, cy-Oct), 61.7 (2CH_2_, Bn), 88.1 (CH-O, cy-Oct), 126.7 (C, Isox), 129.2 (4CH, Ph), 129.7 (2CH, Ph), 131.6 (4CH, Ph), 154.9 (2C, Ph).

HRMS (ESI^+^, *m*/*z*): calculated for C_23_H_28_N_2_O_5_S_2_ [M+H]^+^, 477.1512; found, 477.1502.

#### 3.2.14. *N*-(3a,4,5,6,7,8,9,9a-Octahydrocycloocta[*d*]isoxazol-3-yl)-*N*-(phenylsulfonyl)benzenesulfonamide (**5c**)

Yield 63 mg (78%), method A; reaction time 6 h, 0.45 mmol of sulfonyl chloride was used. White crystals, m.p. 107–108 °C. R*_f_* = 0.56 (DCM).

^1^H NMR (*δ*, ppm): 1.36–2.16 (m, 11H, 6CH_2_); 2.17–2.31 (m, 1H, CH_2_); 3.76–3.87 (m, 1H, CH, cy-Oct); 4.62–4.73 (m, 1H, CH-O, cy-Oct); 7.22–7.41 (m, 4H, 4CH, Ph); 7.47–7.61 (m, 2H, 2CH, Ph); 7.61–7.73 (m, 4H, 4CH, Ph).

^13^C NMR (*δ*, ppm): 23.4 (CH_2_), 24.2 (CH_2_), 25.2 (CH_2_), 27.4 (CH_2_), 27.9 (CH_2_), 29.7 (CH_2_), 34.6 (CH, cy-Oct), 89.9 (CH-O, cy-Oct), 120.4 (C, Isox), 128.8 (2CH, Ph), 129.0 (2CH, Ph), 129.4 (2CH, Ph), 129.6 (2CH, Ph), 134.1 (2C(SO_2_)), 134.6 (CH, Ph), 134.7 (CH, Ph).

HRMS (ESI^+^, *m*/*z*): calculated for C_21_H_24_N_2_O_5_S_2_ [M+H]^+^, 449.1199; found, 449.1198.

#### 3.2.15. 4-Methyl-*N*-(3a,4,5,6,7,8,9,9a-octahydrocycloocta[*d*]isoxazol-3-yl)-*N*-tosylbenzenesulfonamide (**5d**)

Yield 65 mg (76%), method A; reaction time 1 day, 0.54 mmol of sulfonyl chloride was used. White crystals, m.p. 113–114 °C. R*_f_* = 0.21 (petroleum ether:EtOAc = 2:1).

^1^H NMR (*δ*, ppm): 1.36–1.84 (m, 8H, 4CH_2_); 1.86–2.15 (m, 3H, 2CH_2_); 2.16–2.30 (m, 1H, CH_2_); 2.39 (s, 3H, CH_3_); 2.40 (s, 3H, CH_3_); 3.75–3.86 (m, 1H, CH, cy-Oct); 4.58–4.68 (m, 1H, CH-O, cy-Oct); 7.00–7.19 (m, 4H, 4CH, Ar); 7.52–7.60 (m, 4H, 4CH, Ar).

^13^C NMR (*δ*, ppm): 21.8 (2CH_3_), 23.4 (CH_2_), 24.2 (CH_2_), 25.2 (CH_2_), 27.5 (CH_2_), 28.0 (CH_2_), 29.7 (CH_2_), 34.6 (CH, cy-Oct), 89.6 (CH-O, cy-Oct), 120.4 (C, Isox), 129.3 (2CH, Ar), 129.5 (4CH, Ar), 129.8 (2CH, Ar), 131.3 (C(SO_2_)), 132.0 (C(SO_2_)), 145.9 (C, Ar), 146.1 (C, Ar).

HRMS (ESI^+^, *m*/*z*): calculated for C_23_H_28_N_2_O_5_S_2_ [M+H]^+^, 477.1512; found, 477.1505.

#### 3.2.16. *N*-(Mesitylsulfonyl)-2,4,6-trimethyl-*N*-(3a,4,5,6,7,8,9,9a-octahydrocycloocta[*d*]isoxazol-3-yl)benzenesulfonamide (**5e**)

Yield 33 mg (34%), method A; reaction time 3 days, 0.54 mmol of sulfonyl chloride was used. White crystals, m.p. 148–149 °C. R*_f_* = 0.47 (DCM).

^1^H NMR (*δ*, ppm): 0.97–1.17 (m, 2H, 2CH_2_); 1.20–1.67 (m, 8H, 6CH_2_); 1.82–1.95 (m, 2H, 2CH_2_); 2.33 (s, 6H, 2CH_3_); 2.74 (br.s, 12H, 4CH_3_); 3.13–3.20 (m, 1H, CH, cy-Oct); 3.68–3.78 (m, 1H, CH-O, cy-Oct); 7.03 (br.s, 4H, 4CH, Ar).

^13^C NMR (*δ*, ppm): 21.3 (2CH_3_), 22.9 (CH_2_), 23.4 (br.s, 4CH_3_ + CH_2_), 25.2 (CH_2_), 27.3 (CH_2_), 29.3 (CH_2_), 29.4 (CH_2_), 34.7 (CH, cy-Oct), 87.3 (CH-O, cy-Oct), 119.4 (C, Isox), 132.4 (4CH, Ar + 2C(SO_2_)), 142.7 (4C, Ar), 145.3 (2C, Ar).

HRMS (ESI^+^, *m*/*z*): calculated for C_27_H_36_N_2_O_5_S_2_ [M+Na]^+^, 555.1958; found, 555.1958.

#### 3.2.17. *N*-((2,3-Dihydrobenzo[*b*][1,4]dioxin-6-yl)sulfonyl)-*N*-(3a,4,5,6,7,8,9,9a-octahydrocycloocta[*d*]isoxazol-3-yl)-2,3-dihydrobenzo[*b*][1,4]dioxine-6-sulfonamide (**5f**)

Yield 60 mg (59%), method A; reaction time 1 day, 0.54 mmol of sulfonyl chloride was used. White crystals, m.p. 129–130 °C. R*_f_* = 0.22 (DCM).

^1^H NMR (*δ*, ppm; *J*, Hz): 1.35–2.16 (m, 11H, 2CH_2_); 2.17–2.32 (m, 1H, CH_2_); 3.76–3.91 (m, 1H, CH, cy-Oct); 4.15–4.44 (m, 8H, 4CH_2_O); 4.63–4.70 (m, 1H, CH-O, cy-Oct); 6.76 (d, ^3^*J* 8.7, 1H, CH, Ar); 6.77 (d, ^3^*J* 8.7, 1H, CH, Ar); 7.09 (br.d, ^4^*J* 1.2, 1H, CH, Ar); 7.12–7.20 (m, 2H, 2CH, Ar); 7.23 (br.dd, ^3^*J* 8.7, ^4^*J* 1.2, 1H, CH, Ar).

^13^C NMR (*δ*, ppm): 23.4 (CH_2_), 24.2 (CH_2_), 25.2 (CH_2_), 27.5 (CH_2_), 28.0 (CH_2_), 29.9 (CH_2_), 34.6 (CH, cy-Oct), 64.0 (2CH_2_O), 64.8 (2CH_2_O), 89.9 (CH-O, cy-Oct), 117.3 (CH, Ar), 117.4 (CH, Ar), 119.0 (CH, Ar), 119.1 (CH, Ar), 120.5 (C, Isox), 123.4 (CH, Ar), 123.8 (CH, Ar), 126.0 (C(SO_2_)), 126.7 (C(SO_2_)), 143.0 (C, Ar), 143.2 (C, Ar), 149.1 (C, Ar), 149.3 (C, Ar).

HRMS (ESI^+^, *m*/*z*): calculated for C_25_H_28_N_2_O_9_S_2_ [M+Na]^+^, 587.1128; found, 587.1128.

#### 3.2.18. *N*-((2,4-difluorophenyl)sulfonyl)-2,4-difluoro-*N*-(3a,4,5,6,7,8,9,9a-octahydrocycloocta[*d*]isoxazol-3-yl)benzenesulfonamide (**5g**)

Yield 92 mg (98%), method A; reaction time 1 day, 0.54 mmol of sulfonyl chloride was used. White crystals, m.p. 129–131 °C. R*_f_* = 0.56 (DCM).

^1^H NMR (*δ*, ppm; *J*, Hz): 1.34–2.18 (m, 11H, 6CH_2_); 2.19–2.34 (m, 1H, CH_2_); 3.62–3.79 (m, 1H, CH, cy-Oct); 4.62–4.82 (m, 1H, CH-O, cy-Oct); 7.00 (ddd, ^3^*J*_HF_ 9.9, ^3^*J*_HF_ 8.6, ^4^*J*_HH_ 2.4, 2H, 2CH, Ar); 7.04–7.16 (m, 2H, 2CH, Ar); 8.02 (br.s, 2H, 2CH, Ar).

^13^C NMR (*δ*, ppm; *J*, Hz): 23.3 (CH_2_), 24.0 (CH_2_), 25.3 (CH_2_), 27.6 (CH_2_), 28.7 (CH_2_), 29.3 (CH_2_), 34.5 (CH, cy-Oct), 89.0 (CH-O, cy-Oct), 106.2 (dd, ^2^*J*_CF_ 26, ^2^*J*_CF_ 26, 2CH, Ar), 112.5 (dd, ^2^*J*_CF_ 22, ^4^*J*_CF_ 3, 2CH, Ar), 119.1–119.6 (m, C(SO_2_)), 119.8 (C, Isox), 120.0–120.3 (m, C(SO_2_)), 134.9–135.8 (m, 2CH, Ar), 161.3 (m, ^1^*J*_CF_ 267, 2CF, Ar), 167.7 (dd, ^1^*J*_CF_ 262, ^3^*J*_CF_ 12, 2CF, Ar).

NMR ^19^F (*δ*, ppm): −99.65 (br.s, 1F, CF, Ar); −98.32 (br.s, 1F, CF, Ar); −94.96 (br.s, 1F, CF, Ar); −94.87 (br.s, 1F, CF, Ar).

HRMS (ESI^+^, *m*/*z*): calculated for C_21_H_20_F_4_N_2_O_5_S_2_ [M+Na]^+^, 543.0642; found, 543.0642.

#### 3.2.19. 2-Nitro-*N*-((2-nitrophenyl)sulfonyl)-*N*-(3a,4,5,6,7,8,9,9a-octahydrocycloocta[*d*]isoxazol-3-yl)benzenesulfonamide (**5h**)

Yield 47 mg (49%), method A; reaction time 9 h, 0.45 mmol of sulfonyl chloride was used. White crystals, m.p. 153–154 °C. R*_f_* = 0.58 (petroleum ether:EtOAc = 1:1).

^1^H NMR (*δ*, ppm; *J*, Hz): 1.42–2.14 (m, 11H, 6CH_2_); 2.16–2.28 (m, 1H, CH_2_); 3.62–3.73 (m, 1H, CH, cy-Oct); 4.70–4.77 (m, 1H, CH-O, cy-Oct); 7.74 (br.d, ^3^*J* 7.7, 2H, 2CH, Ar); 7.78–7.93 (m, 4H, 4CH, Ar); 8.27–8.44 (m, 2H, 2CH, Ar).

^13^C NMR (*δ*, ppm): 23.3 (CH_2_), 24.4 (CH_2_), 25.1 (CH_2_), 27.4 (CH_2_), 27.8 (CH_2_), 29.2 (CH_2_), 35.1 (CH, cy-Oct), 89.0 (CH-O, cy-Oct), 120.2 (C, Isox), 124.5 (2CH, Ar), 128.1 (2C(SO_2_)), 132.3 (2CH, Ar), 134.0 (2CH, Ar), 136.5 (2CH, Ar), 149.0 (2C(NO_2_)).

HRMS (ESI^+^, *m*/*z*): calculated for C_21_H_22_N_4_O_9_S_2_ [M+Na]^+^, 561.0720; found, 561.0713.

#### 3.2.20. 4-Nitro-*N*-((4-nitrophenyl)sulfonyl)-*N*-(3a,4,5,6,7,8,9,9a-octahydrocycloocta[*d*]isoxazol-3-yl)benzenesulfonamide (**5i**)

Yield 95 mg (98%), method A; reaction time 3 h, 0.36 mmol of sulfonyl chloride was used. White crystals, m.p. 139–140 °C. R*_f_* = 0.32 (petroleum ether:EtOAc = 4:1).

^1^H NMR (*δ*, ppm): 1.38–2.18 (m, 11H, 6CH_2_); 2.18–2.31 (m, 1H, CH_2_); 3.61–3.68 (m, 1H, CH, cy-Oct); 4.57–4.69 (m, 1H, CH-O, cy-Oct); 8.04–8.23 (m, 4H, 4CH, Ar); 8.27–8.44 (m, 4H, 4CH, Ar).

^13^C NMR (*δ*, ppm): 23.7 (CH_2_), 24.4 (CH_2_), 25.2 (CH_2_), 27.35 (CH_2_), 27.40 (CH_2_), 29.2 (CH_2_), 34.7 (CH, cy-Oct), 90.0 (CH-O, cy-Oct), 120.0 (C, Isox), 124.3 (4CH, Ar), 131.4 (2CH, Ar), 131.6 (2CH, Ar), 139.9 (C(SO_2_)), 140.7 (C(SO_2_)), 151.5 (2C(NO_2_), Ar).

HRMS (ESI^+^, *m*/*z*): calculated for C_21_H_22_N_4_O_9_S_2_ [M+Na]^+^, 561.0720; found, 561.0713.

#### 3.2.21. *N*-(Naphthalen-2-ylsulfonyl)-*N*-(3a,4,5,6,7,8,9,9a-octahydrocycloocta[*d*]isoxazol-3-yl)naphthalene-2-sulfonamide (**5j**)

Yield 51 mg (52%), method A; reaction time 9 h, 0.45 mmol of sulfonyl chloride was used. White crystals, m.p. 160–162 °C. R*_f_* = 0.44 (DCM).

^1^H NMR (*δ*, ppm): 1.42–2.23 (m, 11H, 6CH_2_); 2.35–2.45 (m, 1H, CH_2_); 3.93–3.99 (m, 1H, CH, cy-Oct); 4.79–4.86 (m, 1H, CH-O, cy-Oct); 7.30–7.41 (m, 4H, 4CH, Ar); 7.42–7.62 (m, 8H, 8CH, Ar); 7.97 (br.s, 1H, CH, Ar); 8.14 (br.s, 1H, CH, Ar).

^13^C NMR (*δ*, ppm): 23.6 (CH_2_), 24.3 (CH_2_), 25.3 (CH_2_), 27.5 (CH_2_), 28.0 (CH_2_), 29.9 (CH_2_), 34.8 (CH, cy-Oct), 90.4 (CH-O, cy-Oct), 120.6 (C, Isox), 123.1 (CH, Ar), 123.5 (CH, Ar), 127.6 (2CH, Ar), 127.8 (CH, Ar), 127.9 (CH, Ar), 128.6 (CH, Ar), 128.9 (CH, Ar), 129.4 (2CH, Ar), 129.7 (CH, Ar), 129.8 (CH, Ar), 130.5 (C, Ar), 131.1 (C, Ar), 131.3 (C, Ar), 131.4 (CH, Ar + C, Ar), 131.5 (CH, Ar), 135.3 (C(SO_2_)), 135.4 (C(SO_2_)).

HRMS (ESI^+^, *m*/*z*): calculated for C_29_H_28_N_2_O_5_S_2_ [M+H]^+^, 549.1512; found, 549.1504.

#### 3.2.22. *N*-(3a,4,5,6,7,8,9,9a-Octahydrocycloocta[*d*]isoxazol-3-yl)-*N*-(thiophen-2-ylsulfonyl)thiophene-2-sulfonamide (**5k**)

Yield 54 mg (65%), method A; reaction time 1 day, 0.54 mmol of sulfonyl chloride was used. White crystals, m.p. 131–132 °C. R*_f_* = 0.47 (petroleum ether:EtOAc = 1:1).

^1^H NMR (*δ*, ppm): 1.41–2.16 (m, 11H, 6CH_2_); 2.24–2.36 (m, 1H, CH_2_); 3.77–3.84 (m, 1H, CH, cy-Oct); 4.70–4.77 (m, 1H, CH-O, cy-Oct); 6.92–7.05 (m, 2H, 2CH, Tioph); 7.54–7.60 (m, 2H, 2CH, Tioph); 7.62–7.70 (m, 2H, 2CH, Tioph).

^13^C NMR (*δ*, ppm): 23.5 (CH_2_), 24.1 (CH_2_), 25.2 (CH_2_), 27.5 (CH_2_), 28.1 (CH_2_), 29.8 (CH_2_), 34.8 (CH, cy-Oct), 90.1 (CH-O, cy-Oct), 120.2 (C, Isox), 127.3 (2CH, Tioph), 132.6 (C(SO_2_)), 133.1 (C(SO_2_)), 136.4 (CH, Tioph), 136.5 (CH, Tioph), 137.0 (CH, Tioph), 137.2 (CH, Tioph).

HRMS (ESI^+^, *m*/*z*): calculated for C_17_H_20_N_2_O_5_S_4_ [M+Na]^+^, 483.0147; found, 483.0153.

#### 3.2.23. *N*-(3a,4,5,6,7,8,9,9a-Octahydrocycloocta[*d*]isoxazol-3-yl)-*N*-(pyridin-3-ylsulfonyl)pyridine-3-sulfonamide (**5l**)

Yield 63 mg (78%), method A; reaction time 9 h, 0.45 mmol of sulfonyl chloride was used. White crystals, m.p. 150–151 °C. R*_f_* = 0.46 (DCM:MeOH = 1:0.05).

^1^H NMR (*δ*, ppm): 1.40–2.18 (m, 11H, 6CH_2_); 2.18–2.31 (m, 1H, CH_2_); 3.71–3.78 (m, 1H, CH, cy-Oct); 4.67–4.75 (m, 1H, CH-O, cy-Oct); 7.35–7.48 (m, 2H, 2CH, Py); 8.05–8.25 (m, 2H, 2CH, Py); 8.74–8.95 (m, 4H, 4CH, Py).

^13^C NMR (*δ*, ppm): 23.6 (CH_2_), 24.3 (CH_2_), 25.2 (CH_2_), 27.4 (CH_2_), 27.7 (CH_2_), 29.5 (CH_2_), 34.7 (CH, cy-Oct), 90.3 (CH-O, cy-Oct), 120.1 (C, Isox), 123.8 (2CH, Py), 130.9 (C(SO_2_)), 131.6 (C(SO_2_)), 137.3 (CH, Py), 137.8 (CH, Py), 149.7 (2CH, Py), 155.3 (2CH, Py).

HRMS (ESI^+^, *m*/*z*): calculated for C_19_H_22_N_4_O_5_S_2_ [M+H]^+^, 451.1104; found, 451.1105.

### 3.3. Biology

#### 3.3.1. Cells and Viruses

Porcine embryo kidney (PEK) cells and African green monkey kidney (Vero) cells were obtained from the collection of FSASI “Chumakov FSC R&D IBP RAS” (Institute of Poliomyelitis), Moscow, Russia. PEK cells were grown in the mixture of medium 199 with Earl’s salts and medium 199 with Hanks’ salts (FSASI “Chumakov FSC R&D IBP RAS” (Institute of Poliomyelitis), Moscow, Russia), supplemented with 5% of heat-inactivated fetal bovine serum (Gibco, Grand Island, NY, USA) and penicillin-streptomycin (Paneco-ltd, Moscow, Russia). Vero cells were grown in DMEM with L-glutamine (FSASI “Chumakov FSC R&D IBP RAS” (Institute of Poliomyelitis)) supplemented with 5% of heat-inactivated fetal bovine serum (Gibco, Grand Island, NY, USA) and gentamicin (Paneco-ltd, Moscow, Russia).

Tick-borne encephalitis virus strain Absettarov (GenBank access no. KU885457.1), yellow fever virus strain 17D (Genbank access no. JN628279.1), and West Nile virus strain Strix nebulosa-12 (GenBank access no. OP868929) were taken from the collection of FSASI “Chumakov FSC R&D IBP RAS” (Institute of Poliomyelitis), Moscow, Russia.

#### 3.3.2. Antiviral Activity and Cytotoxicity Assays

Antiviral activity and cytotoxicity of the tested compounds were studied using plaque reduction assay and resazurin cell viability assay, respectively, as described previously [33]. Each compound was tested in at least two independent experiments.

## 4. Conclusions

Two series of novel 3-[*N,N*-bis(sulfonyl)amino]isoxazolines, containing spiro-annulated or 1,2-annulated cyclooctane rings, were synthesized via sulfonylation of corresponding 3-amino- or 3-(sulfonylamino)isoxazolines. Almost all the obtained compounds revealed antiviral activity against tick-borne encephalitis and West Nile viruses. The best results were shown by spirocyclic compounds **4i** (TBEV EC_50_ 2.0 ± 0.5 μM) and **4j** (WNV EC_50_ 1.3 ± 0.5 μM), respectively. It was also found that spiro-isoxazoline **3b** is active against the yellow fever virus with EC_50_ 24 ± 11 μM. On this basis, we can conclude that spirocyclic and bicyclic scaffolds, combining isoxazoline and cyclooctane rings, represent promising structural motifs for the further design of anti-flaviviral agents. The mechanism of action and molecular target of the compounds described above still need to be established and will be the subject of further research.

## Figures and Tables

**Table 1:**
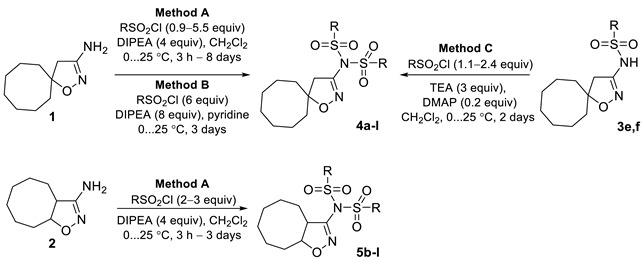
Synthesis of 3-[*N,N*-bis(sulfonyl)amino]isoxazolines **4a–l** and **5b–l**.

R	cmpd	Yield, % ^1^	cmpd	Yield, % ^1^
Me	**4a**	23 (A)	**5a**	-
Bn	**4b**	19 (A)	**5b**	29 (A)
Ph	**4c**	71 (A)	**5c**	78 (A)
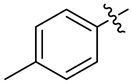	**4d**	56 (A)	**5d**	76 (A)
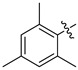	**4e**	60 (C)	**5e**	34 (A)
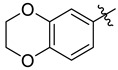	**4f**	36 (A), 61 (C)	**5f**	59 (A)
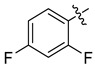	**4g**	47 (A)	**5g**	98 (A)
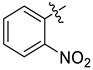	**4h**	20 (B)	**5h**	49 (A)
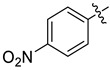	**4i**	57 (A)	**5i**	98 (A)
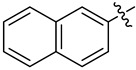	**4j**	72 (A)	**5j**	52 (A)
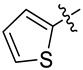	**4k**	32 (A)	**5k**	65 (A)
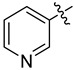	**4l**	92 (A)	**5l**	78 (A)

^1^ Isolated yield.

**Table 2:**
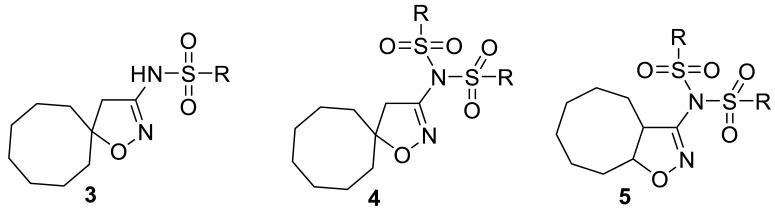
Anti-TBEV activity and chronic cytotoxicity of compounds **3–5**.

R	cmpd	TBEV EC_50_, μM	PEK ^1^ (7 Days) CC_50_, μM	cmpd	TBEV EC_50_, μM	PEK (7 Days) CC_50_, μM	cmpd	TBEV EC_50_, μM	PEK (7 Days) CC_50_, μM
Me	**3a**	>100	>50	**4a**	27 ± 12	>50	-	-	-
Bn	**3b**	13 ± 8	>50	**4b**	>50	>50	**5b**	21.7 ± 2.8	>50
Ph	**3c**	>50	>50	**4c**	9 ± 2	>50	**5c**	>50	>50
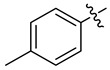	**3d**	>50	>50	**4d**	21 ± 13	>50	**5d**	34 ± 4	>50
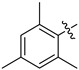	**3e**	18 ± 7	>50	**4e**	12 ± 7	>50	**5e**	10 ± 5	>50
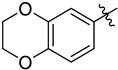	**3f**	>50	>50	**4f**	13 ± 6	>50	**5f**	29 ± 7	>50
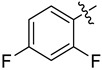	**3g**	>100	>50	**4g**	>50	>50	**5g**	33 ± 15	>50
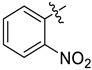	**3h**	28 ± 7	>50	**4h**	20 ± 11	>50	**5h**	12 ± 6	>50
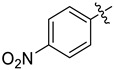	-	-	-	**4i**	2.0 ± 0.5	>50	**5i**	19 ± 4	41.0 ± 1.8
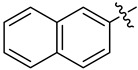	**3j**	20 ± 11	>50	**4j**	17 ± 3	>50	**5j**	10.0 ± 2.4	>50
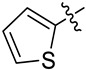	**3k**	>100	>50	**4k**	>50	>50	**5k**	20 ± 3	>50
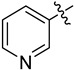	**3l**	>100	>50	**4l**	15 ± 4	>50	**5l**	36 ± 4	>50
	**6**	2.29 ± 0.17	>100						

^1^ Porcine embryo kidney cells.

**Table 3:** Anti-WNV activity and chronic cytotoxicity of compounds **3–5**.

cmpd	WNV EC_50_, μM	Vero ^1^ (7 Days) CC_50_, μM	cmpd	WNV EC_50_, μM	Vero (7 Days) CC_50_, μM	cmpd	WNV EC_50_, μM	Vero (7 Days) CC_50_, μM
**3a**	31.0 ± 2.0	>50	**4a**	10 ± 6	>50	-	-	-
**3b**	9.9 ± 1.5	>50	**4b**	16 ± 8	>50	**5b**	6.3 ± 1.2	>50
**3c**	25 ± 7	>50	**4c**	2.6 ± 1.4	>50	**5c**	18 ± 4	>50
**3d**	14 ± 5	>50	**4d**	3.0 ± 1.9	>50	**5d**	14 ± 8	>50
**3e**	22 ± 4	>50	**4e**	4.8 ± 1.0	>50	**5e**	4.0 ± 0.6	>50
**3f**	18 ± 6	>50	**4f**	7.1 ± 1.0	>50	**5f**	16 ± 10	>50
**3g**	28.4 ± 2.2	>50	**4g**	9.8 ± 2.6	>50	**5g**	12 ± 6	>50
**3h**	21.5 ± 0.9	>50	**4h**	7.8 ± 1.0	33.28 ± 0.02	**5h**	17 ± 3	>50
-	-	-	**4i**	5.3 ± 1.0	28.3 ± 2.4	**5i**	19 ± 11	>50
**3j**	20.3 ± 0.3	31 ± 3	**4j**	1.3 ± 0.5	>50	**5j**	5.9 ± 1.8	>50
**3k**	6.1 ± 1.3	>50	**4k**	10 ± 4	>50	**5k**	18 ± 4	12.0 ± 2.4
**3l**	11 ± 7	>50	**4l**	8 ± 4	>50	**5l**	>50	>50
Favipiravir	102 ± 28	>1000						

^1^ African green monkey kidney cells.

## Data Availability

Not applicable.

## Supplementary Materials

The following supporting information can be downloaded at https://www.mdpi.com/article/10.3390/ijms241310758/s1.
