# Supplementary material for: 3-[N,N-Bis(sulfonyl)amino]isoxazolines with Spiro-Annulated or 1,2-Annulated Cyclooctane Rings Inhibit Reproduction of Tick-Borne Encephalitis, Yellow Fever, and West Nile Viruses
Source: Int J Mol Sci. 2023 Jun 28;24(13):10758. doi: 10.3390/ijms241310758 (PMC10341786; doi:10.3390/ijms241310758)

3-[*N,N*-Bis(sulfonyl)amino]isoxazolines with spiro-annulated or 1,2-annulated cyclooctane rings inhibit reproduction of tick-borne encephalitis, yellow fever, and West Nile viruses

Kseniya N. Sedenkova [1], Artem S. Sazonov [1], Dmitry A. Vasilenko [1], Kristian S. Andriasov [1], Marina G. Eremenko [1], Yuri K. Grishin [1], Evgeny V. Khvatov [2], Alexander S. Goryashchenko [2], Victoria I. Uvarova [2], Aydar A. Ishmukhametov [2,3], Dmitry I. Osolodkin [2,3] and Elena B. Averina [1,*]

[1] Department of Chemistry, Lomonosov Moscow State University, Moscow, Russia
[2] FSASI "Chumakov FSC R&D IBP RAS" (Institute of Poliomyelitis), Moscow, Russia
[3] Institute of Translational Medicine and Biotechnology, Sechenov Moscow State Medical University, Moscow, Russia

* Correspondence: elaver@med.chem.msu.ru

Supplementary materials

1. Acute toxicity of compounds **3-5**………………………..………………………………………….. 2

2. Copies of NMR spectra……………………………………………………………………….…... 3

Table S1. Acute toxicity of compounds **3-5**.

**Compound**	**CC_{50} Vero 24h, µM**	**CC_{50} PEK 24h, µM**
**6**	>100	>100
**Favipiravir**	>1000	>1000
**3a**	>50	>50
**3b**	>50	>50
**3c**	>50	>50
**3d**	>50	>50
**3e**	>50	>50
**3f**	>50	>50
**3g**	>50	>50
**3h**	>50	>50
**3j**	40 ± 3	>50
**3k**	>50	>50
**3l**	>50	>50
**4a**	>50	>50
**4b**	>50	>50
**4c**	>50	>50
**4d**	>50	>50
**4e**	>50	>50
**4f**	>50	>50
**4g**	>50	>50
**4h**	>50	>50
**4i**	35 ± 4	>50
**4j**	>50	>50
**4k**	>50	>50
**4l**	>50	>50
**5b**	>50	>50
**5c**	>50	>50
**5d**	>50	>50
**5e**	>50	>50
**5f**	>50	>50
**5g**	>50	>50
**5h**	39 ± 8	>50
**5i**	>50	>50
**5j**	>50	>50
**5k**	14.9 ± 1.5	>50
**5l**	>50	>50

^{1}H NMR spectrum of compound **4a** ($CDCl_3$)

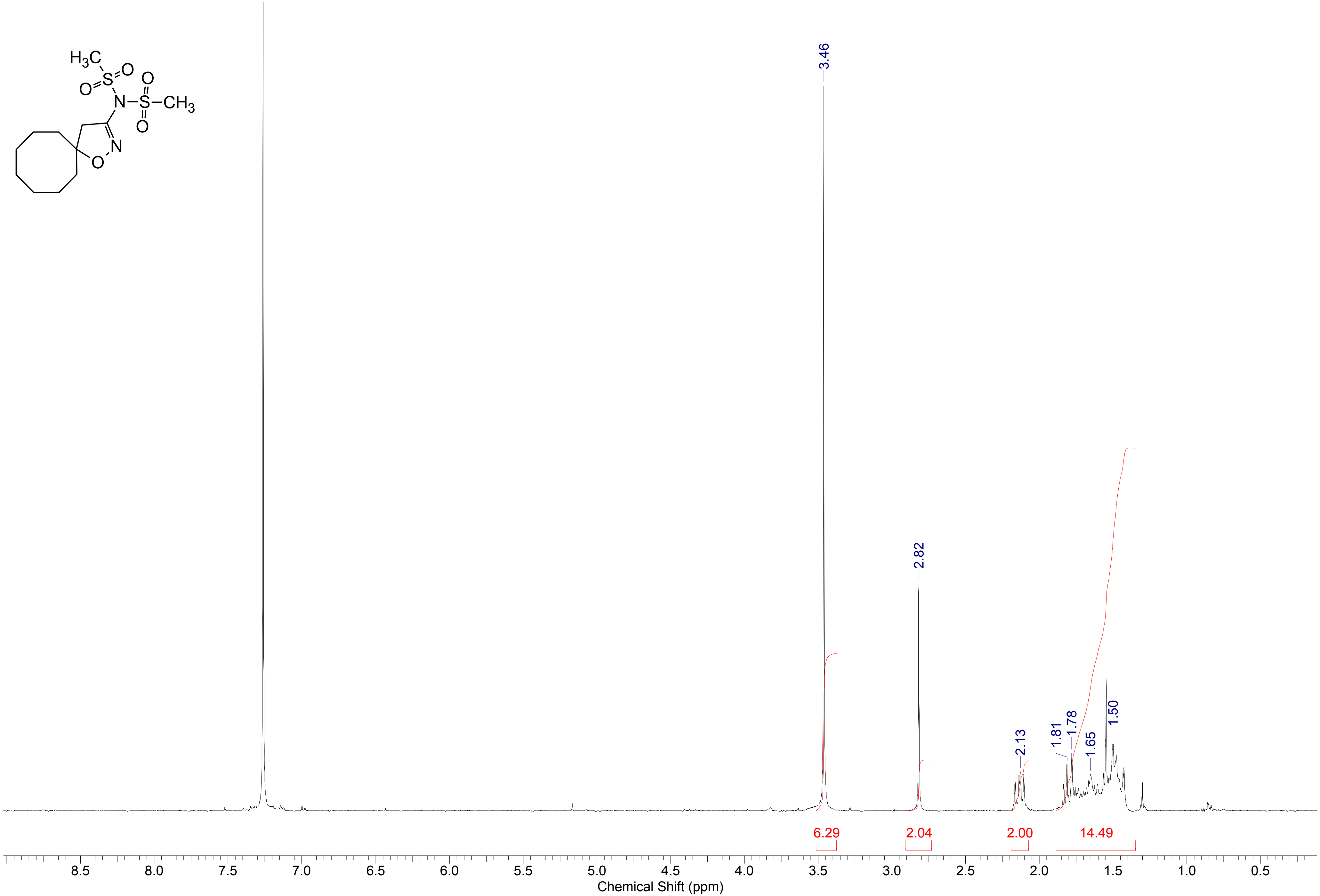

^{13}C NMR spectrum of compound **4a** ($CDCl_3$)

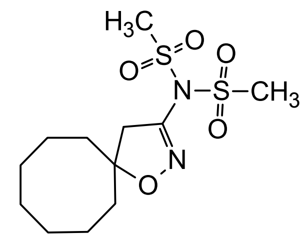

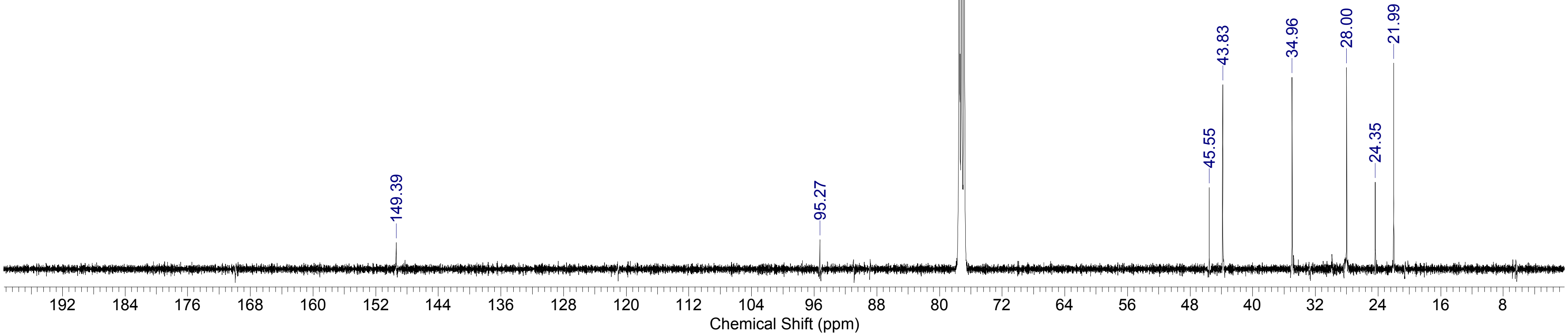

^{1}H NMR spectrum of compound **4b** ($CDCl_3$)

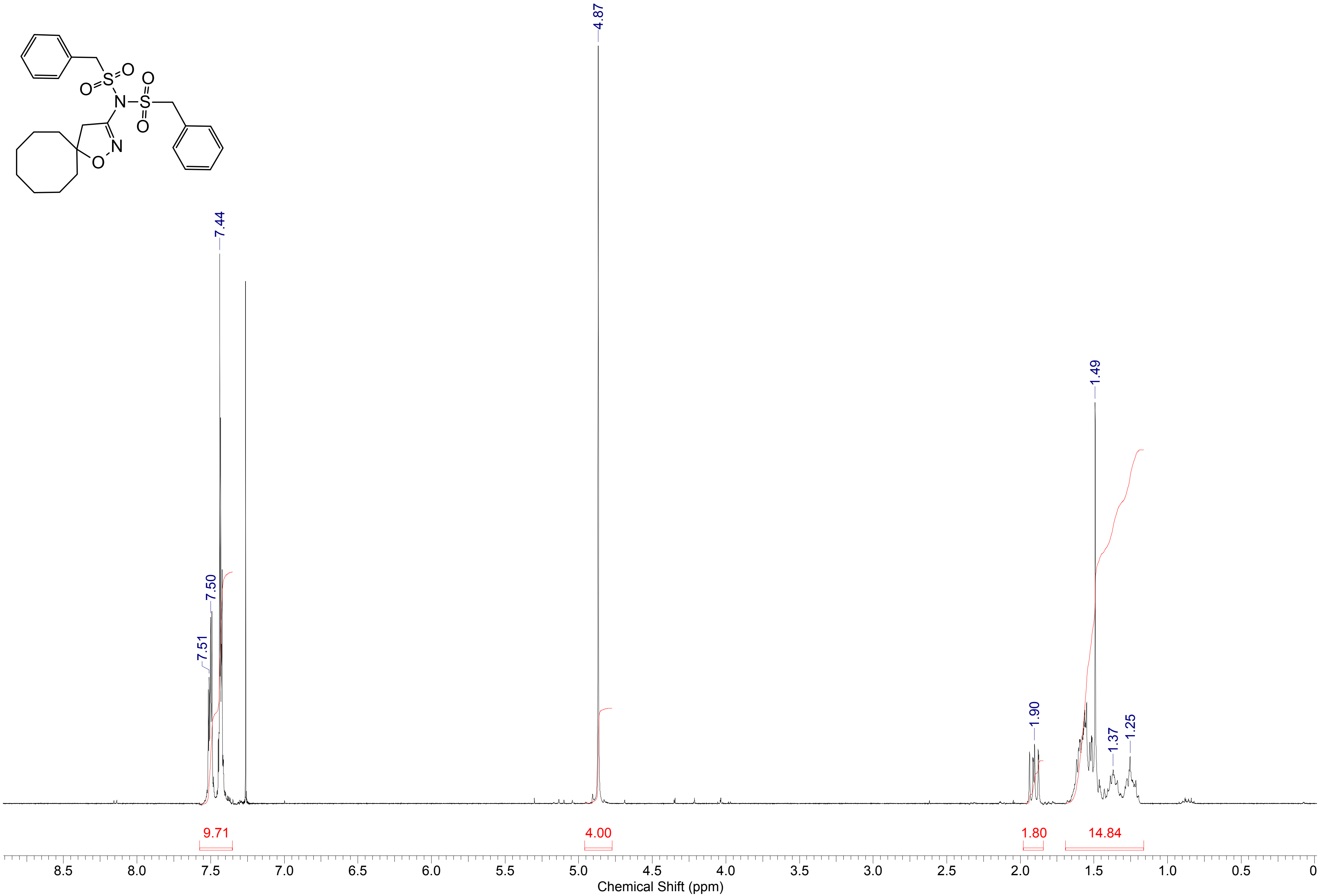

^{13}C NMR spectrum of compound **4b** ($CDCl_3$)

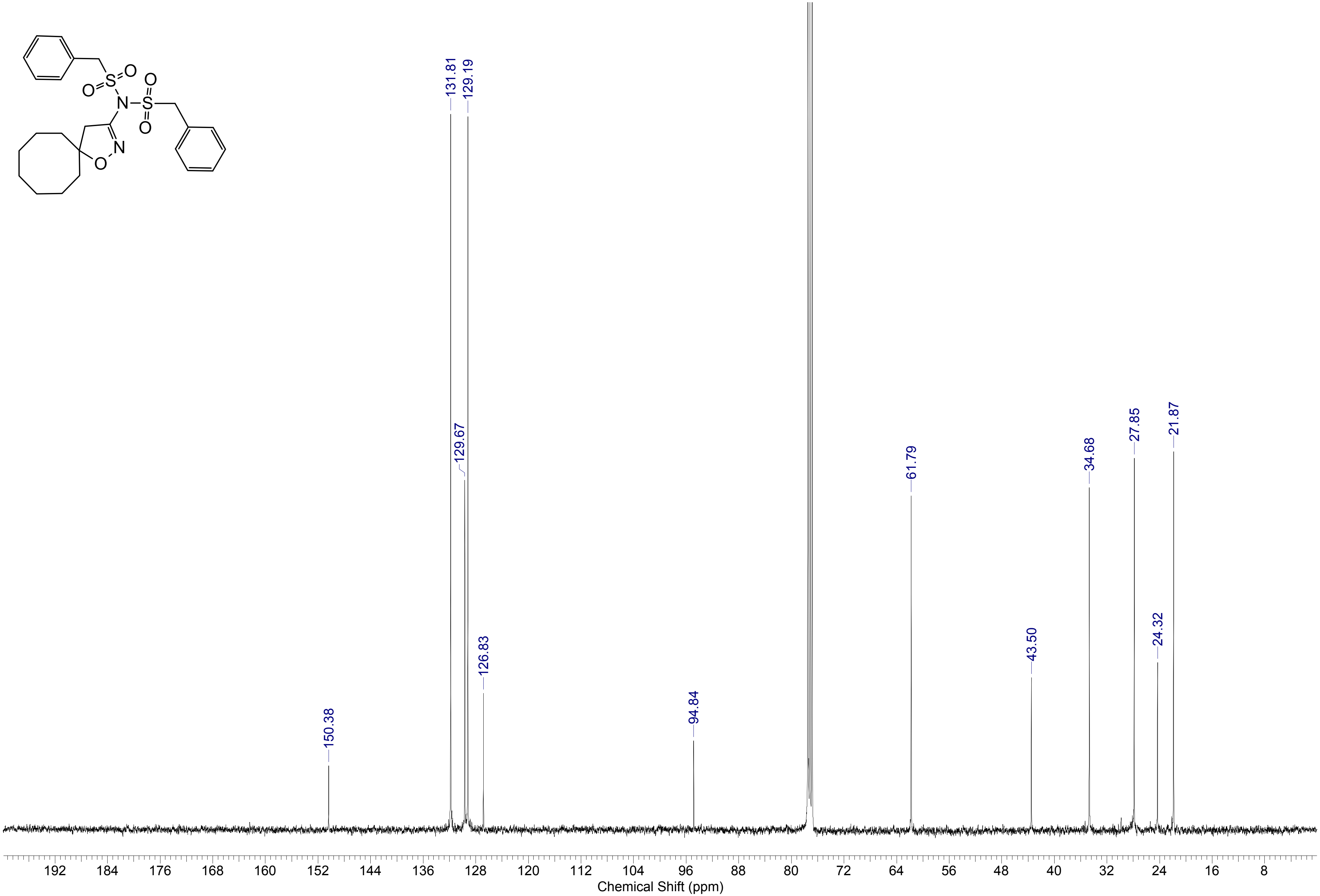

HSQC NMR spectrum of compound **4b** ($CDCl_3$)

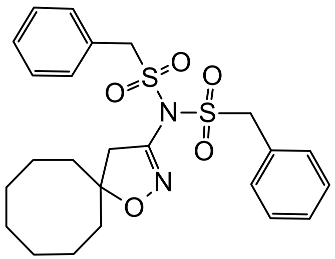

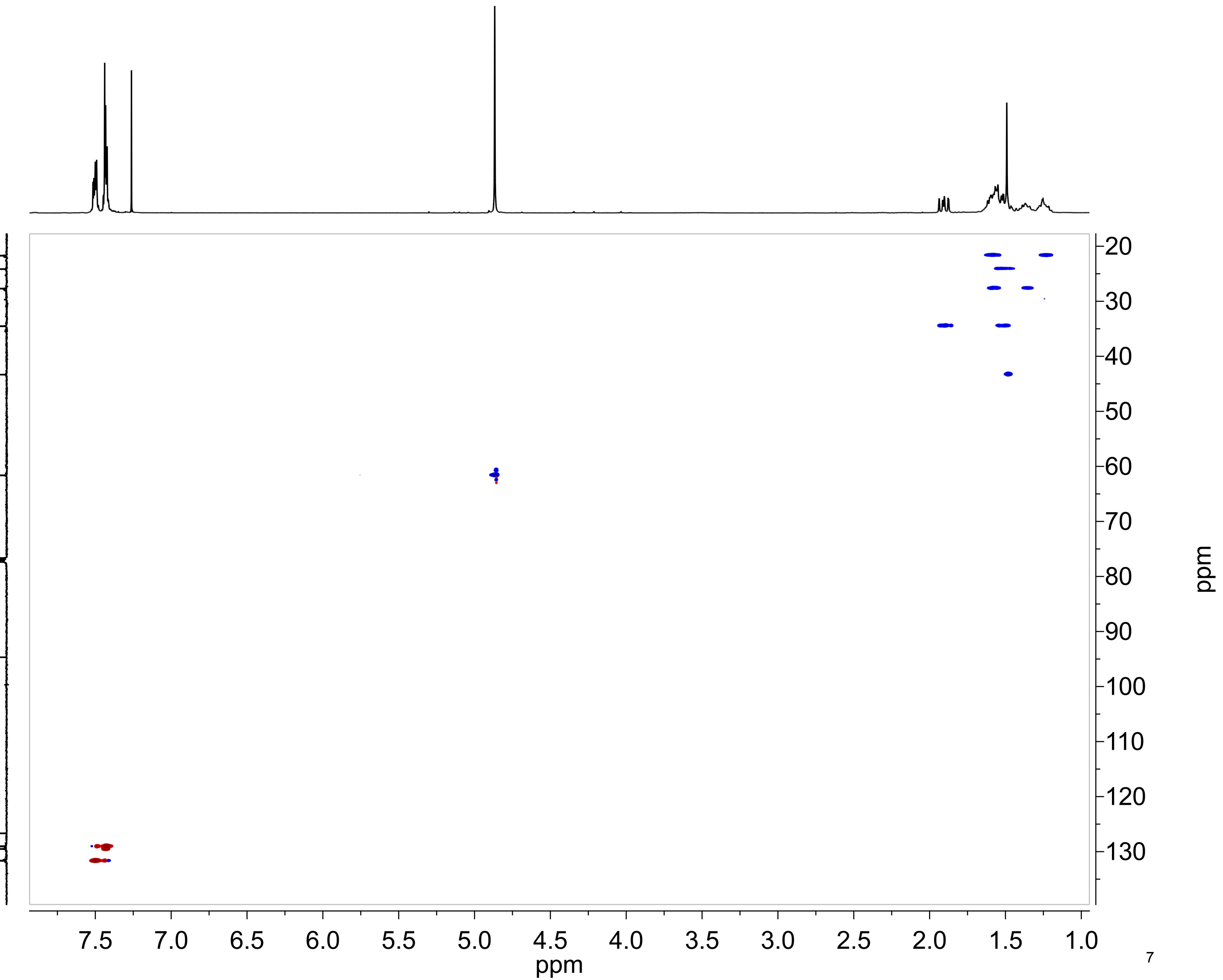

HMBC NMR spectrum of compound **4b** ($CDCl_3$)

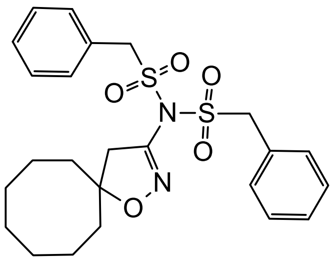

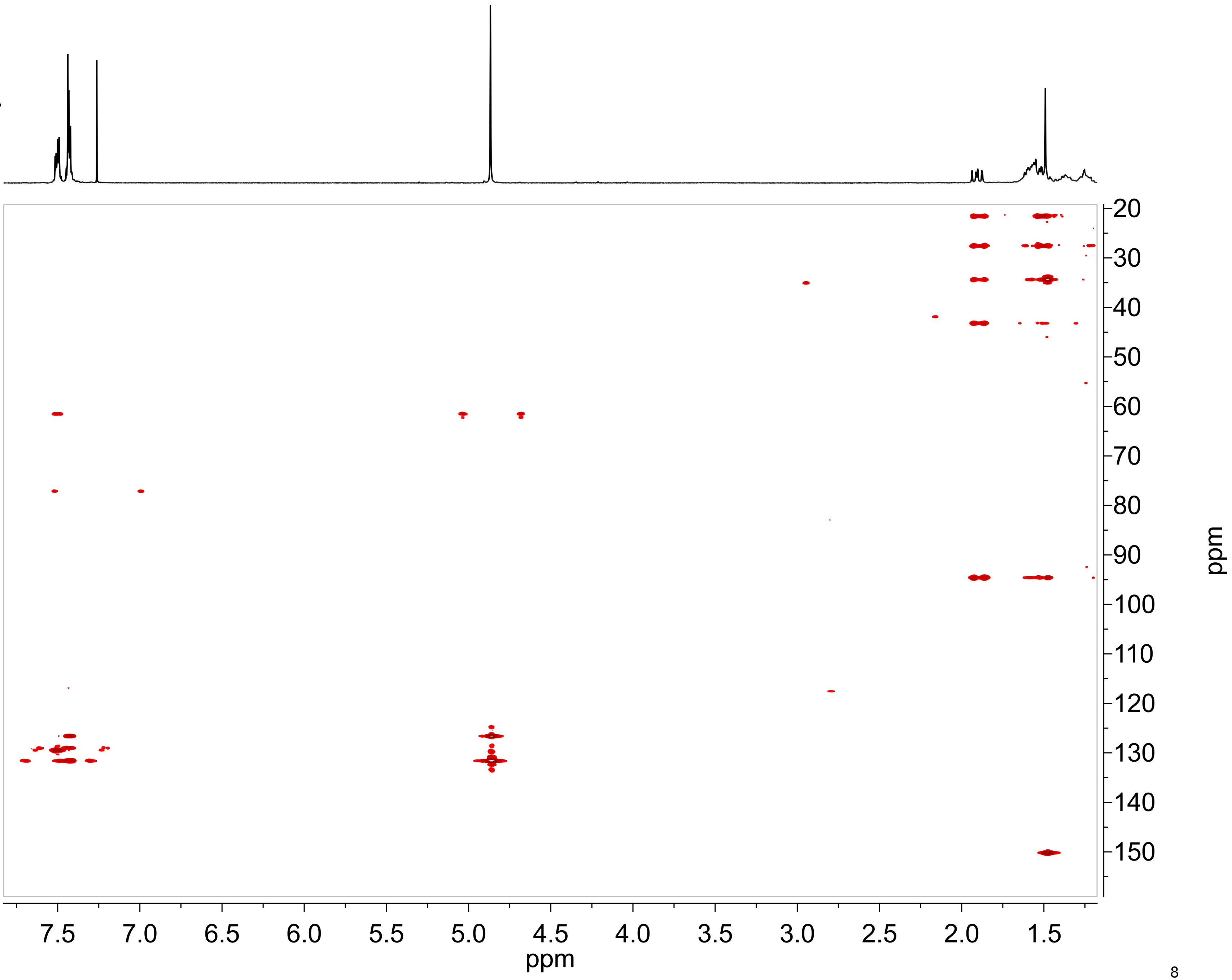

^{1}H NMR spectrum of compound **4c** ($CDCl_3$)

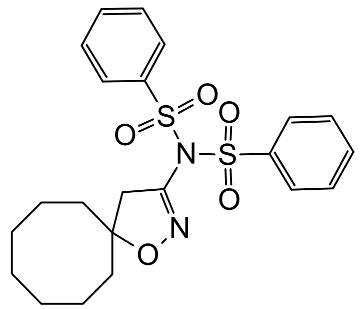

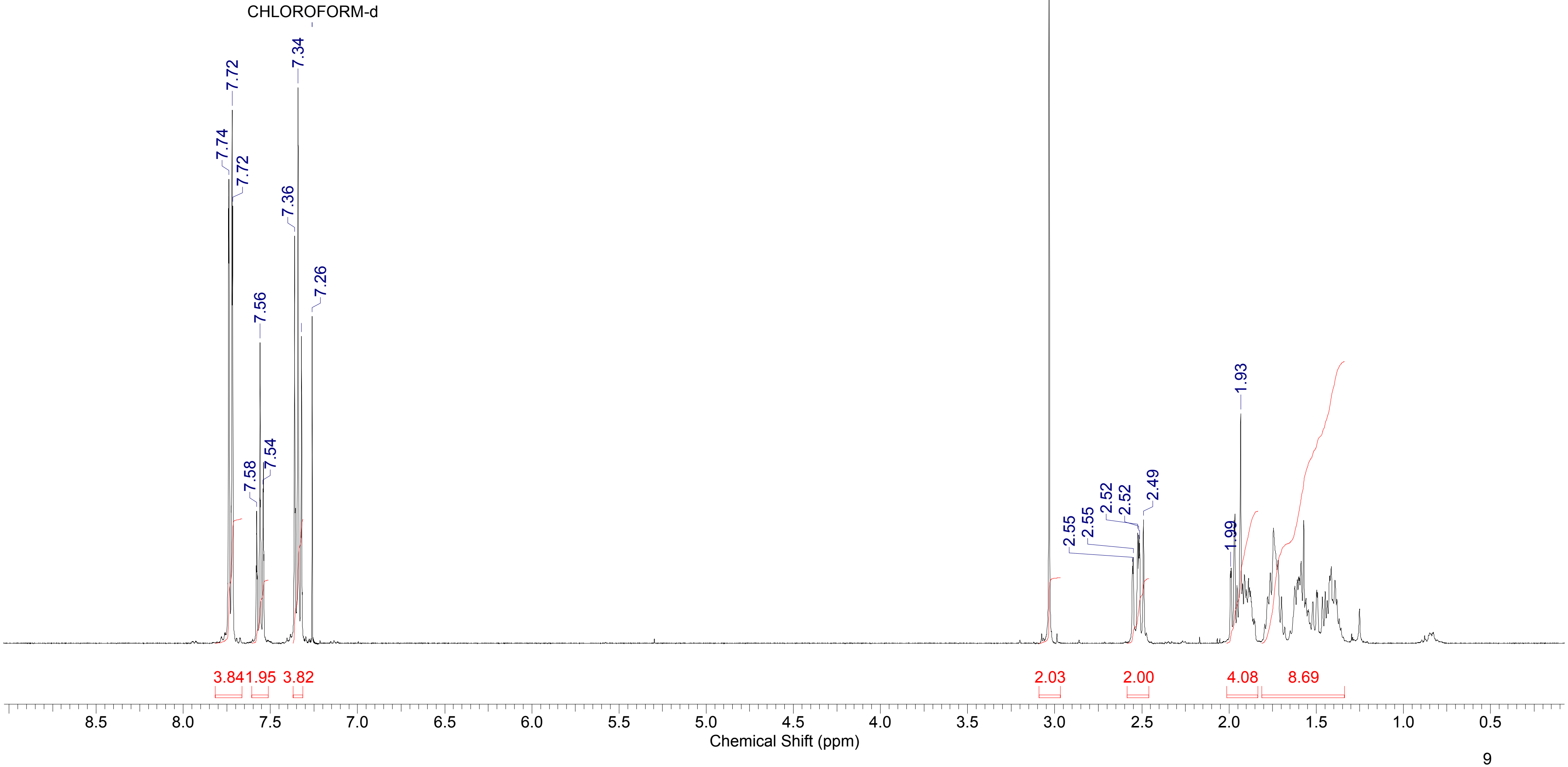

^{13}C NMR spectrum of compound **4c** ($CDCl_3$)

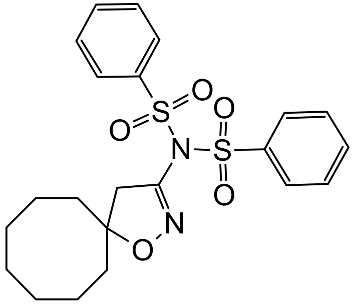

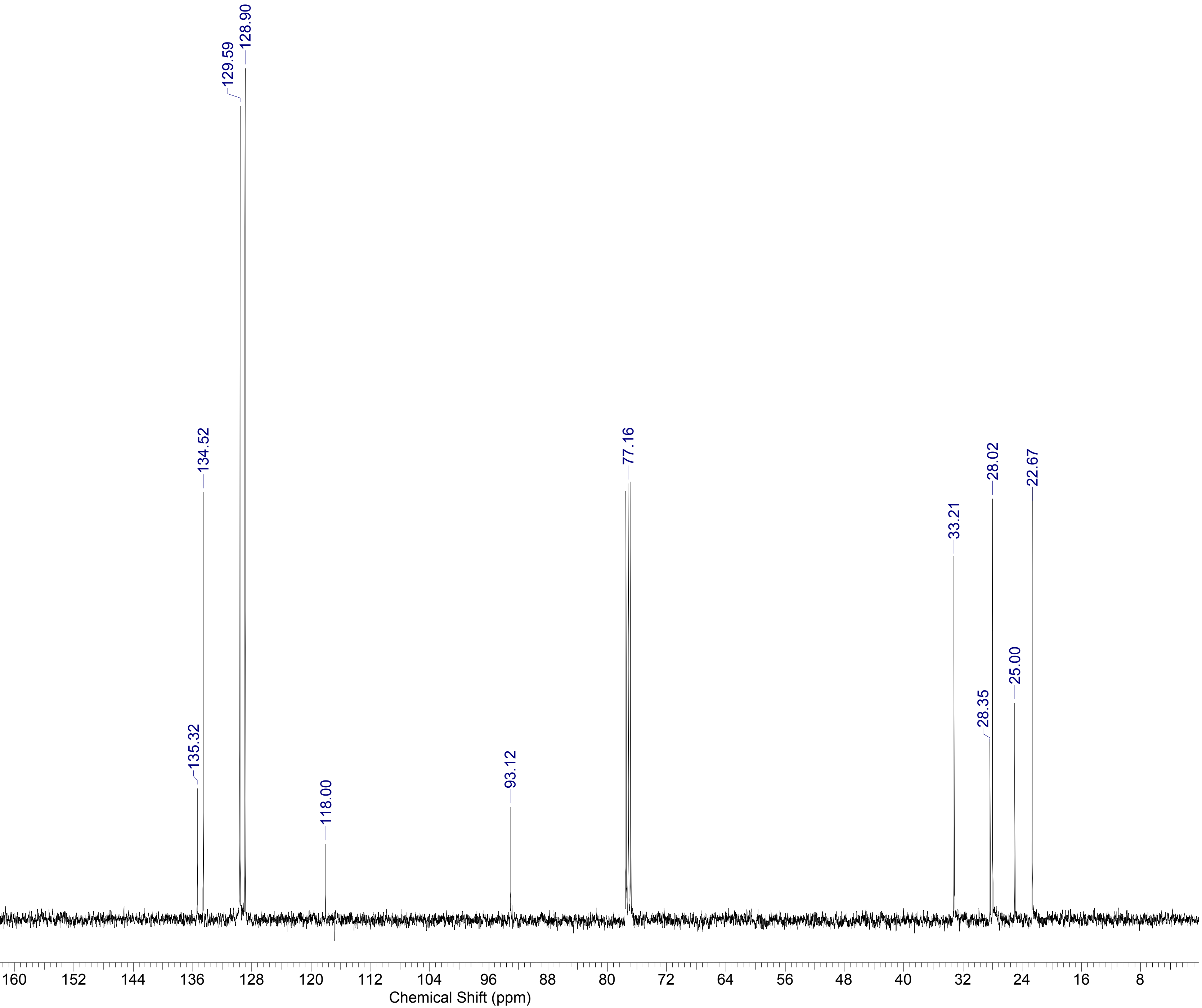

^{1}H NMR spectrum of compound **4d** ($CDCl_3$)

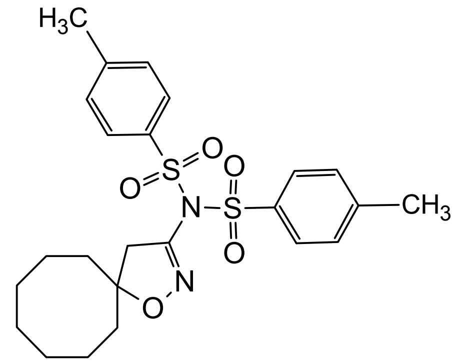

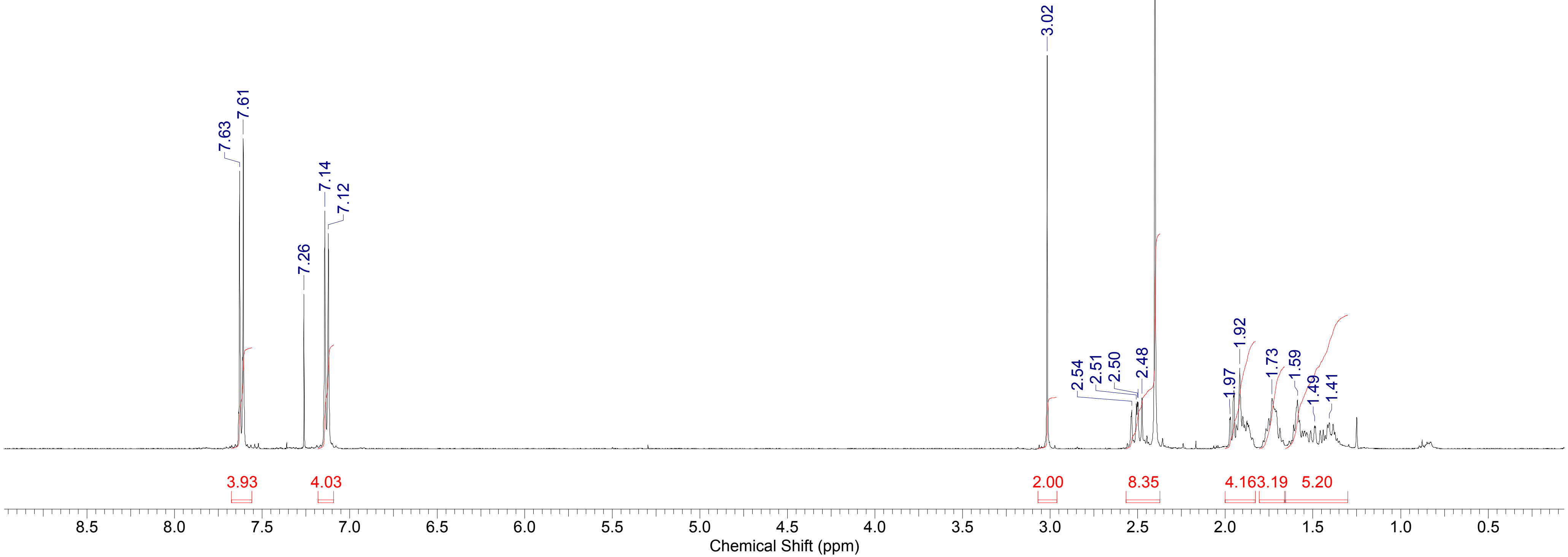

^{13}C NMR spectrum of compound **4d** ($CDCl_3$)

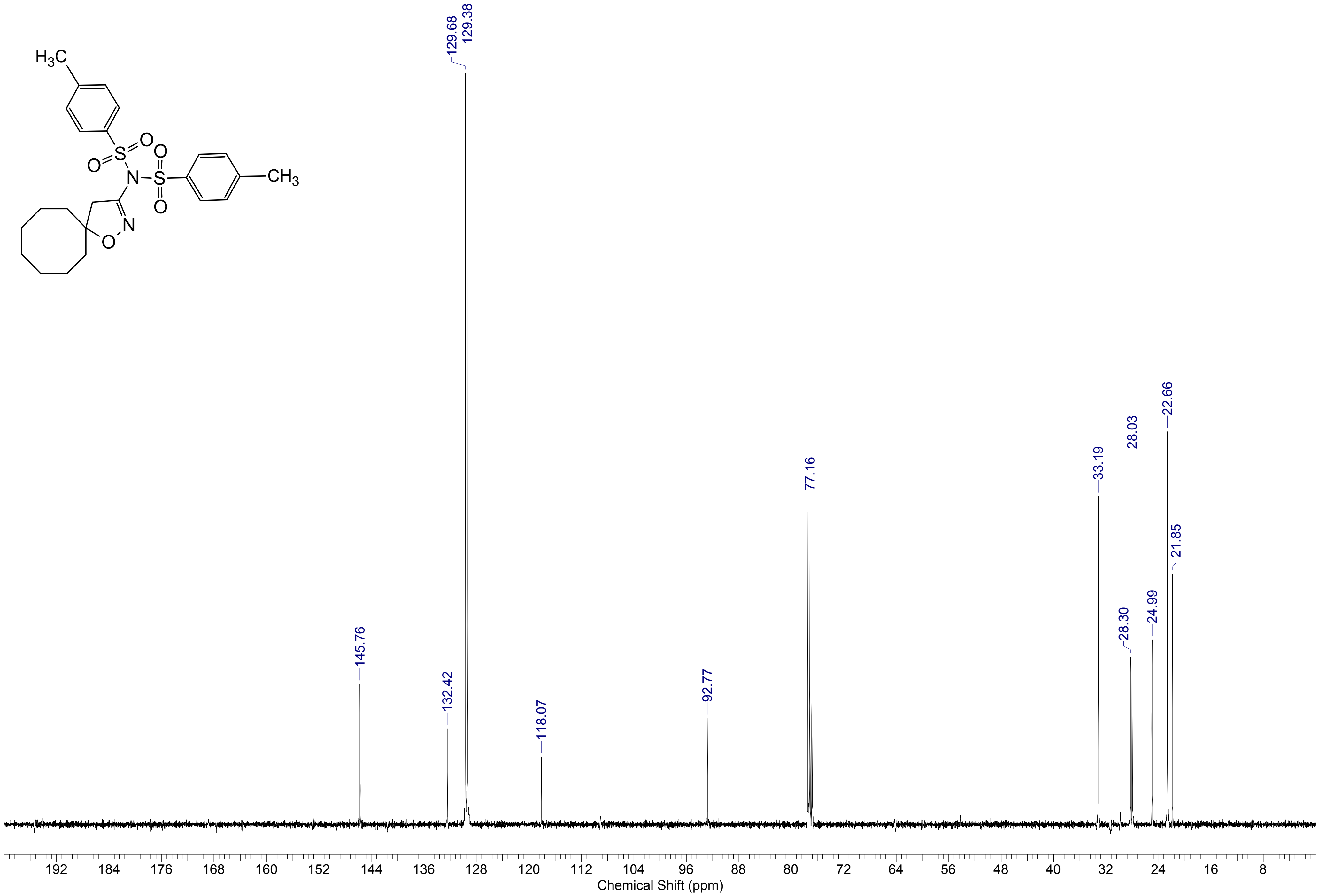

HSQC NMR spectrum of compound **4d** ($CDCl_3$)

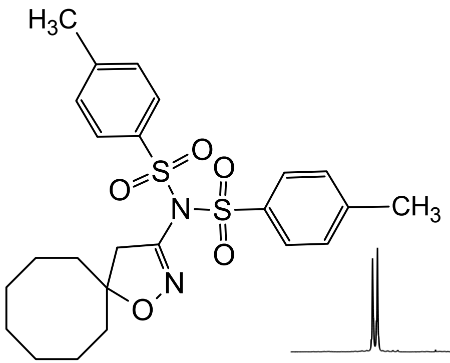

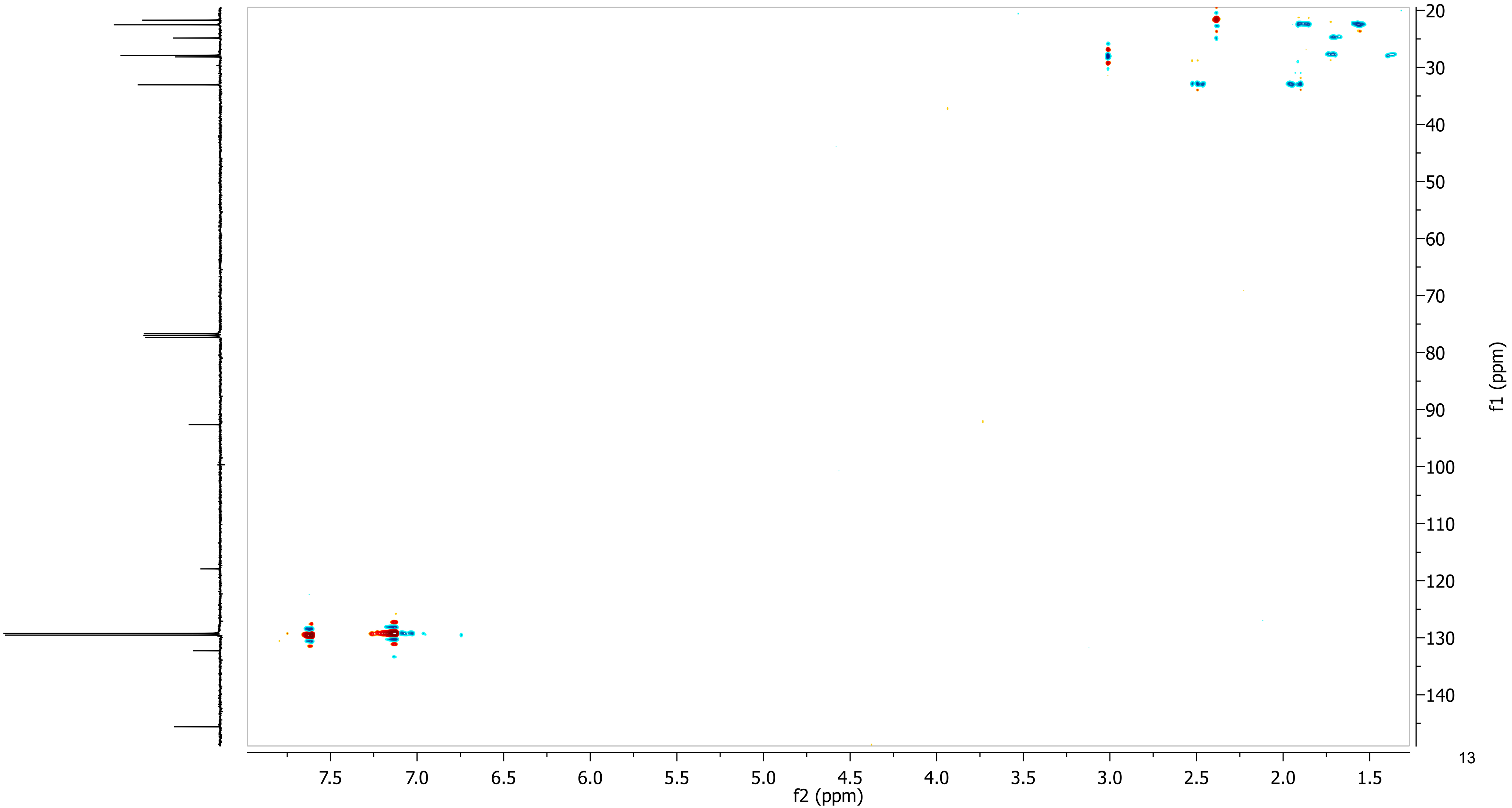

HMBC NMR spectrum of compound **4d** ($CDCl_3$)

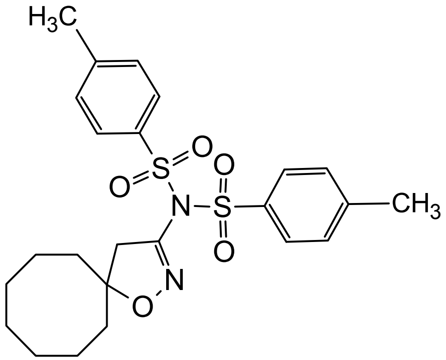

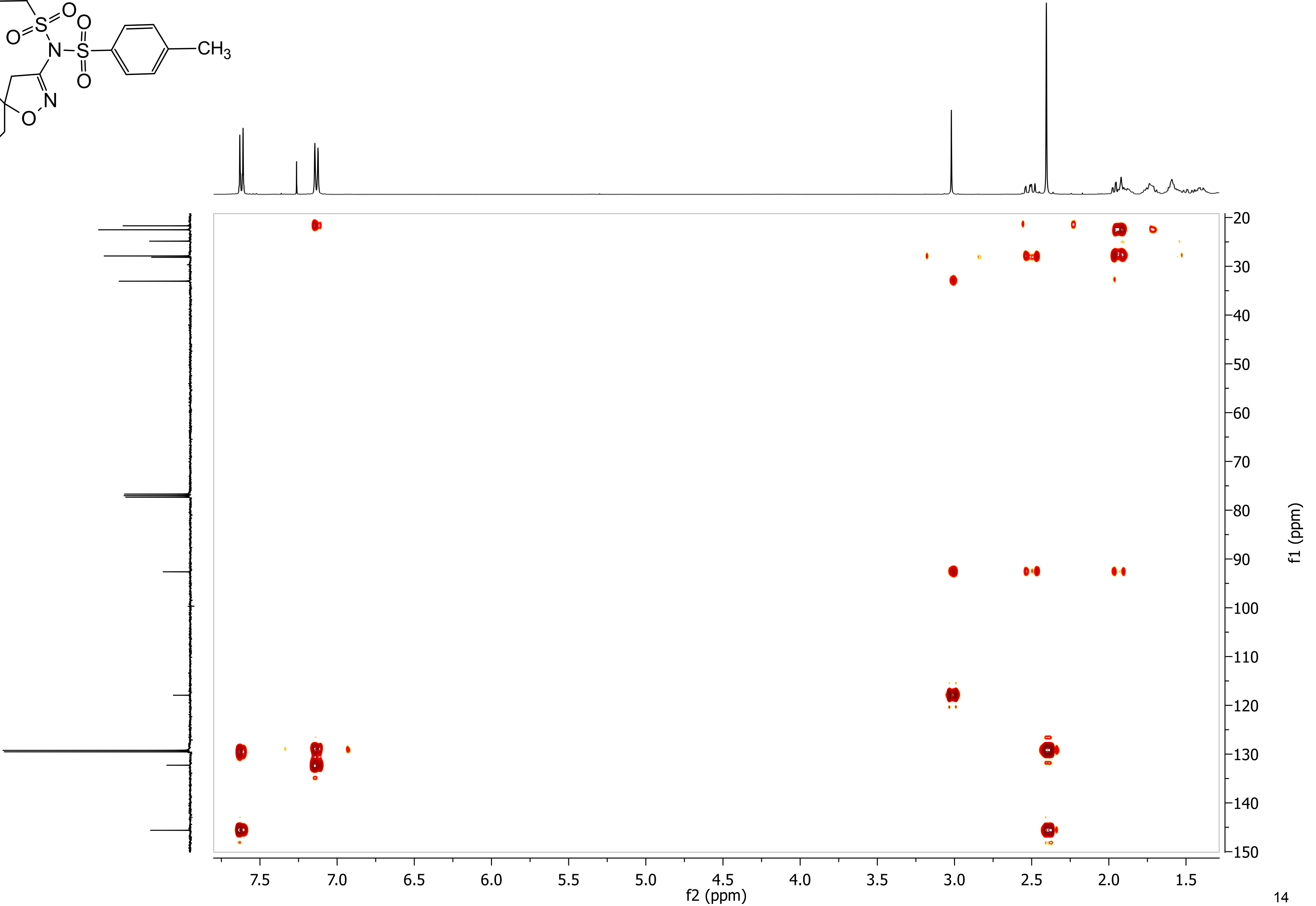

^{1}H NMR spectrum of compound **4e** ($CDCl_3$)

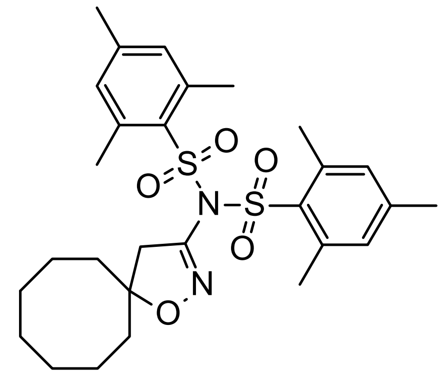

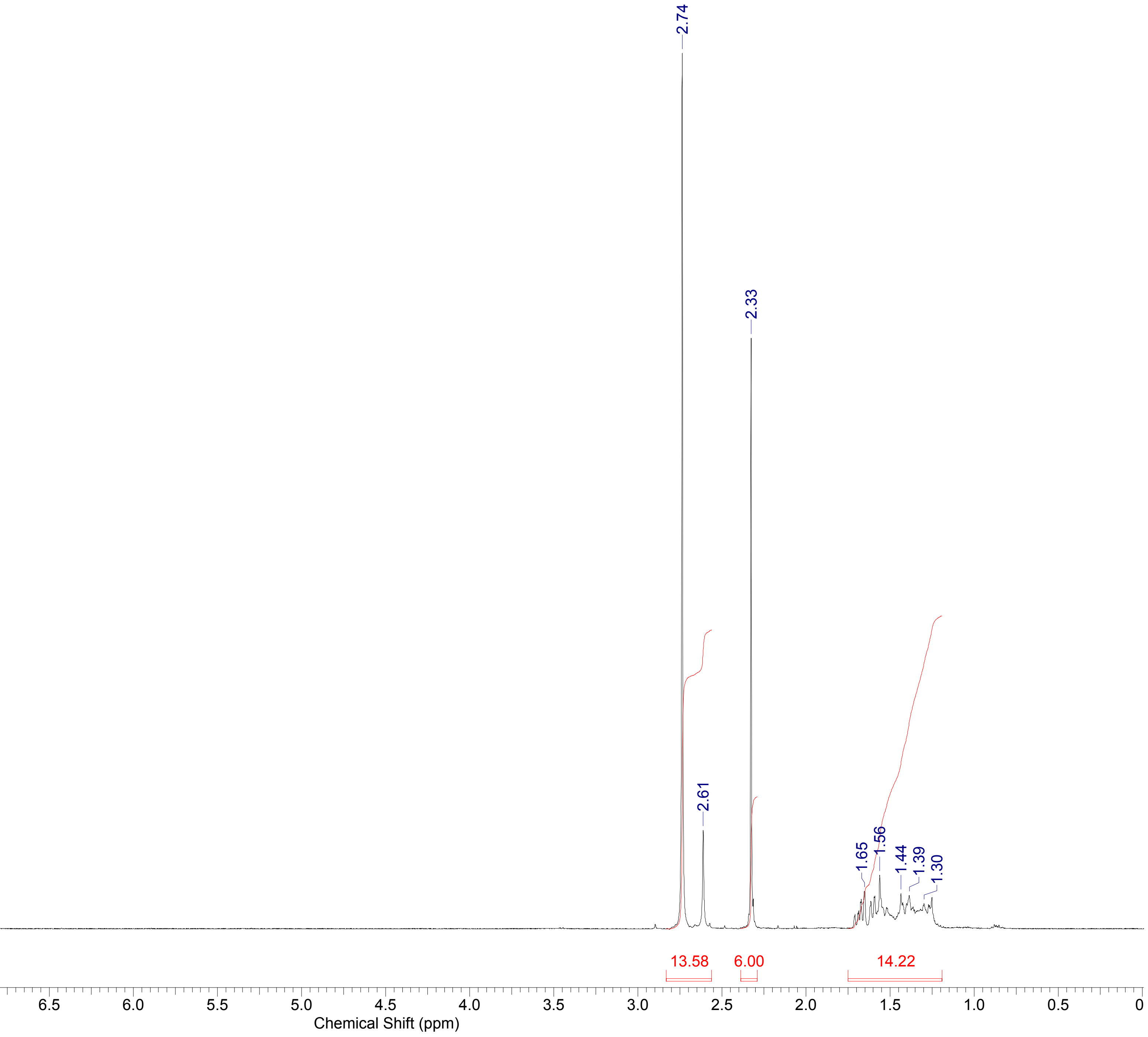

^{13}C NMR spectrum of compound **4e** ($CDCl_3$)

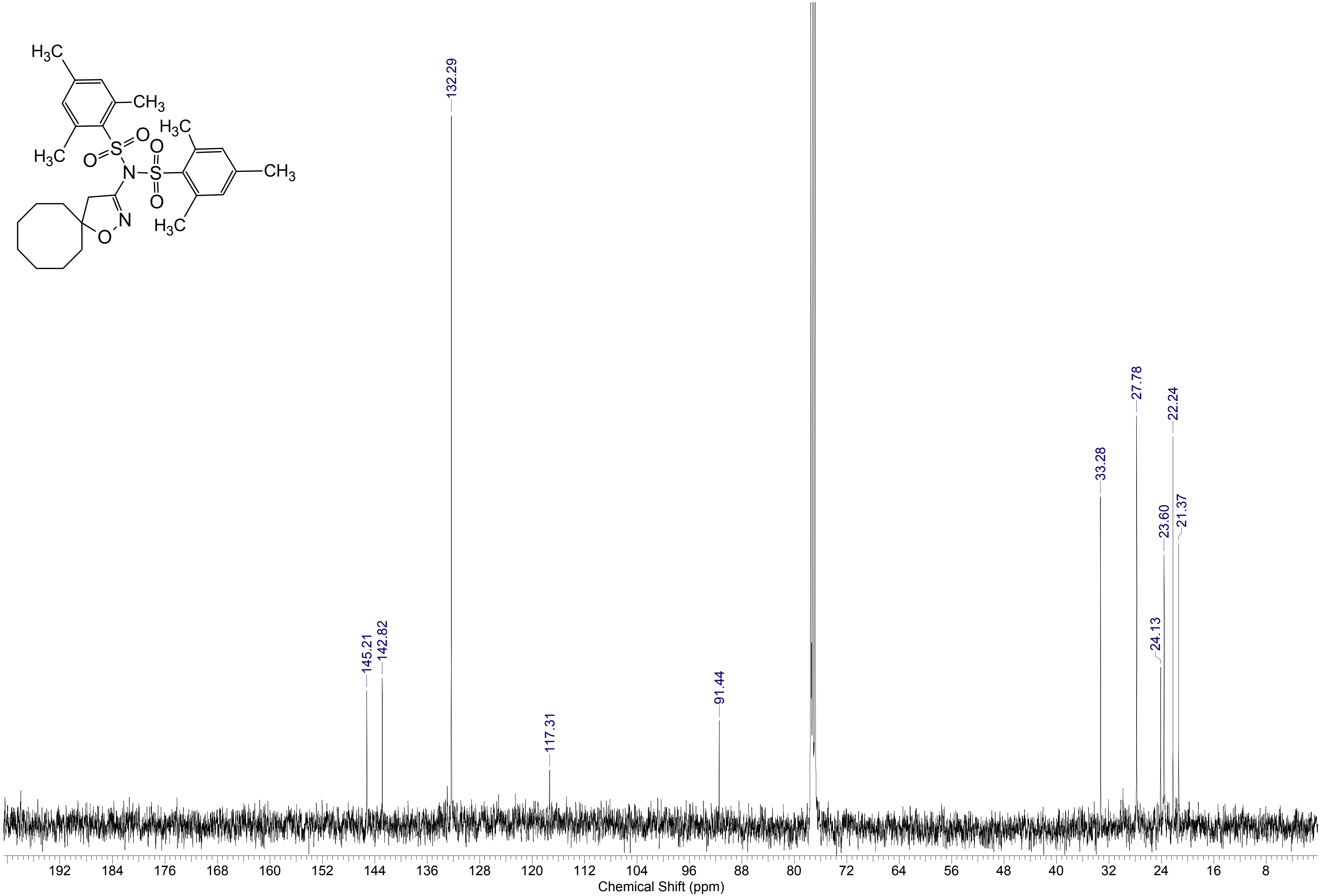

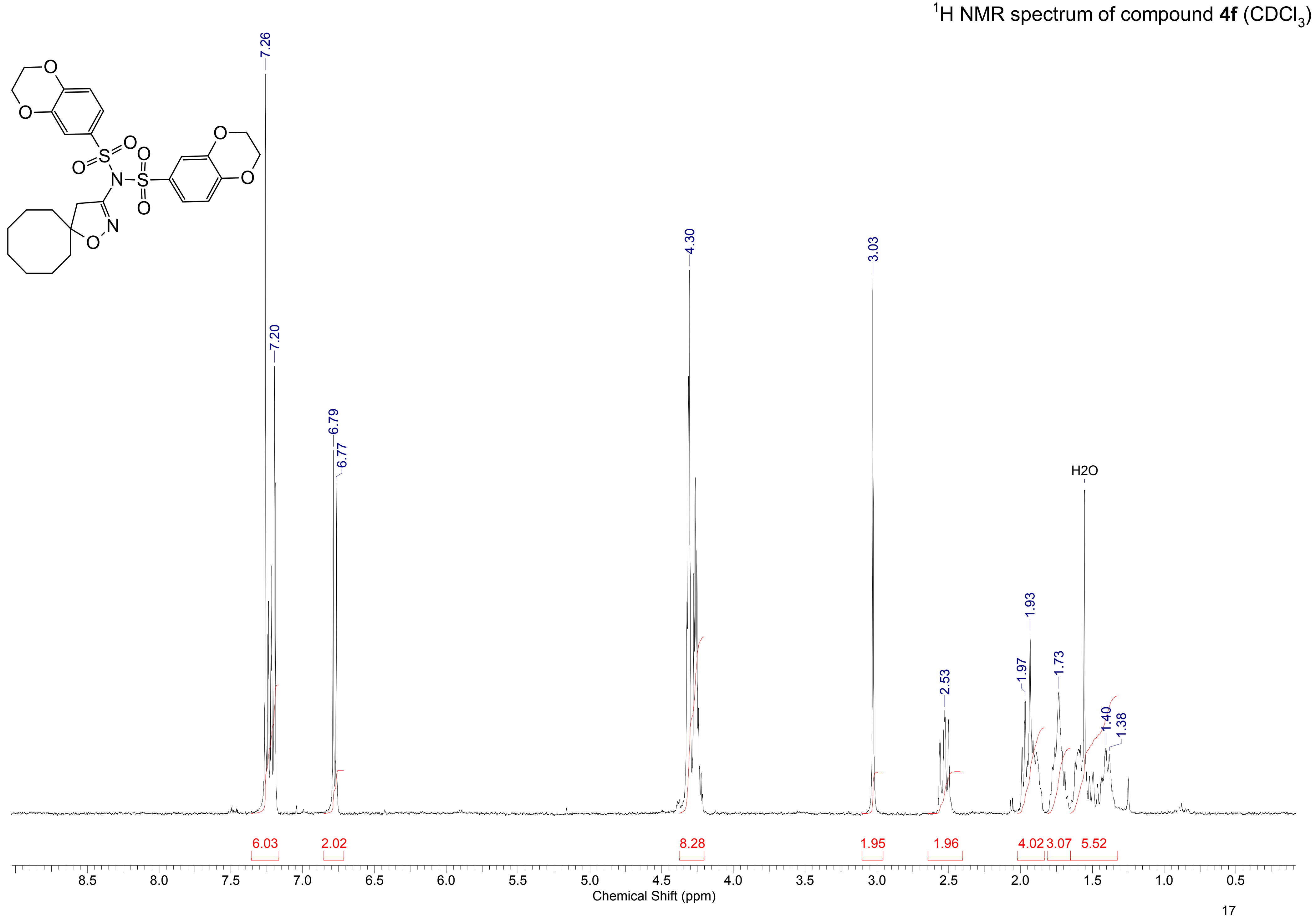

¹H NMR spectrum of compound 4f (CDCl3)
7.26
7.20
6.79
6.77
4.30
3.03
2.53
1.97
1.93
1.73
H2O
1.40
1.38
6.03
2.02
8.28
1.95
1.96
4.02
3.07
5.52
8.5
8.0
7.5
7.0
6.5
6.0
5.5
5.0
4.5
4.0
3.5
3.0
2.5
2.0
1.5
1.0
0.5
Chemical Shift (ppm)

^{13}C NMR spectrum of compound **4f** ($CDCl_3$)

CARBON_02.esp

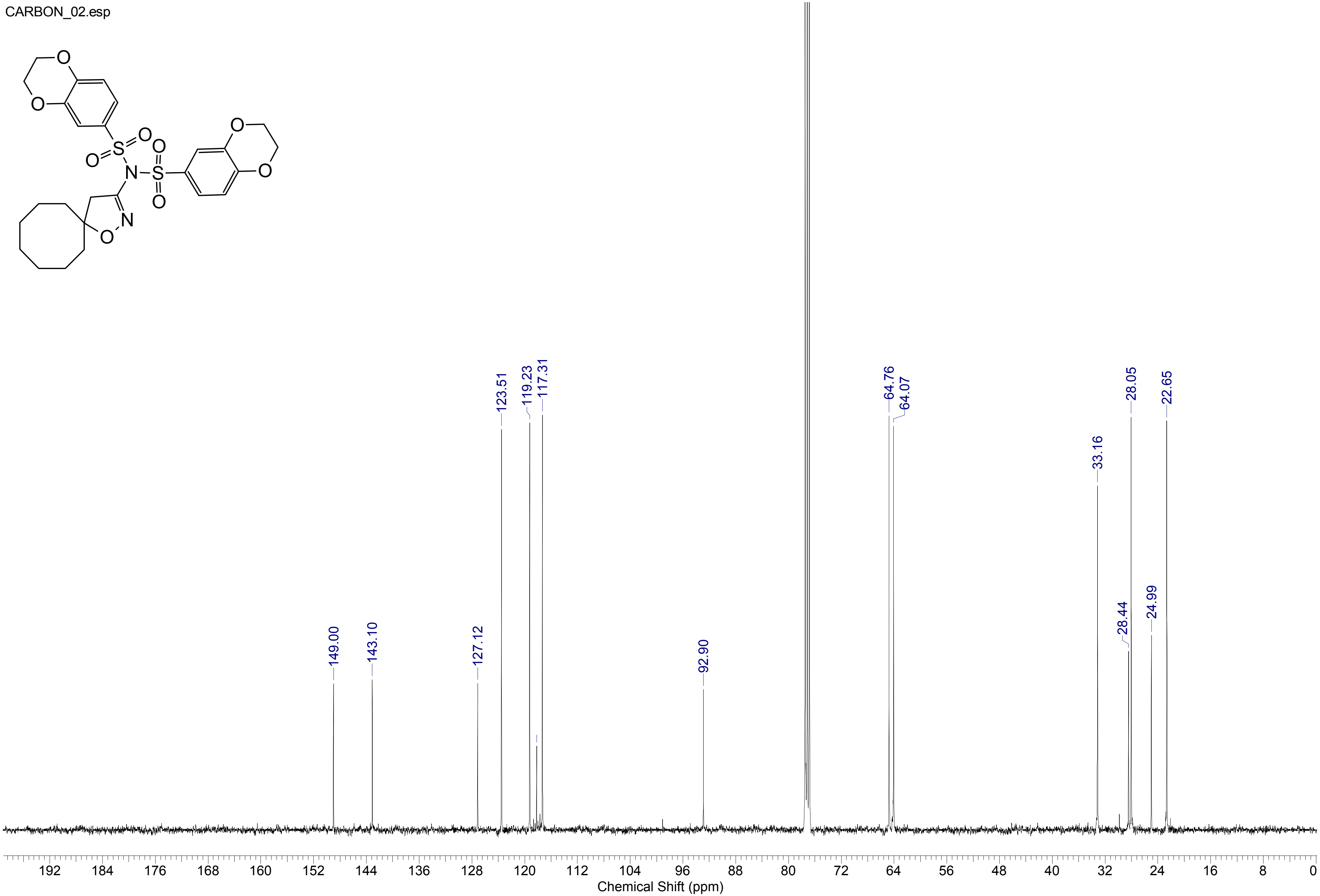

HSQC NMR spectrum of compound **4f** ($CDCl_3$)

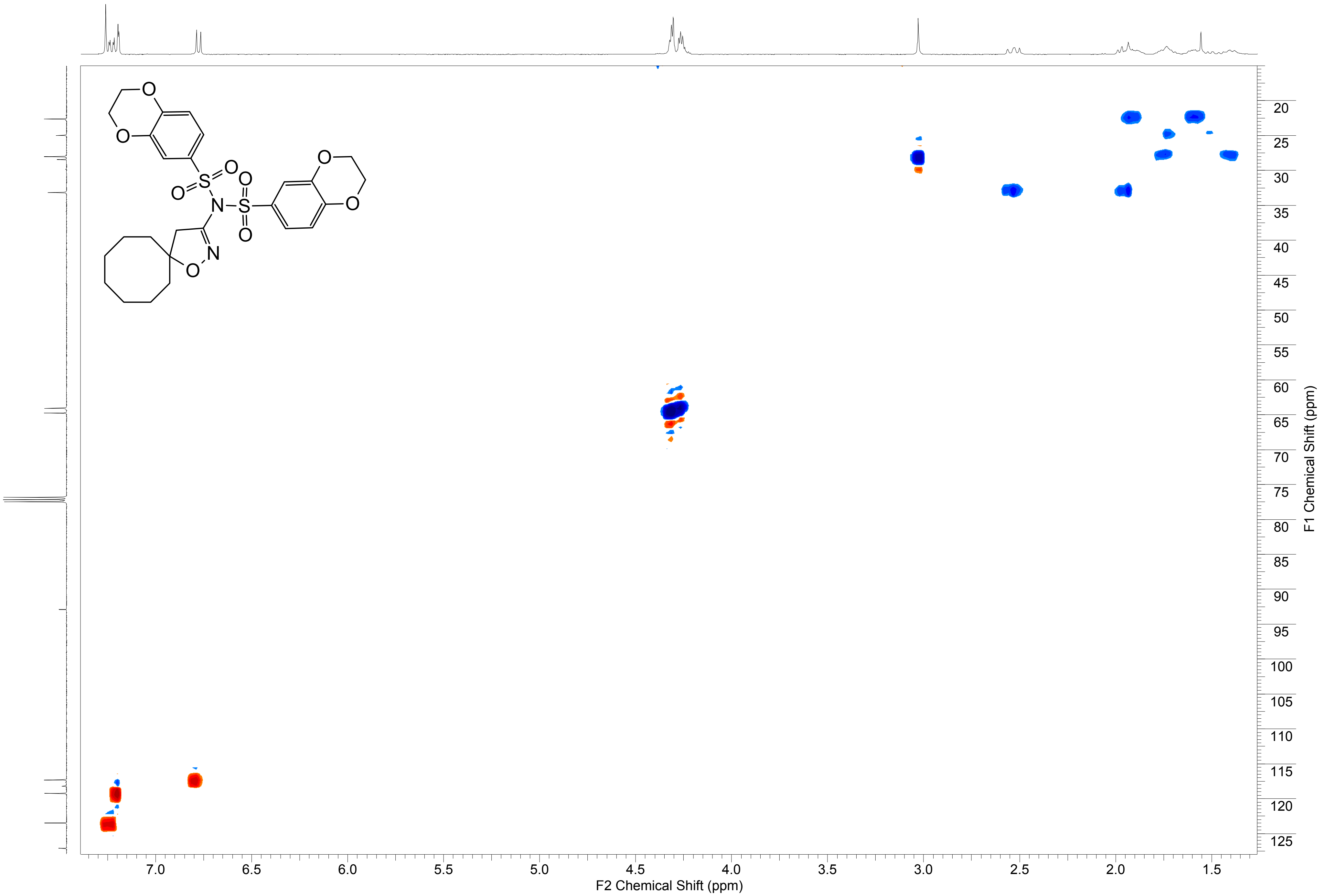

^{1}H NMR spectrum of compound **4g** ($CDCl_3$)

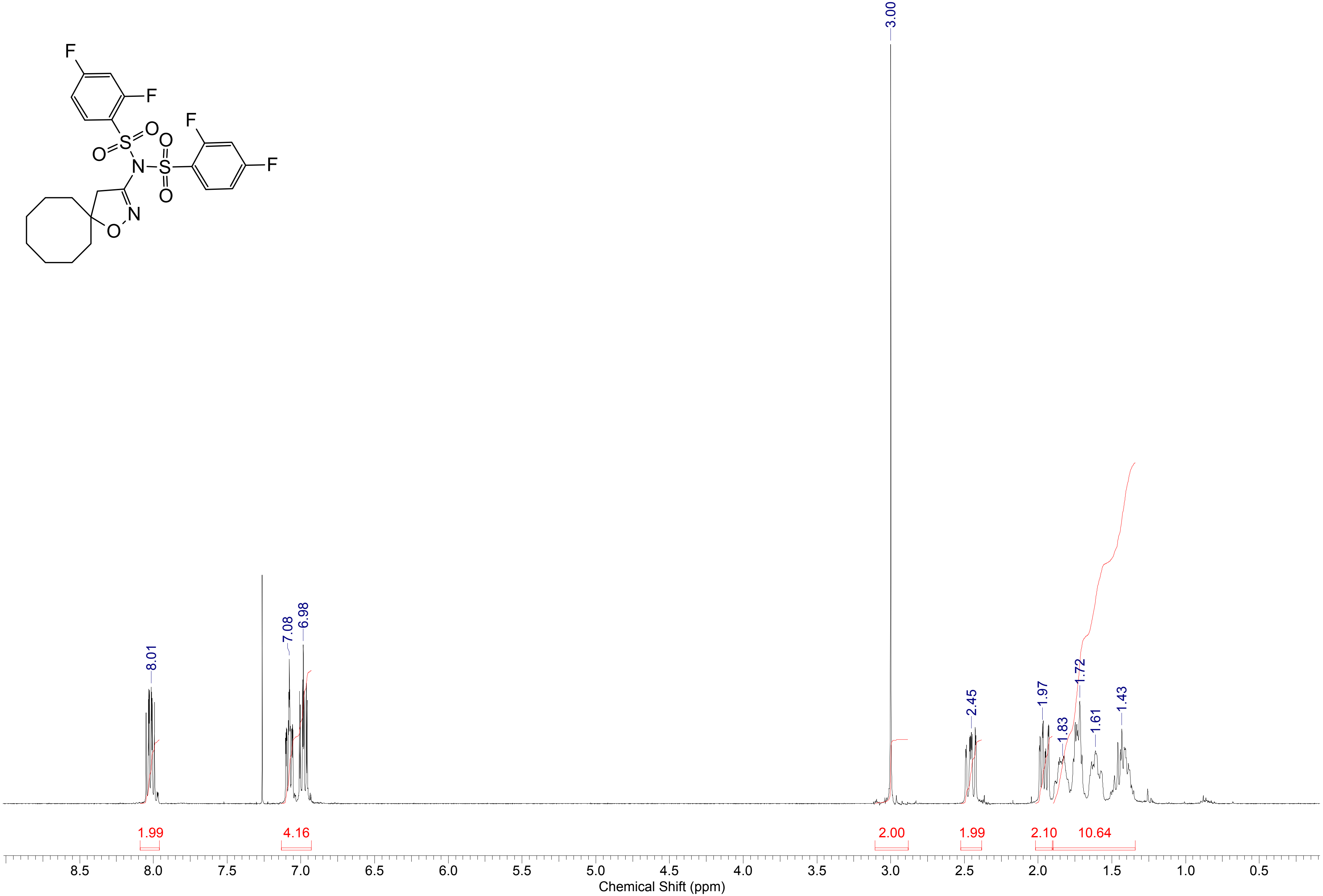

^{13}C NMR spectrum of compound **4g** ($CDCl_3$)

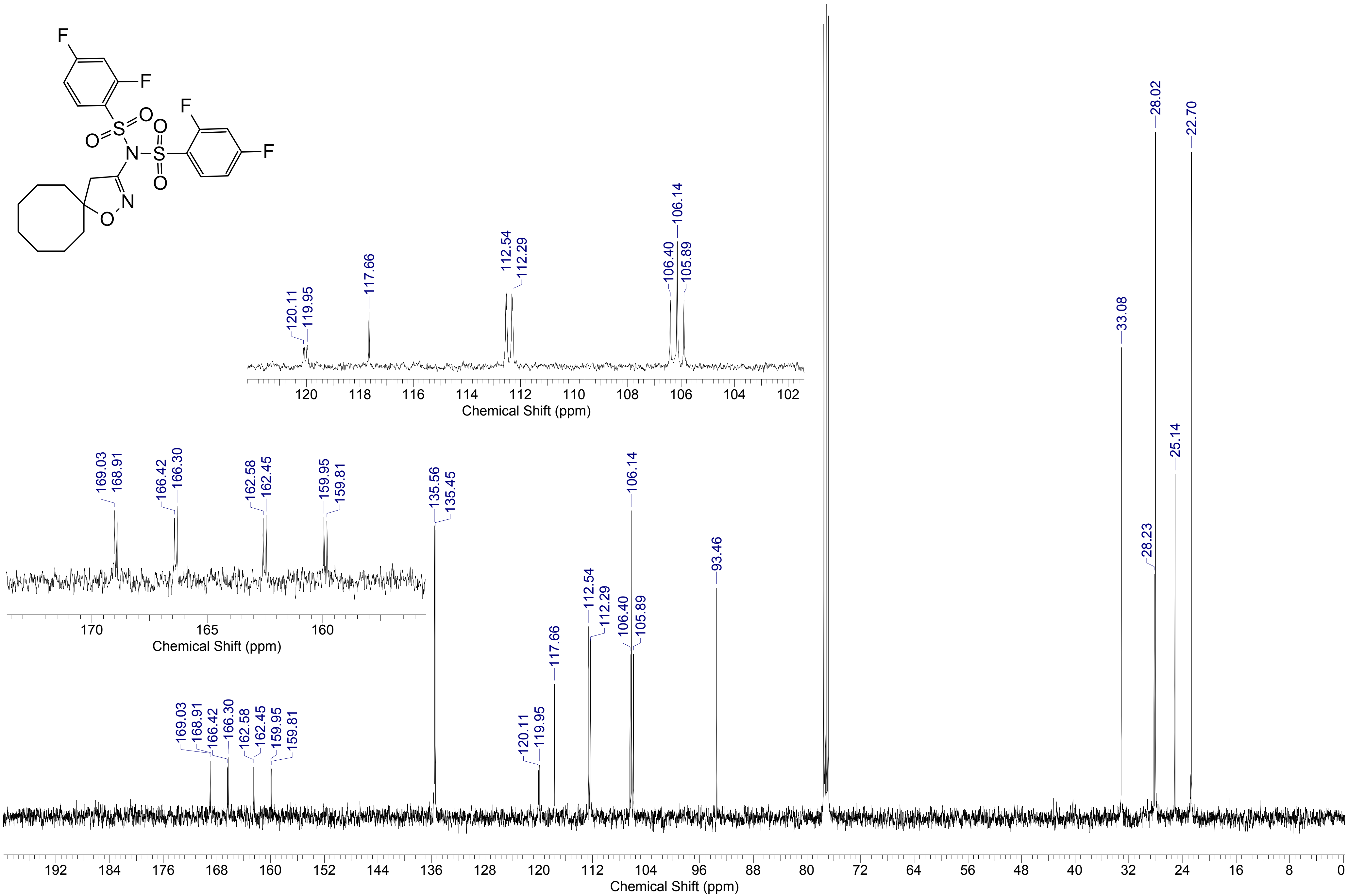

^{19}F NMR spectrum of compound **4g** ($CDCl_3$)

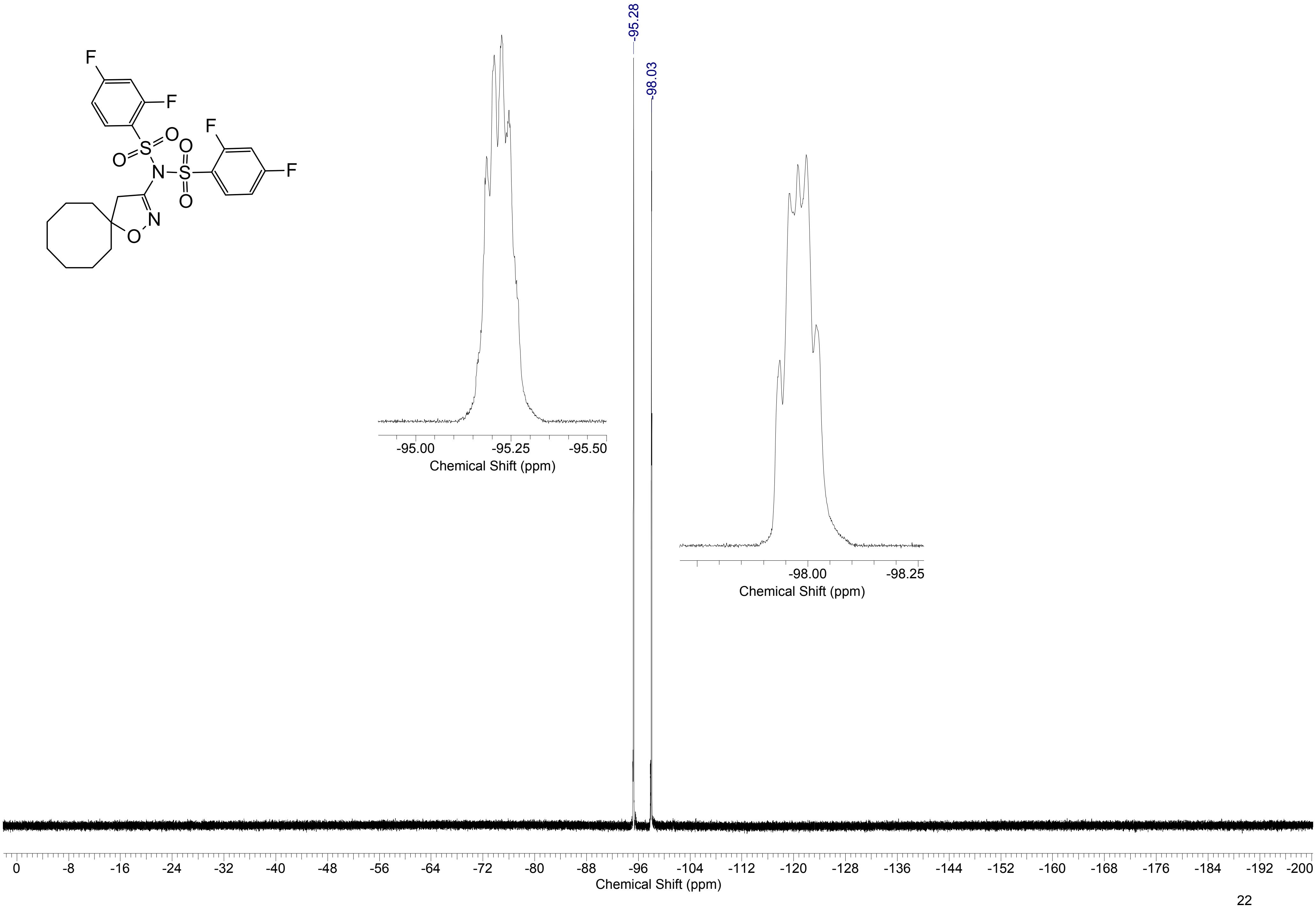

HSQC NMR spectrum of compound **4g** ($CDCl_3$)

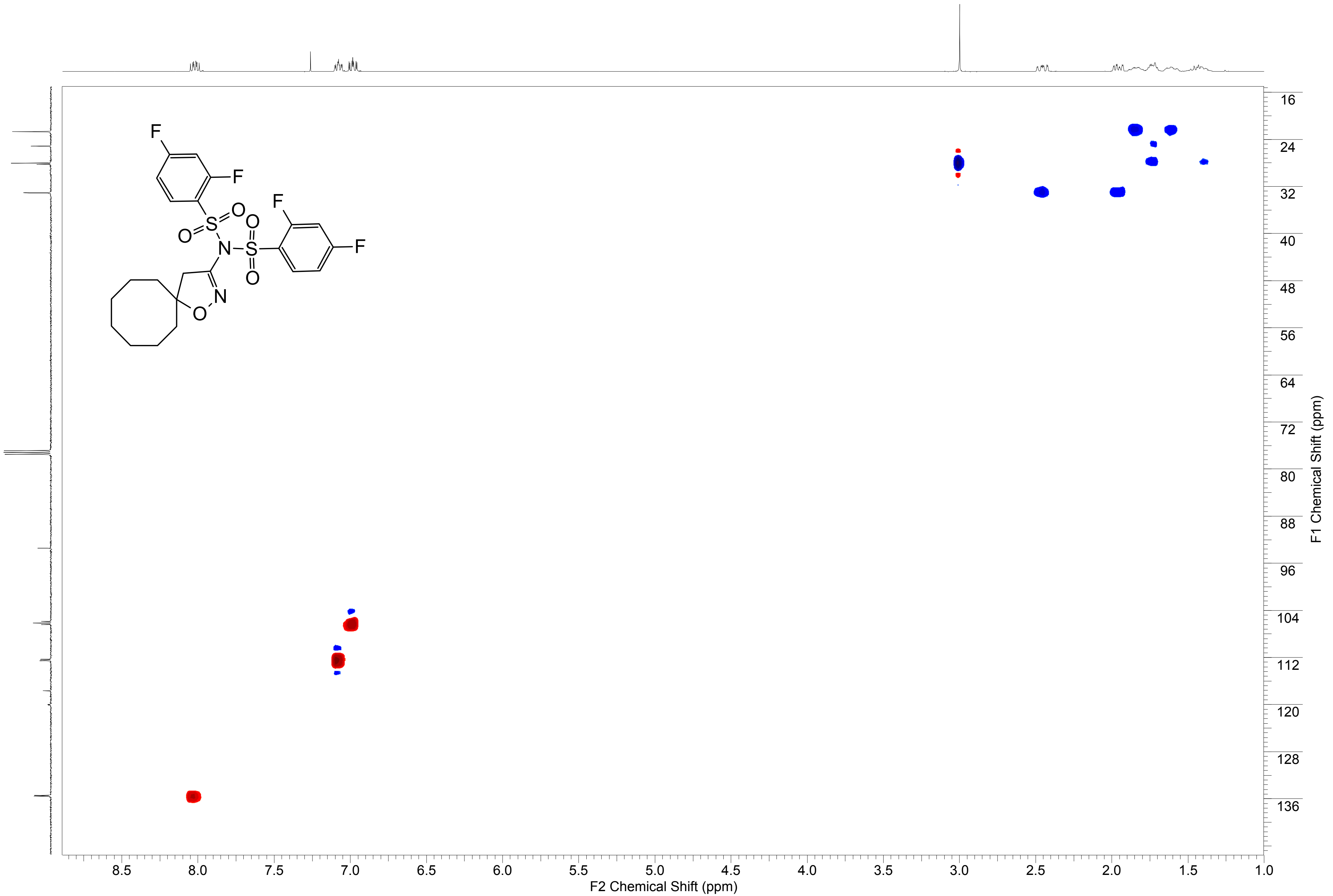

HMBC NMR spectrum of compound **4g** ($CDCl_3$)

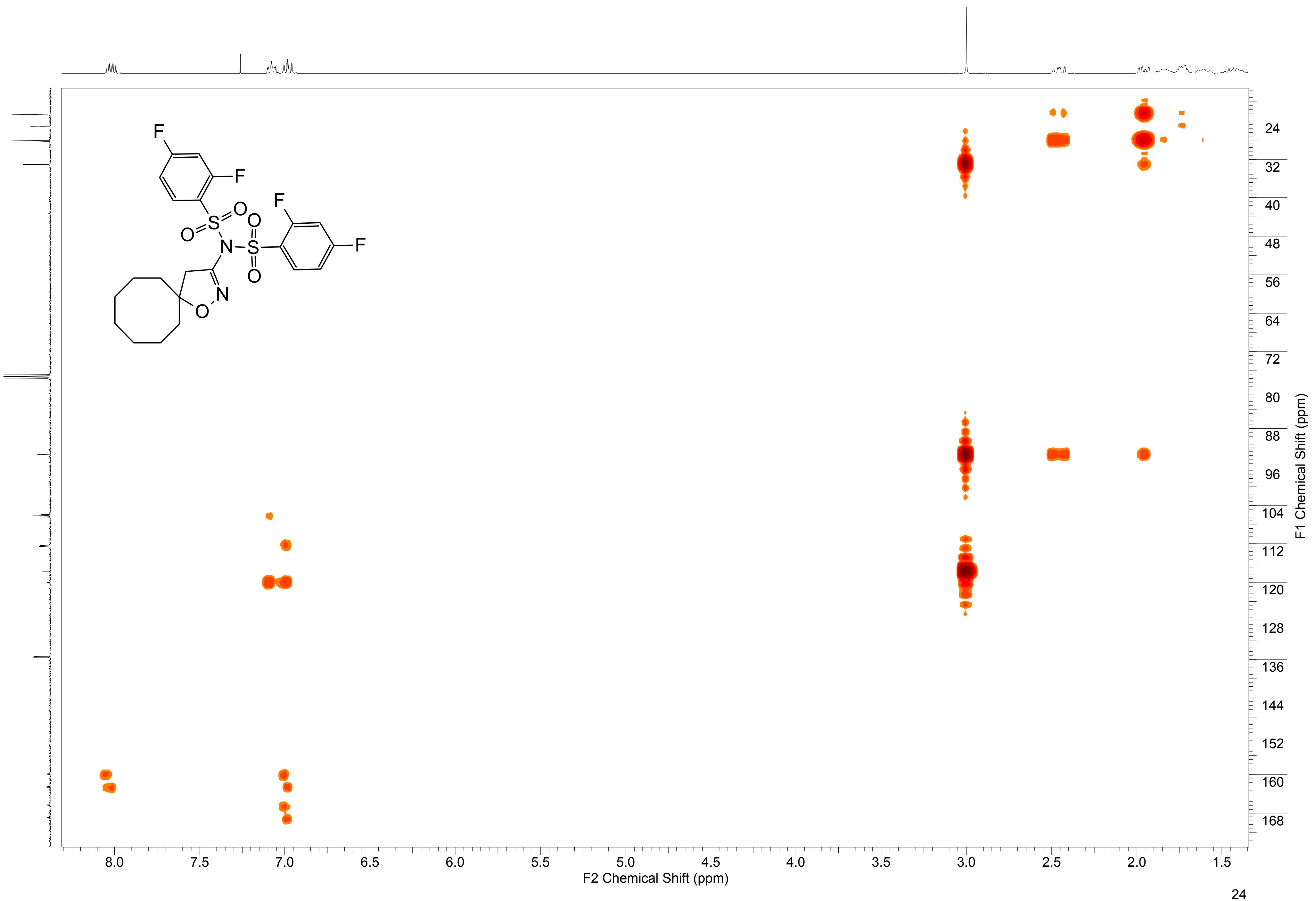

^{1}H NMR spectrum of compound **4h** ($CDCl_3$)

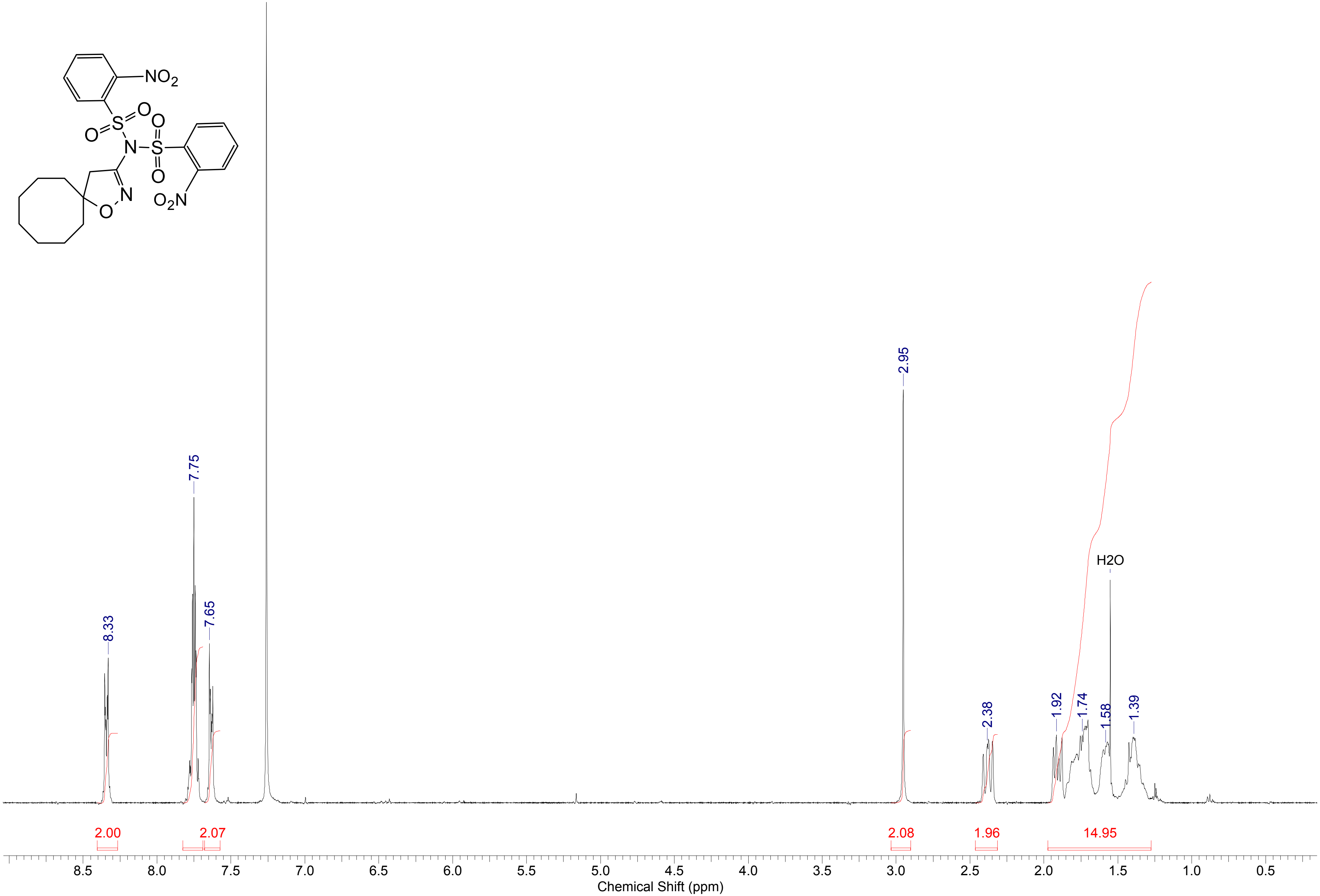

^{13}C NMR spectrum of compound **4h** ($CDCl_3$)

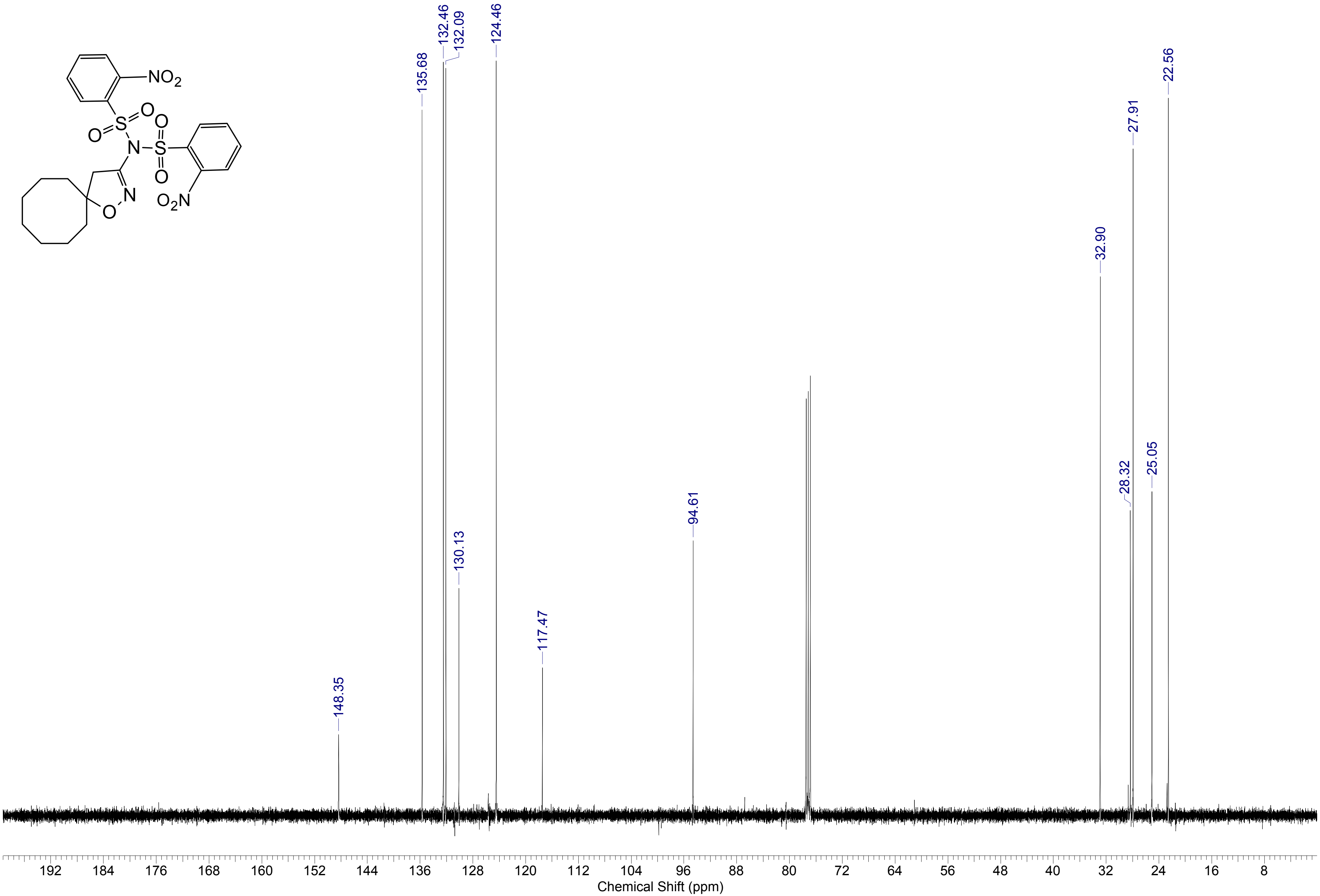

^{1}H NMR spectrum of compound **4i** ($CDCl_3$)

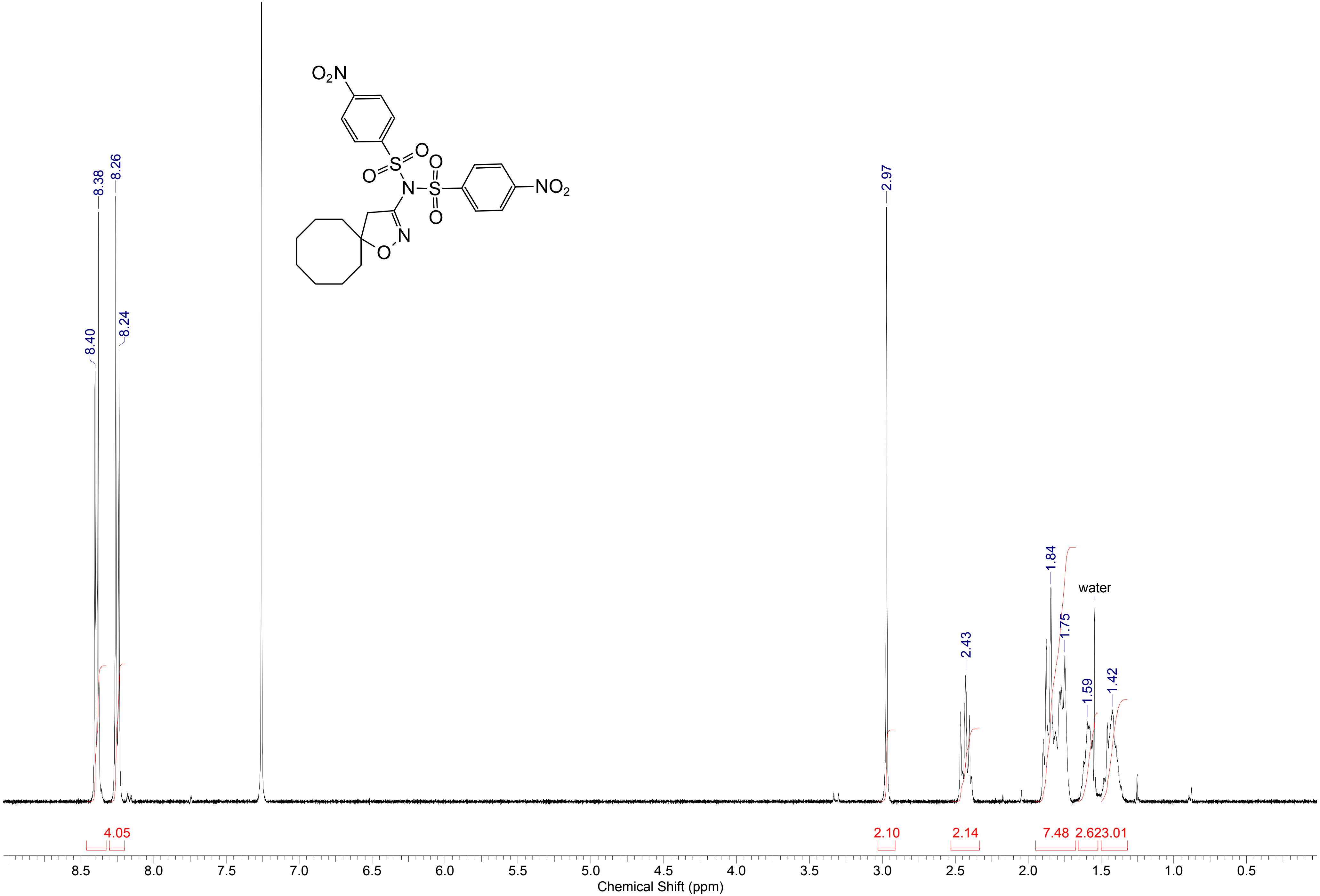

^{13}C NMR spectrum of compound **4i** ($CDCl_3$)

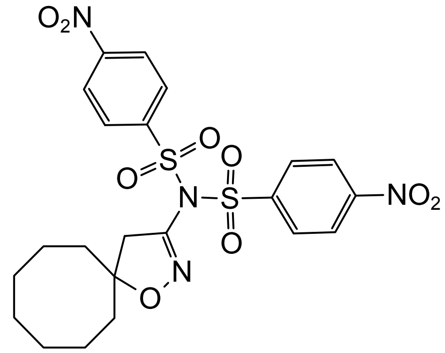

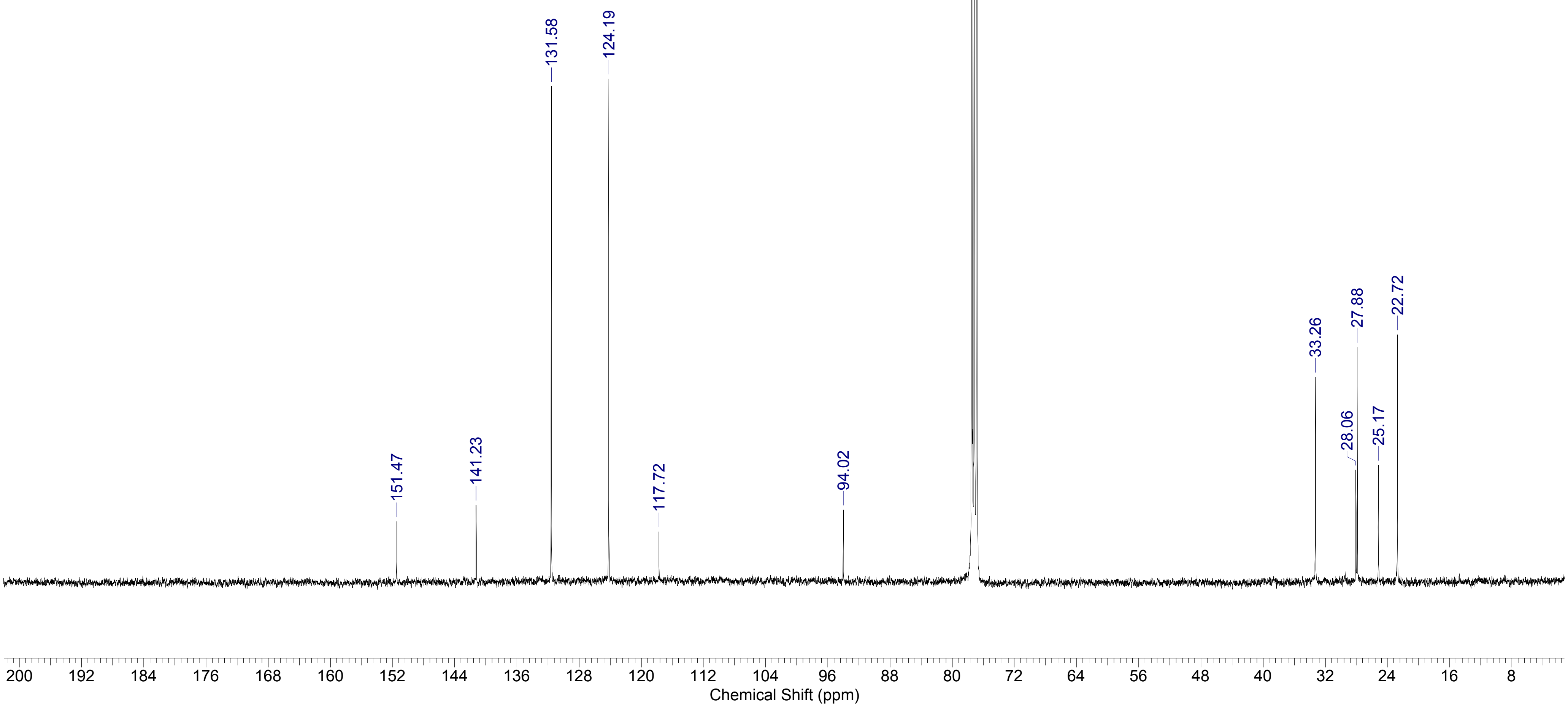

^{1}H NMR spectrum of compound **4j** ($CDCl_3$)

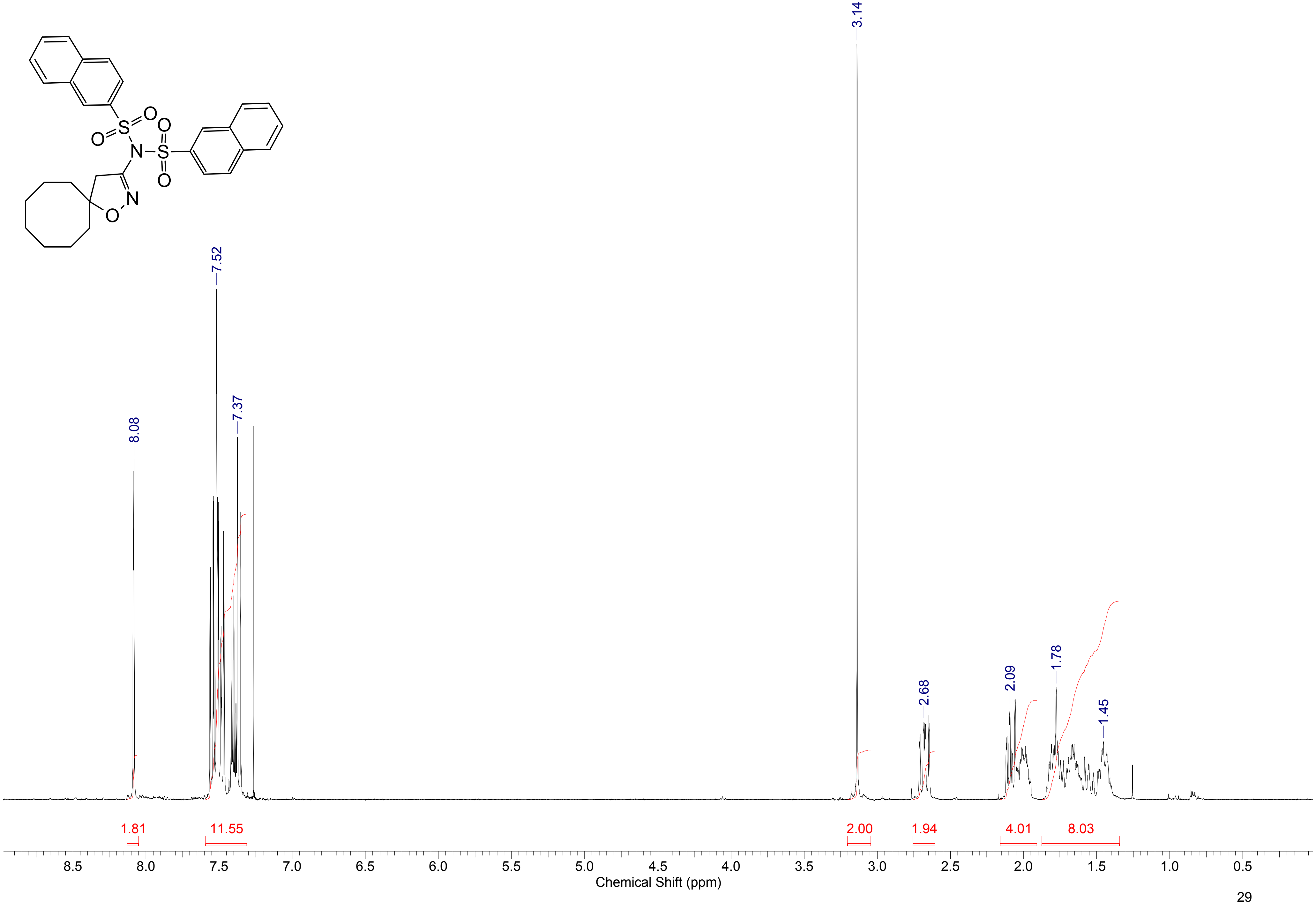

^{13}C NMR spectrum of compound **4j** ($CDCl_3$)

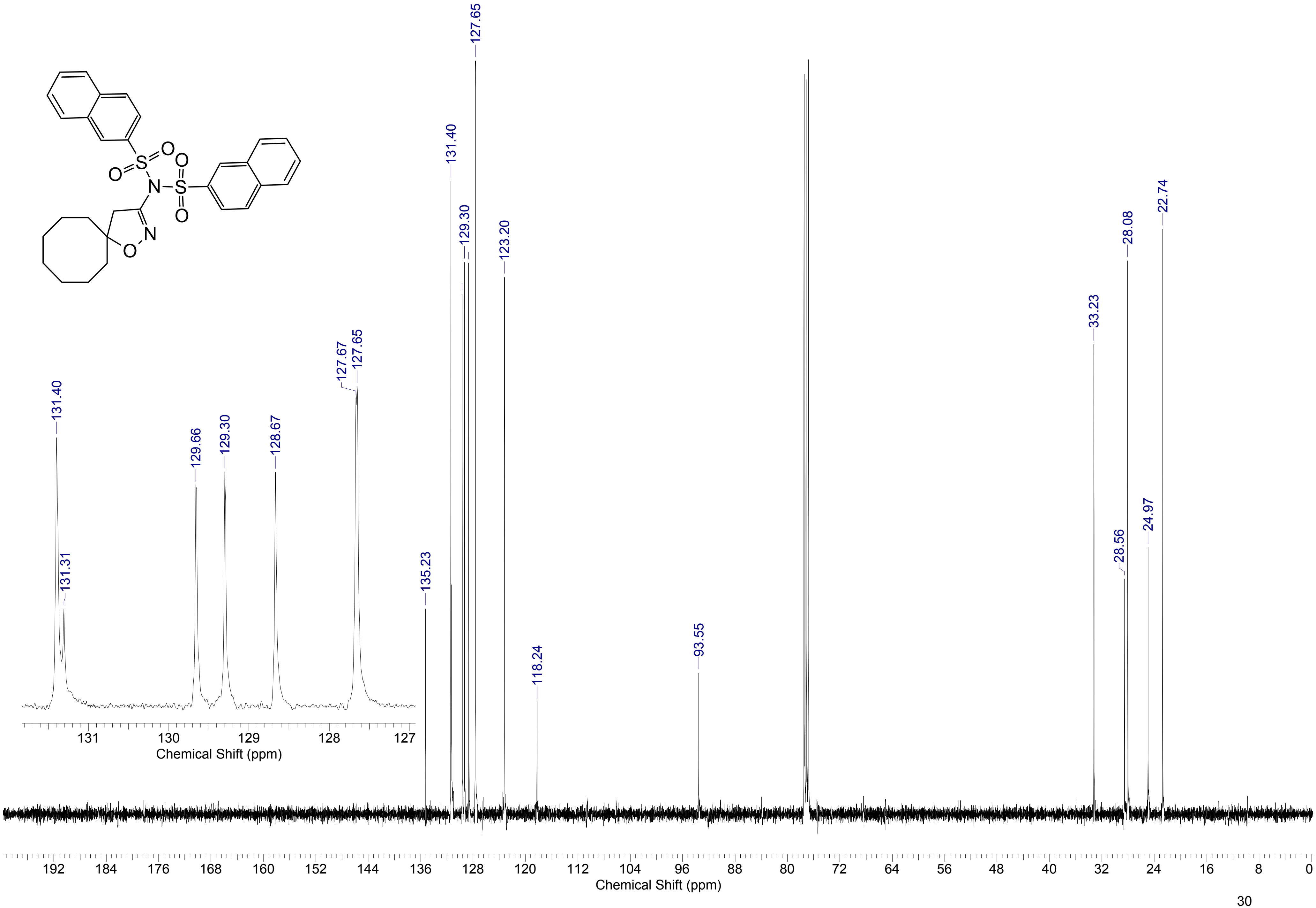

HSQC NMR spectrum of compound **4j** ($CDCl_3$)

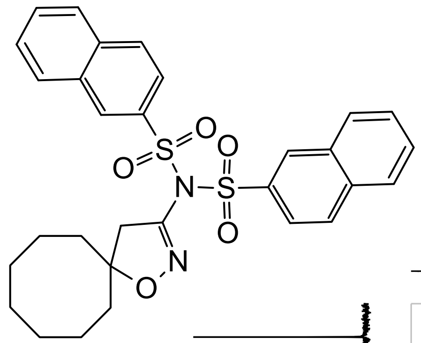

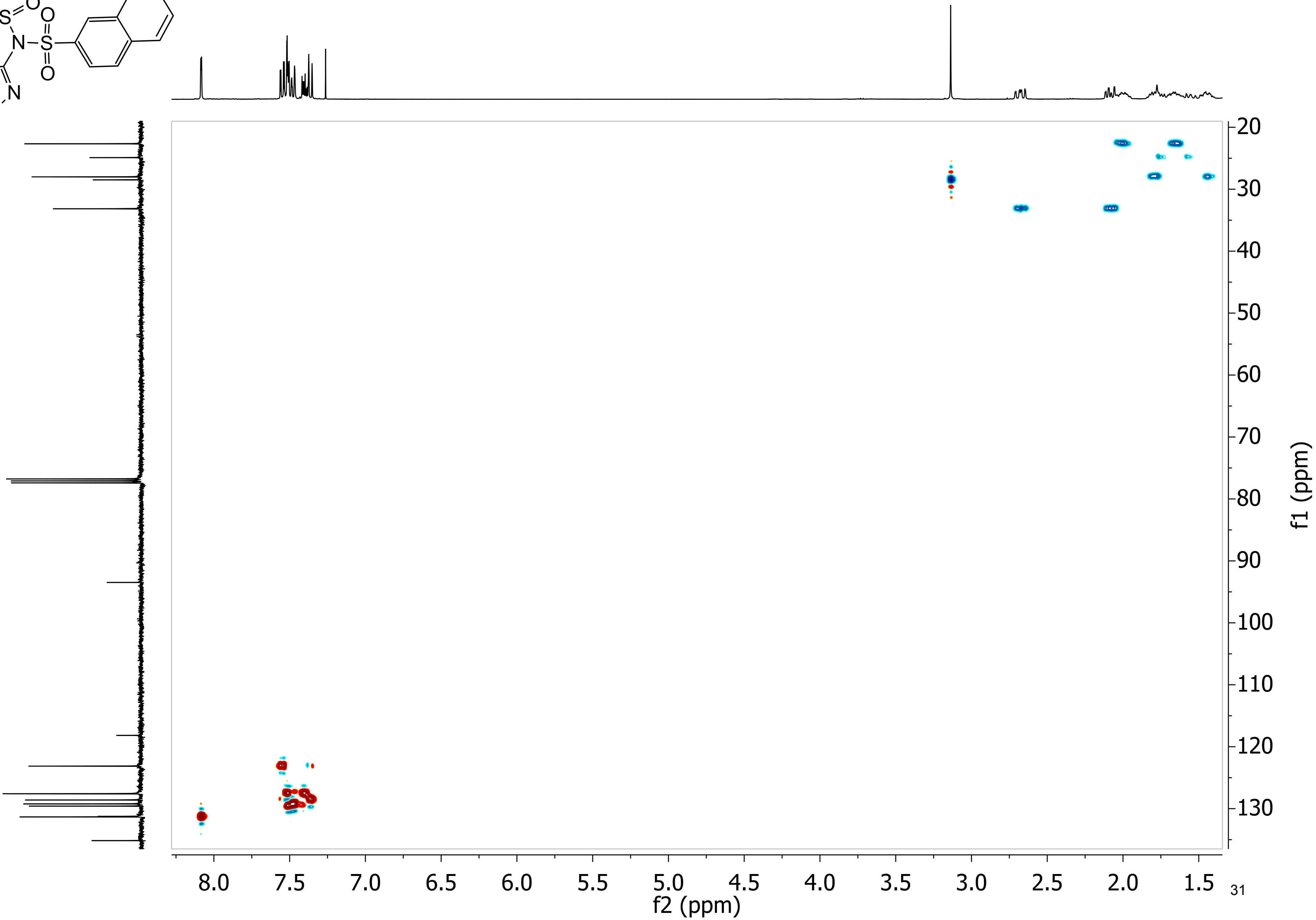

HMBC NMR spectrum of compound **4j** ($CDCl_3$)

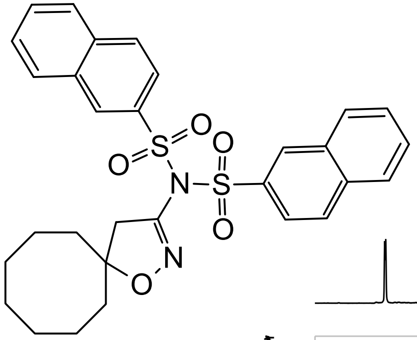

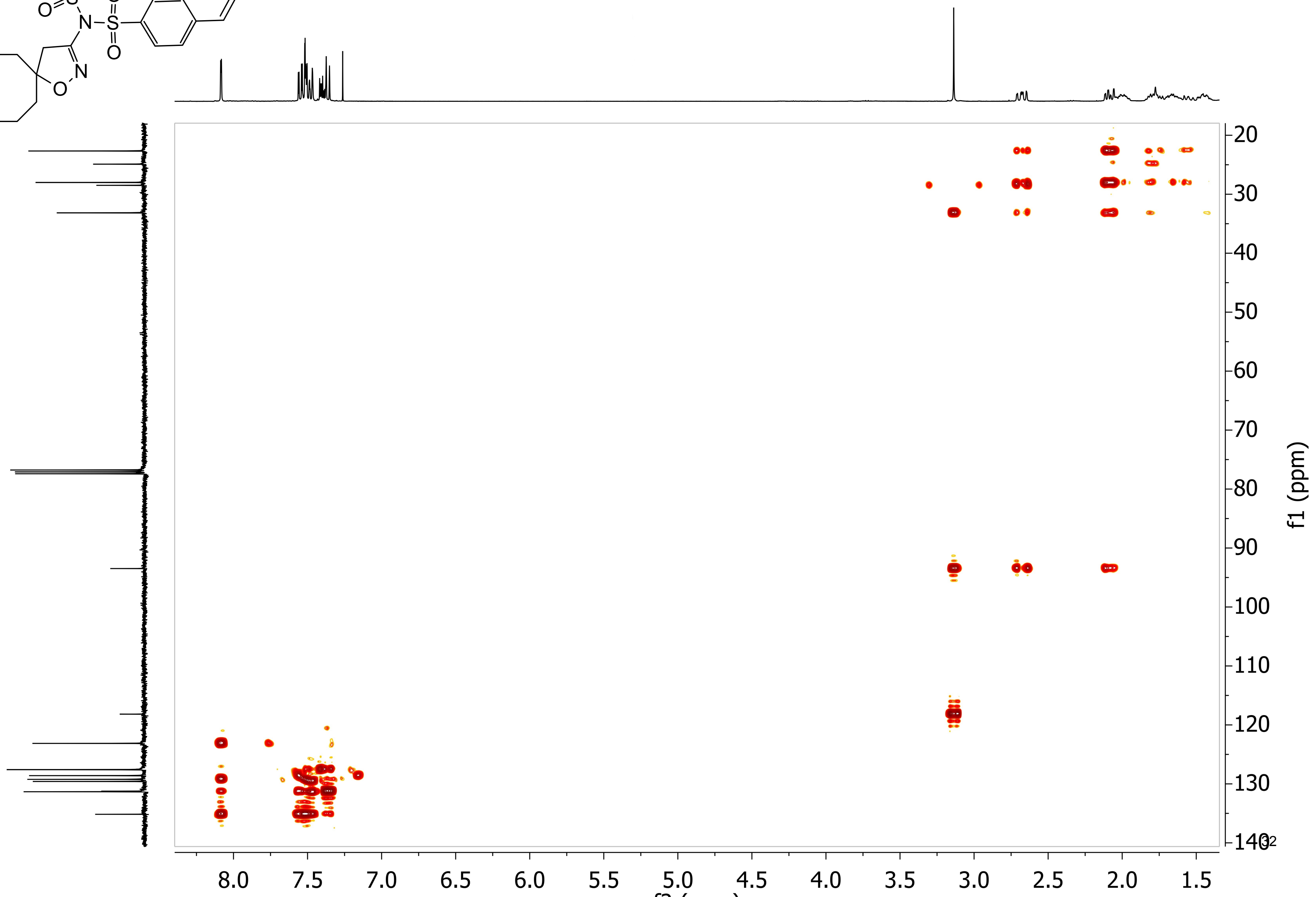

^{1}H NMR spectrum of compound **4k** ($CDCl_3$)

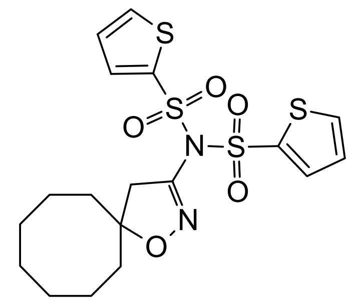

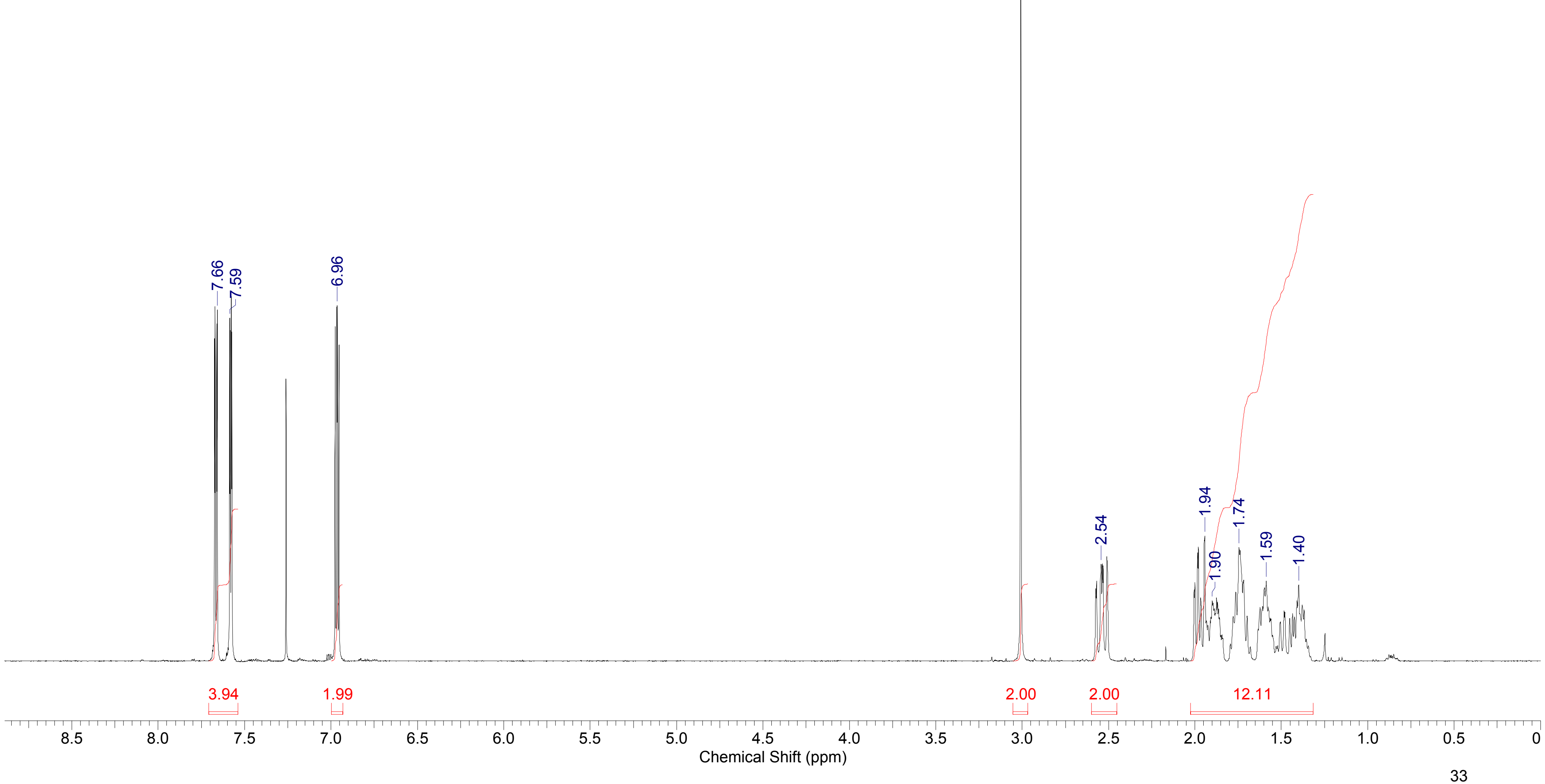

^{13}C NMR spectrum of compound **4k** ($CDCl_3$)

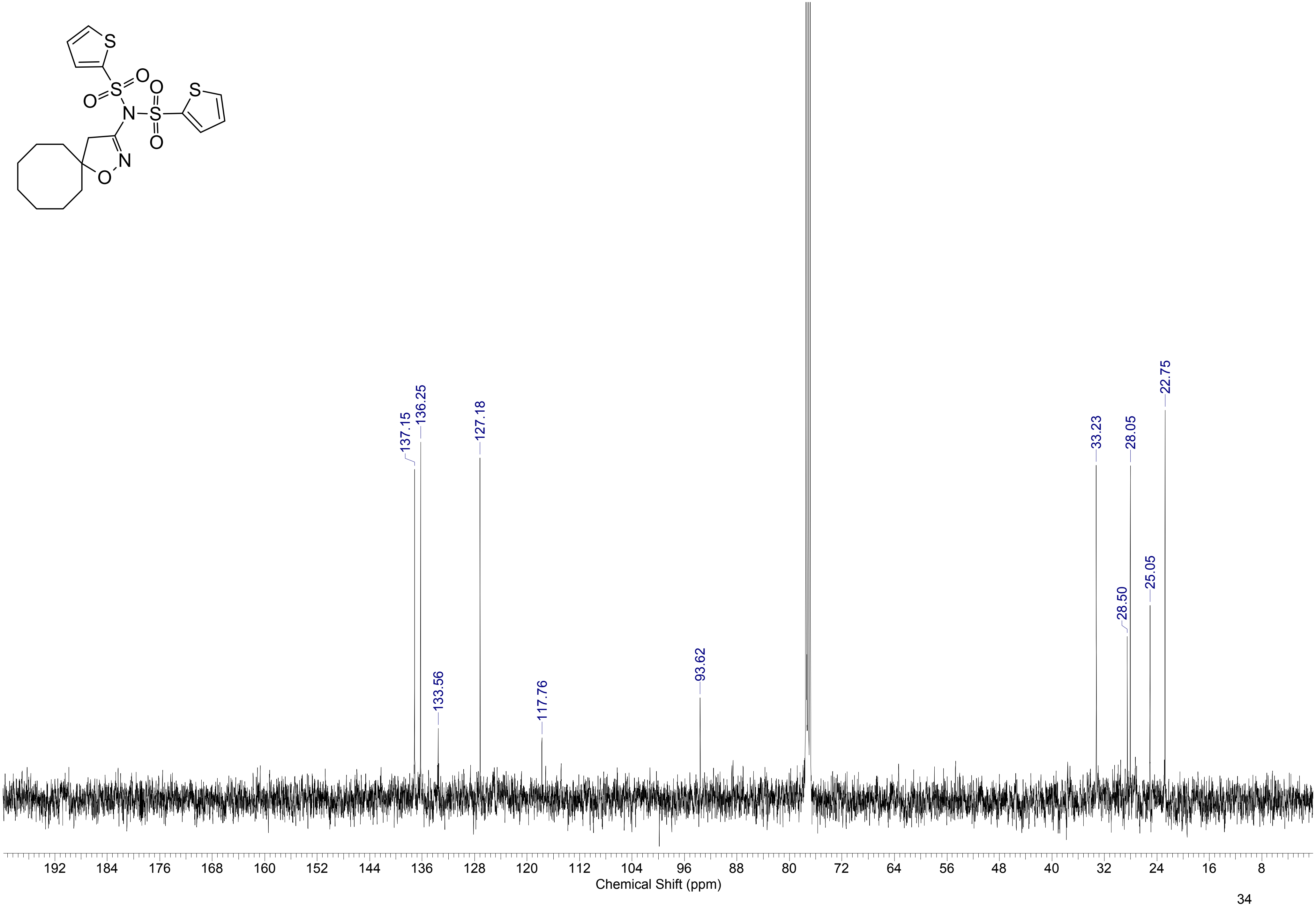

^{1}H NMR spectrum of compound **4l** ($CDCl_3$)

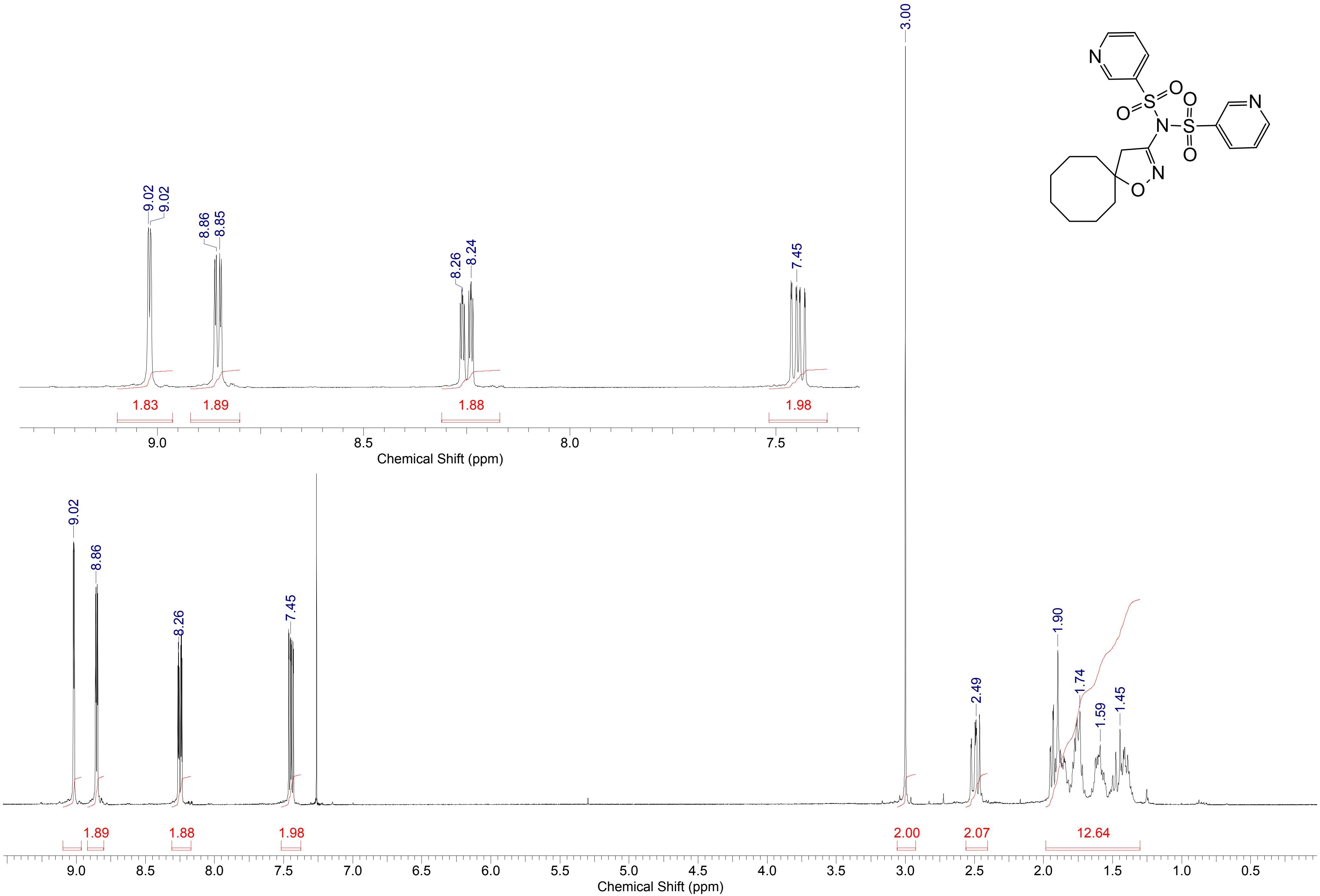

^{13}C NMR spectrum of compound **4l** ($CDCl_3$)

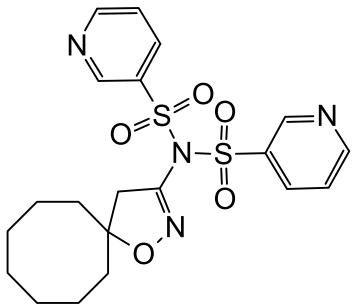

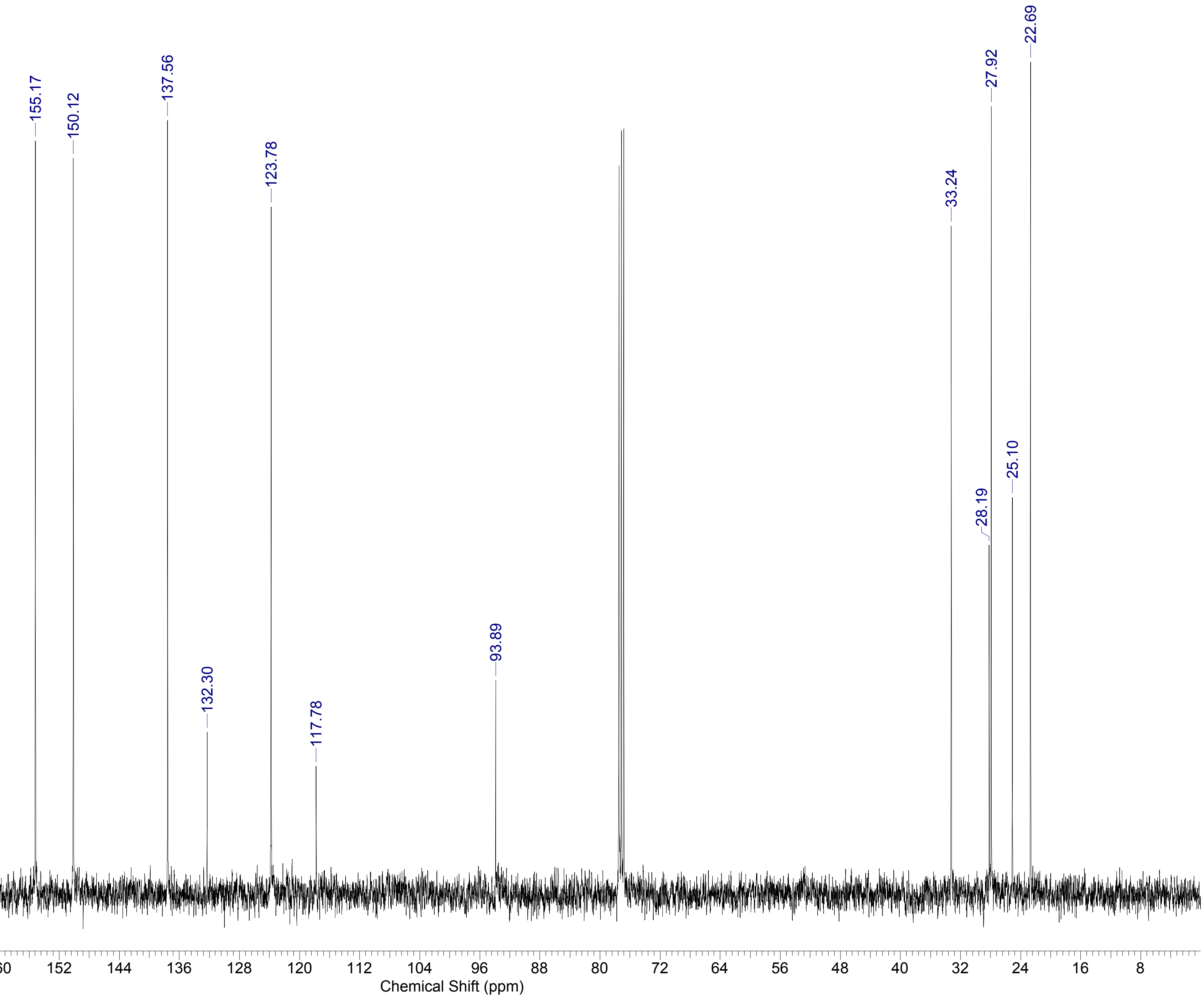

HSQC NMR spectrum of compound **4l** ($CDCl_3$)

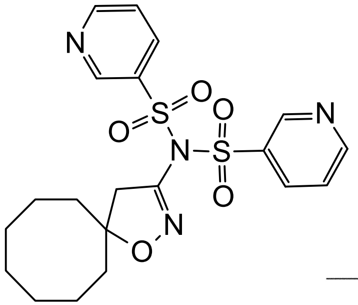

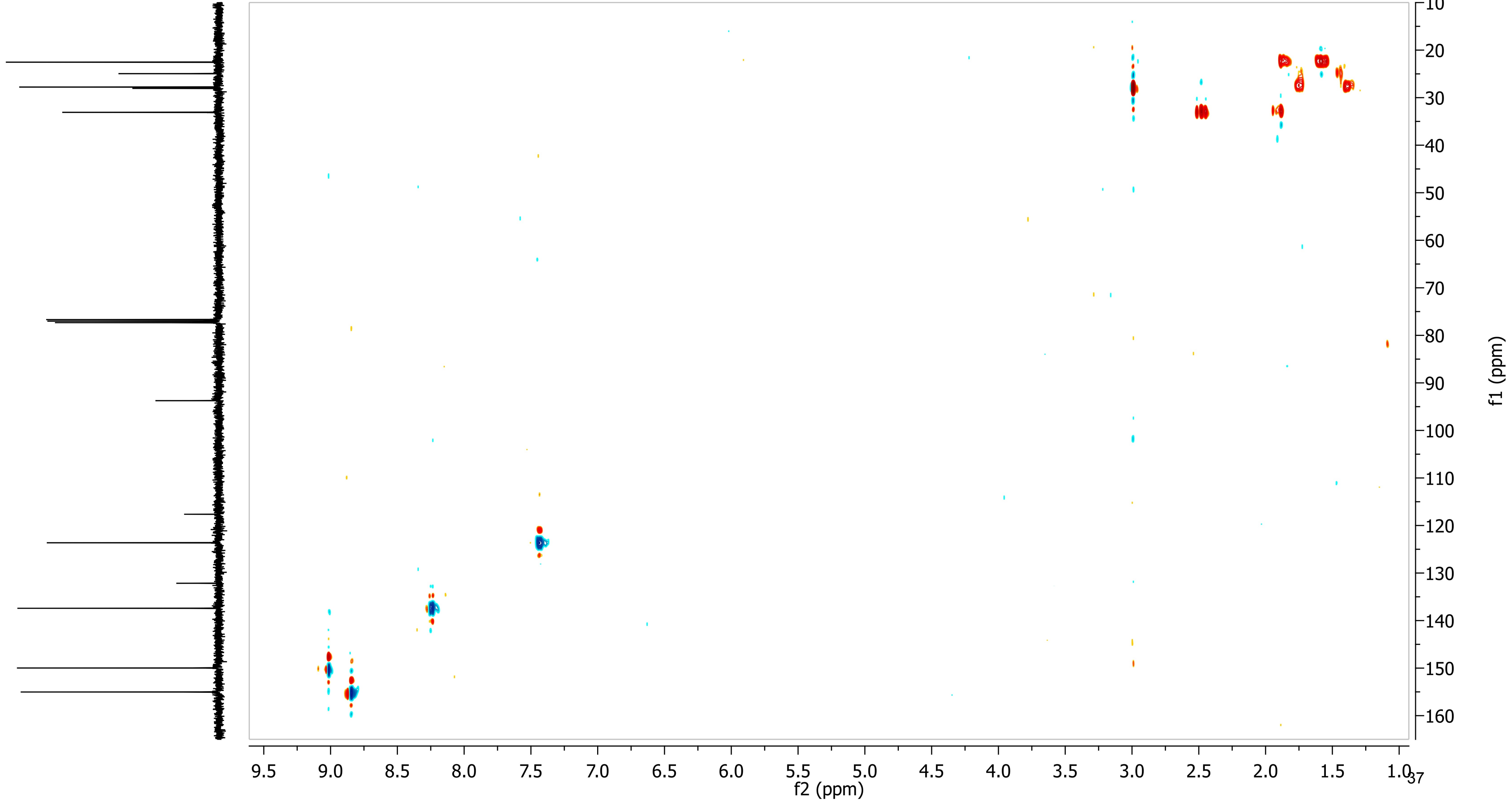

^{1}H NMR spectrum of compound **5b** ($CDCl_3$)

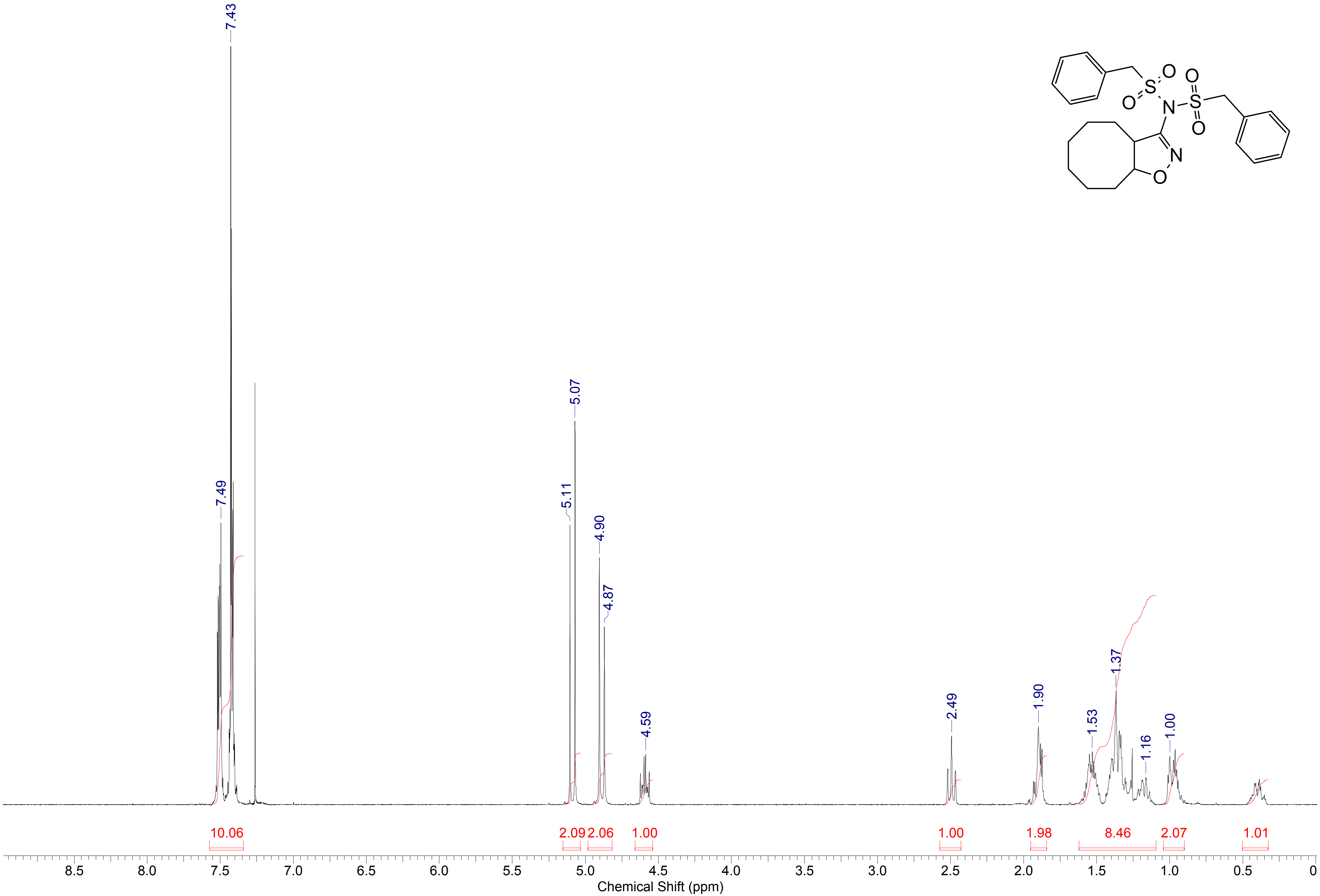

^{13}C NMR spectrum of compound **5b** ($CDCl_3$)

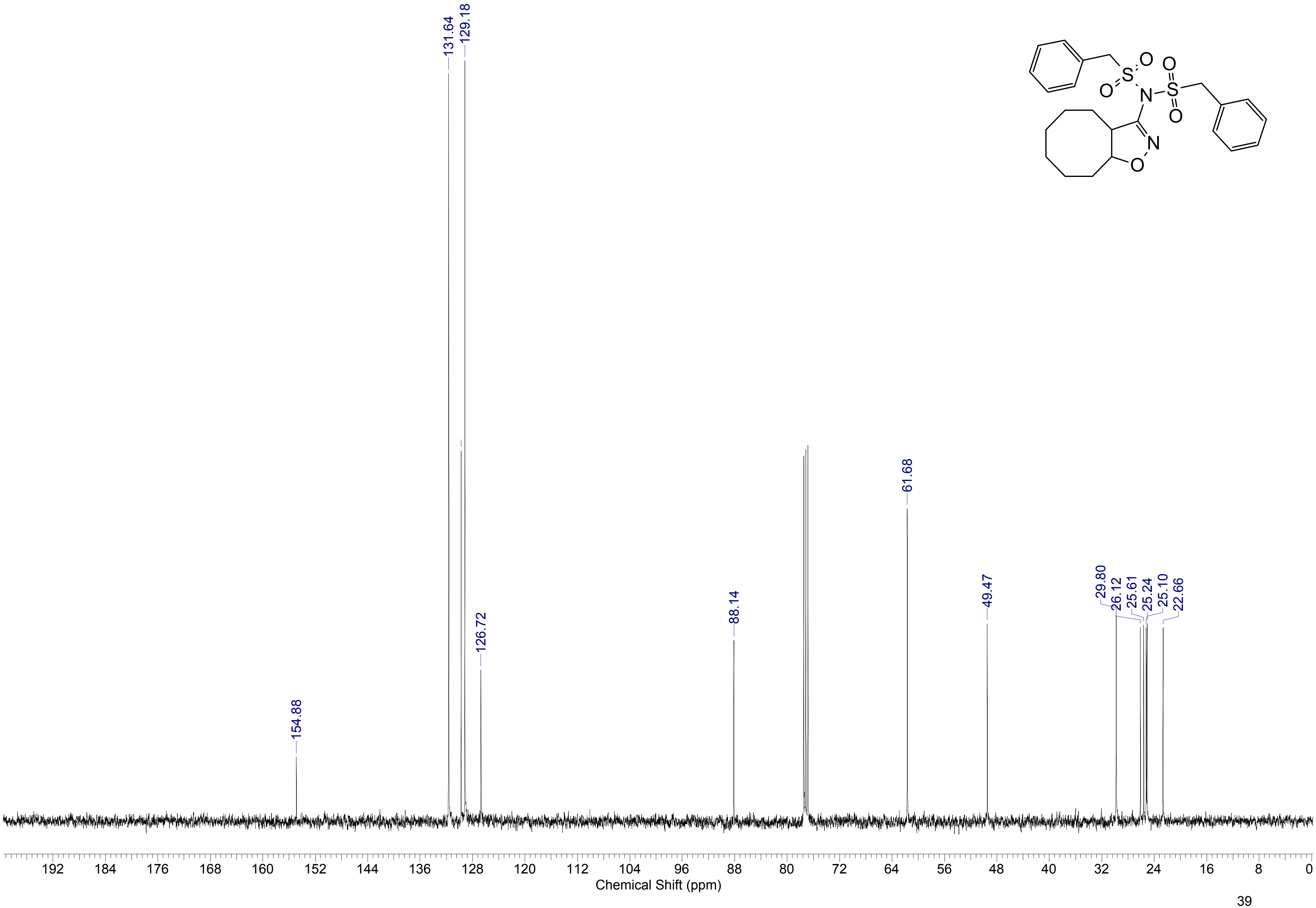

HSQC NMR spectrum of compound **5b** ($CDCl_3$)

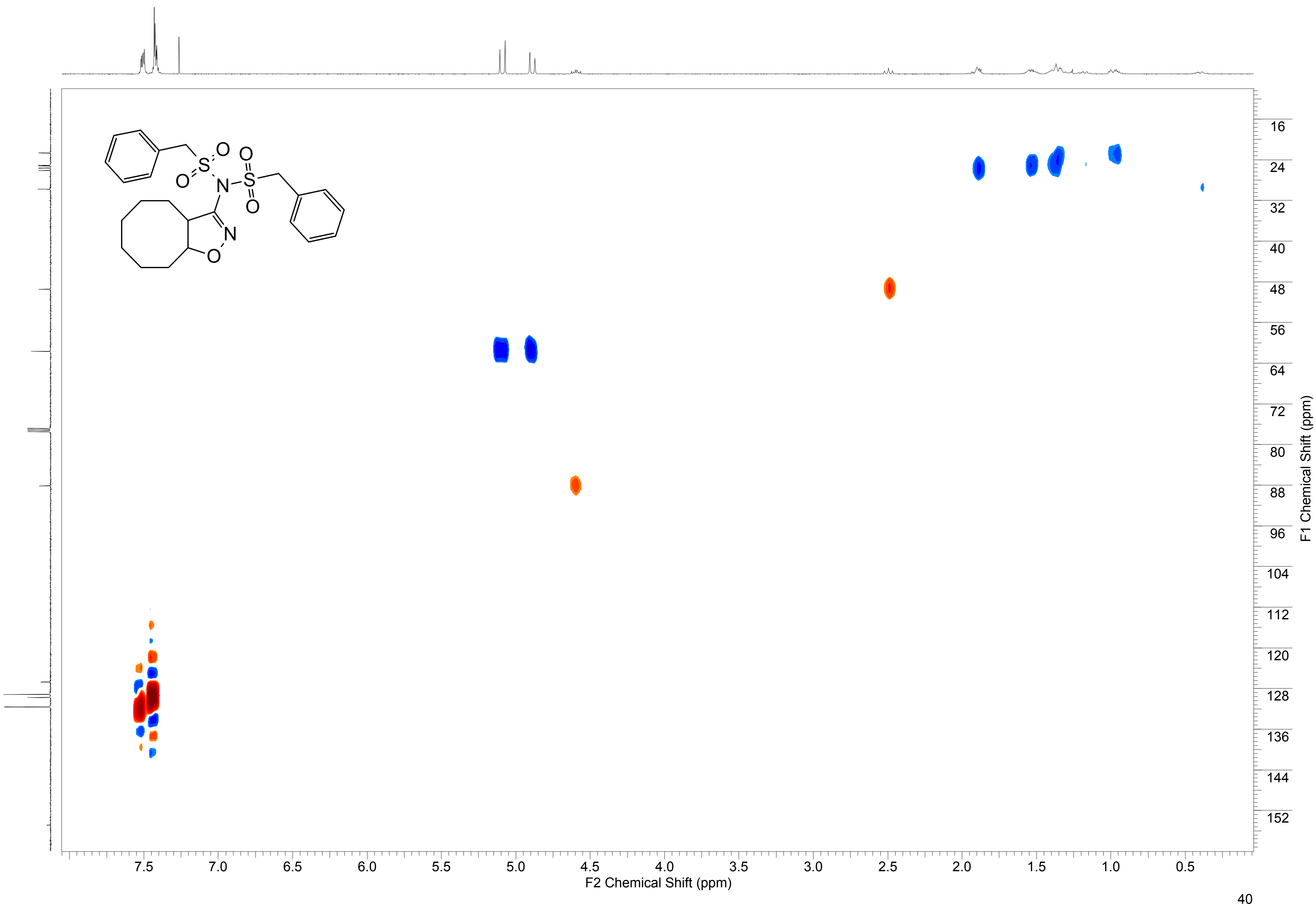

^{1}H NMR spectrum of compound **5c** ($CDCl_3$)

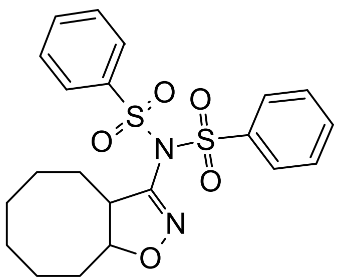

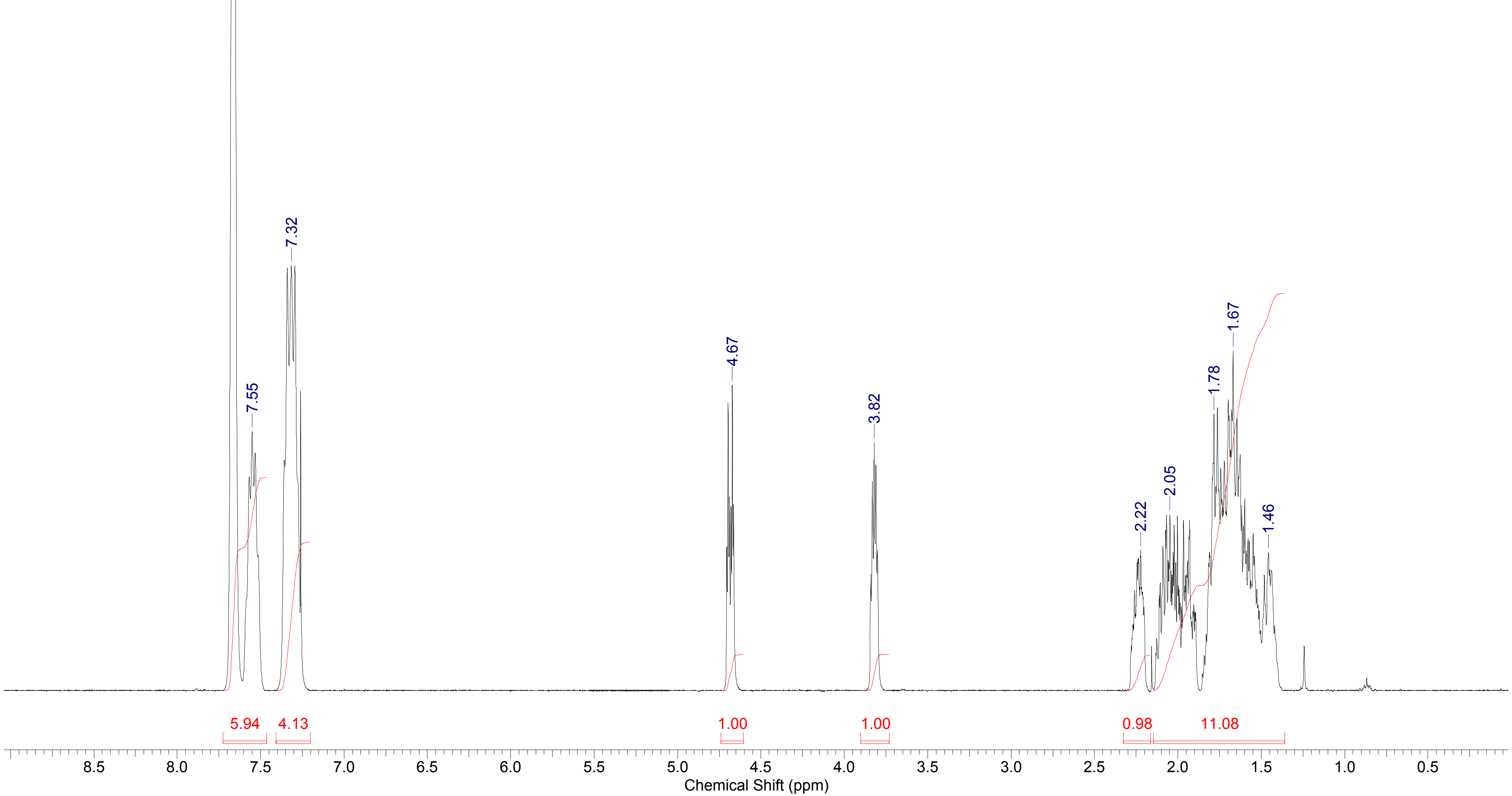

^{13}C NMR spectrum of compound **5c** ($CDCl_3$)

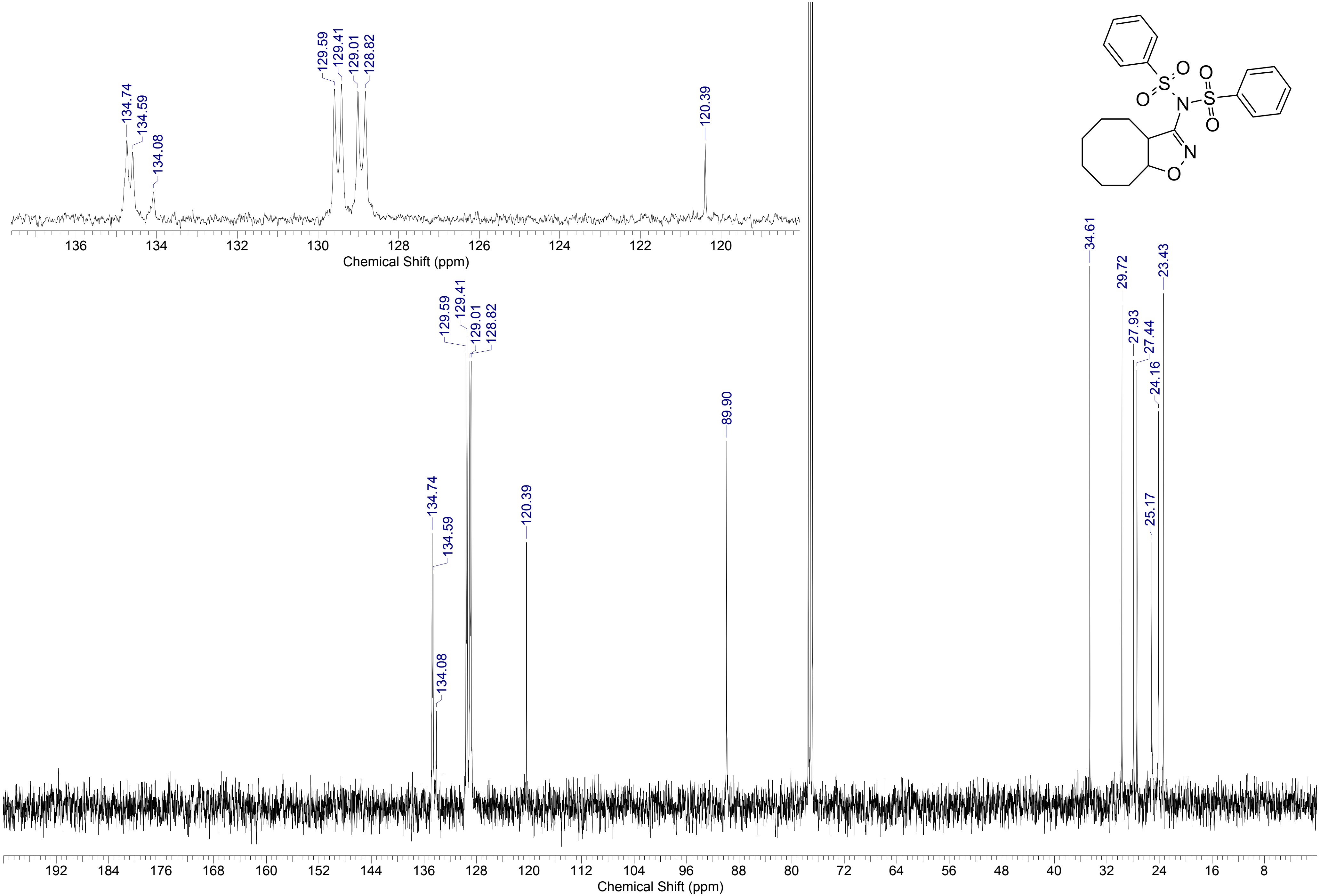

^{1}H NMR spectrum of compound **5d** ($CDCl_3$)

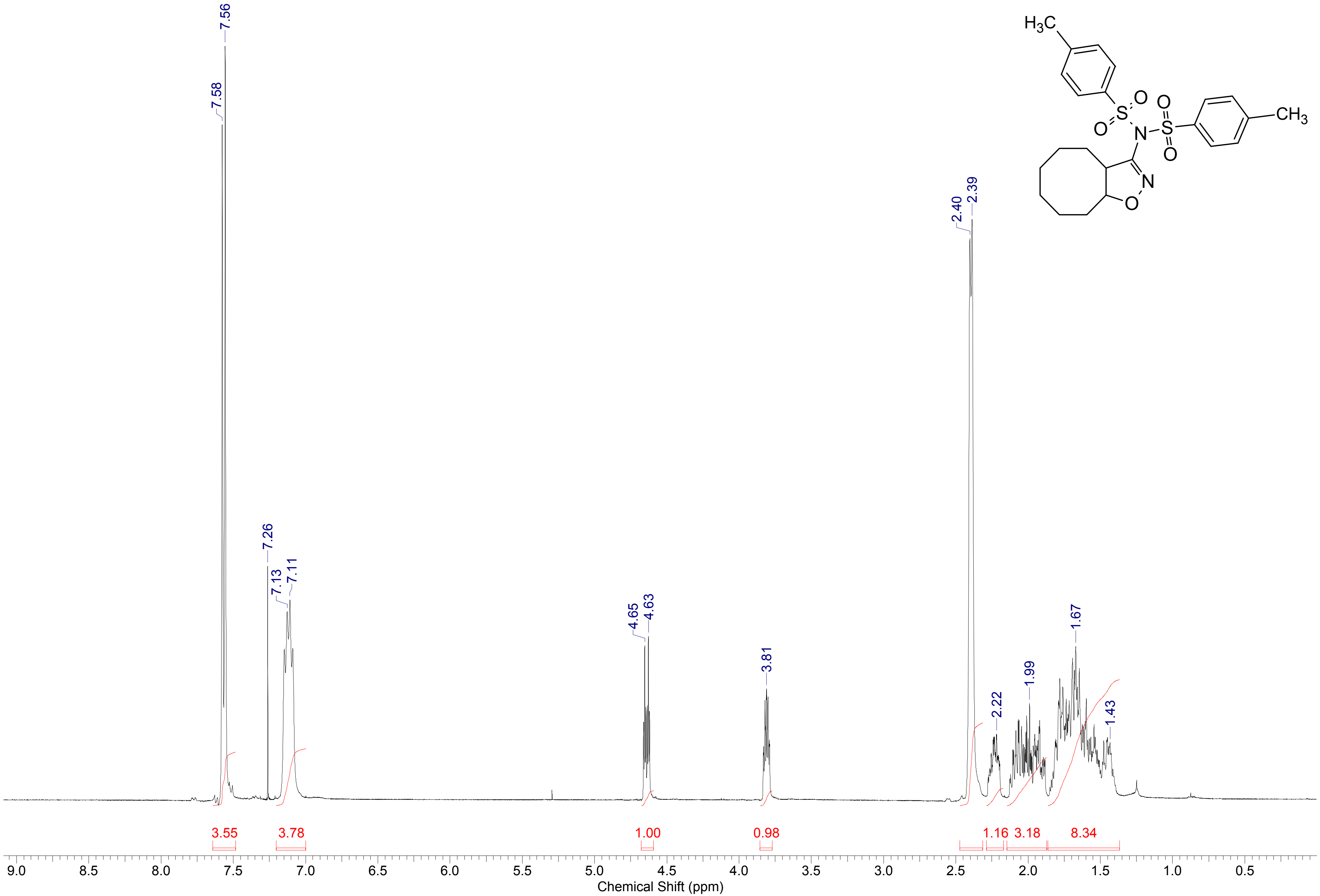

^{13}C NMR spectrum of compound **5d** ($CDCl_3$)

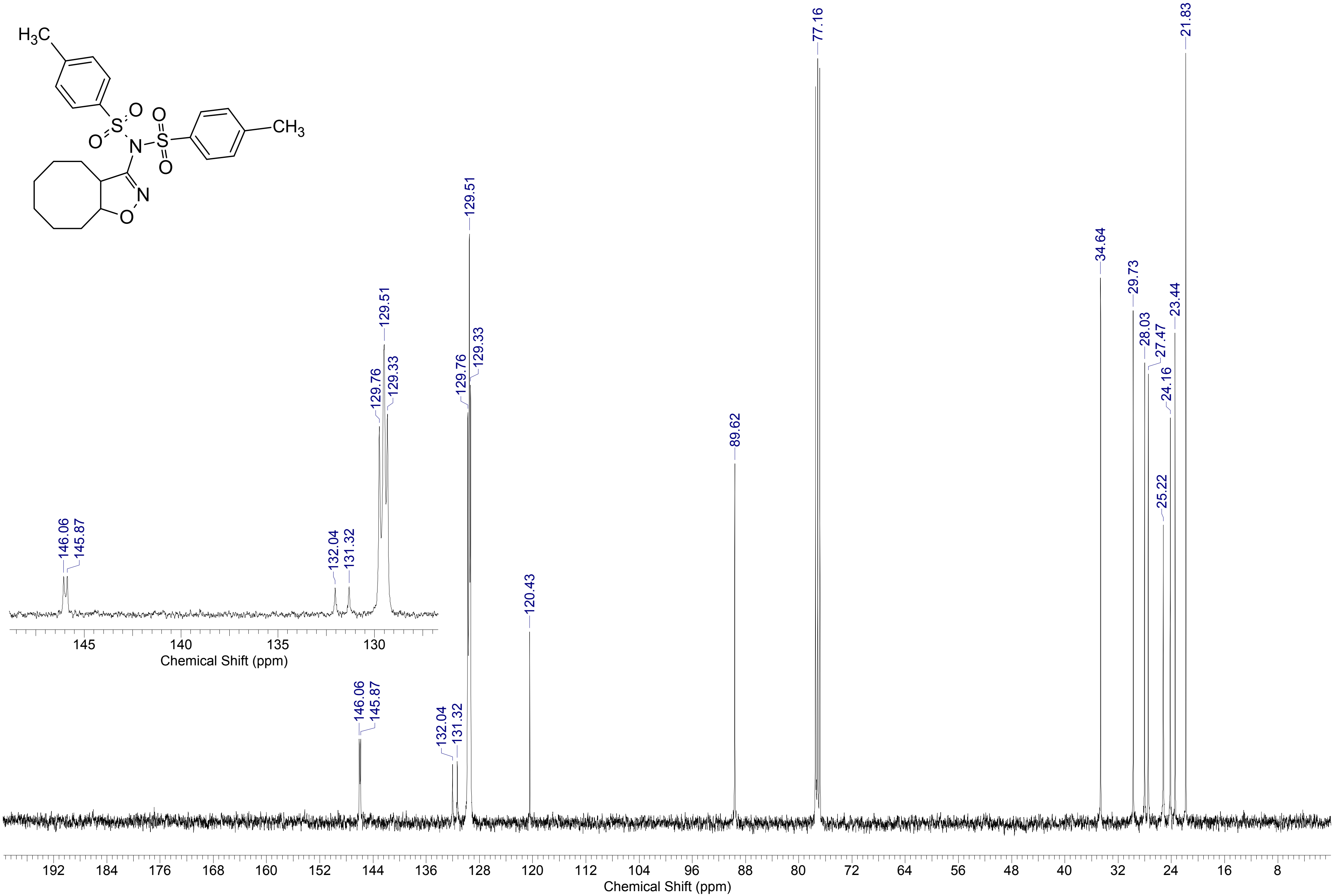

HSQC NMR spectrum of compound **5d** ($CDCl_3$)

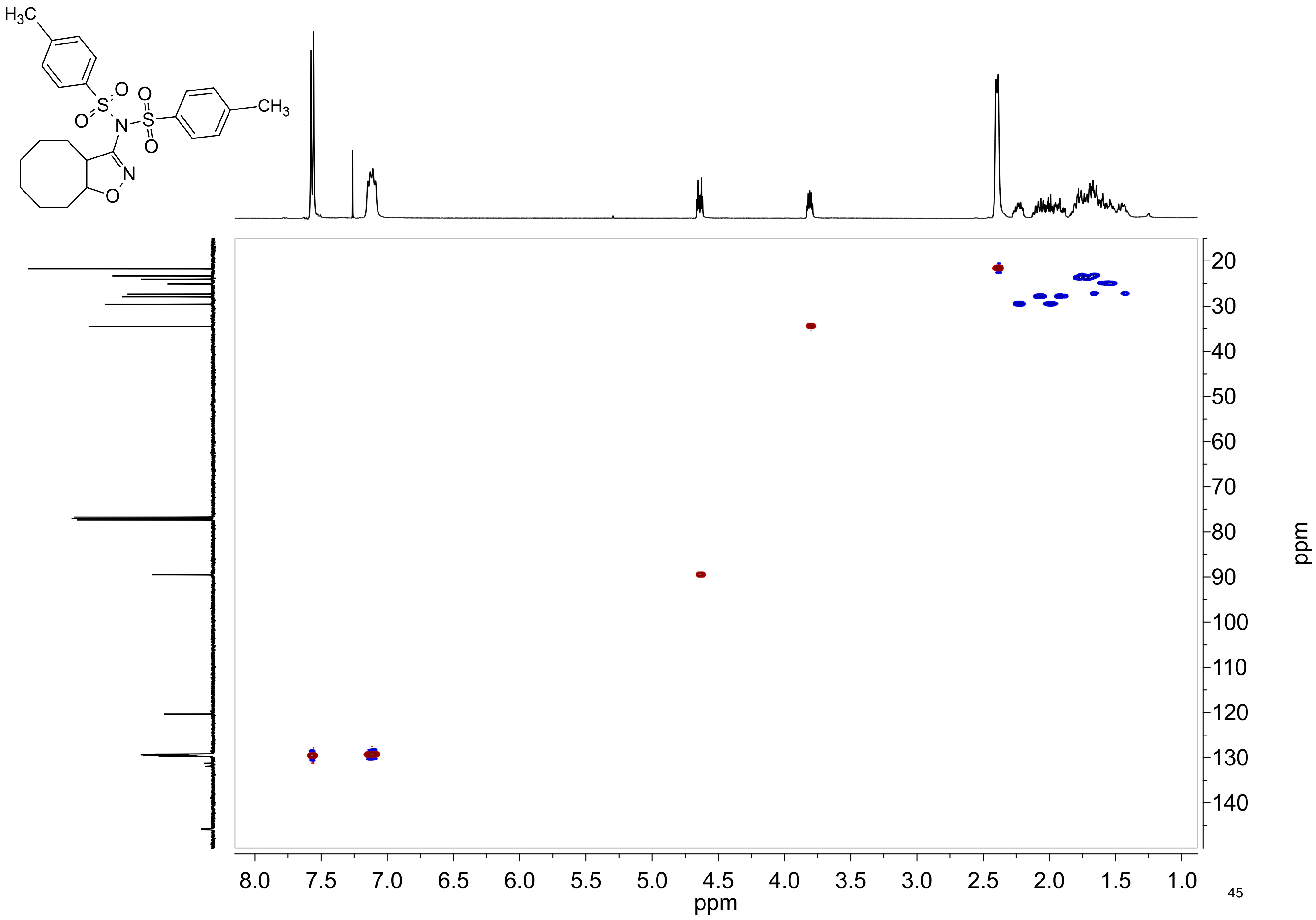

HMBC NMR spectrum of compound **5d** ($CDCl_3$)

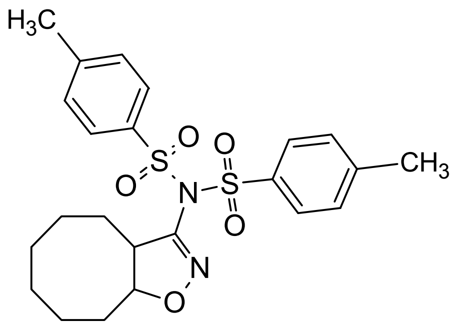

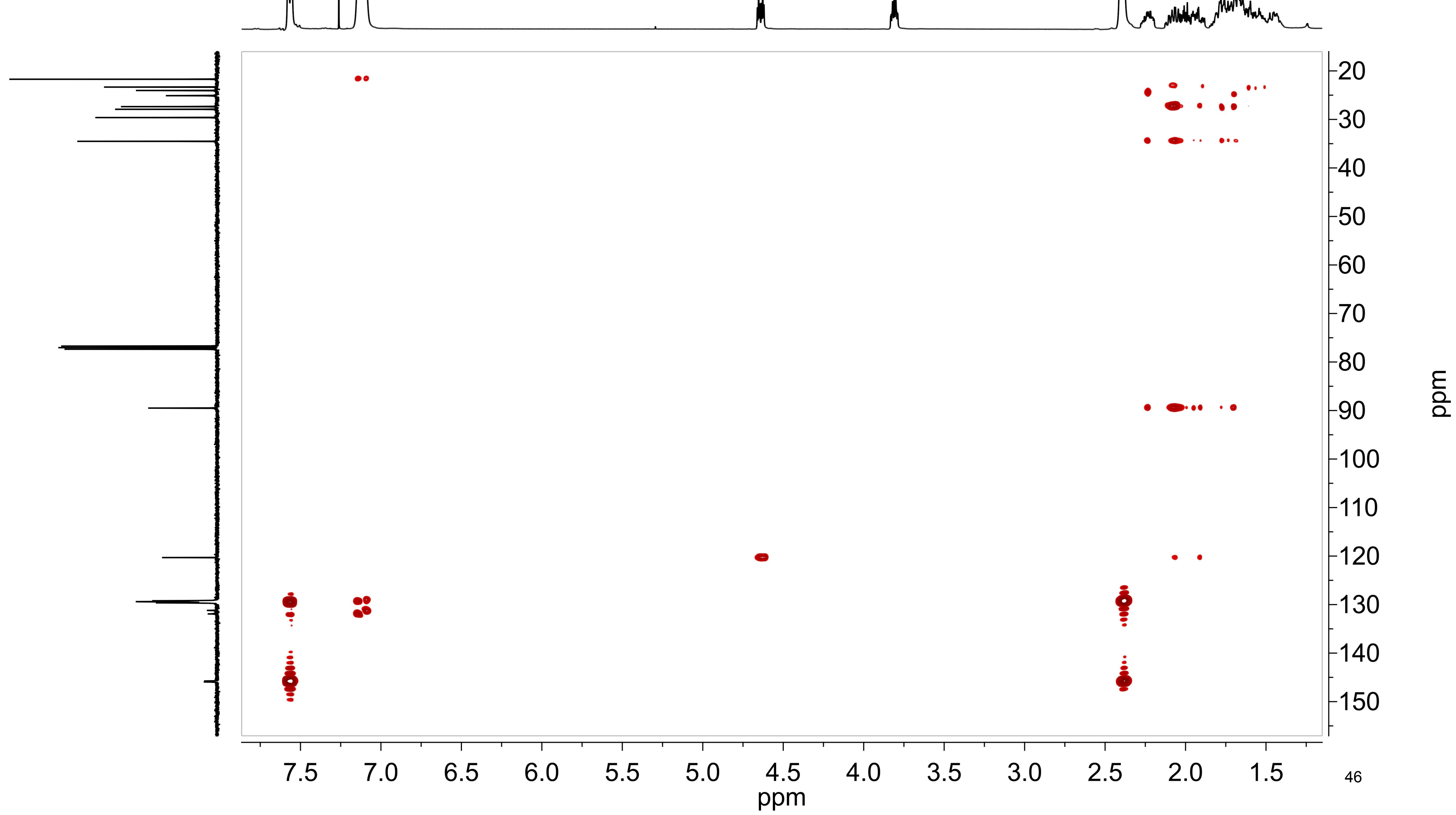

^{1}H NMR spectrum of compound **5e** ($CDCl_3$)

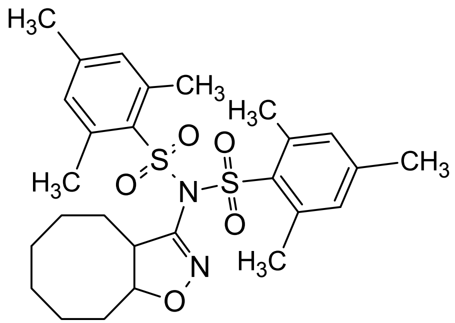

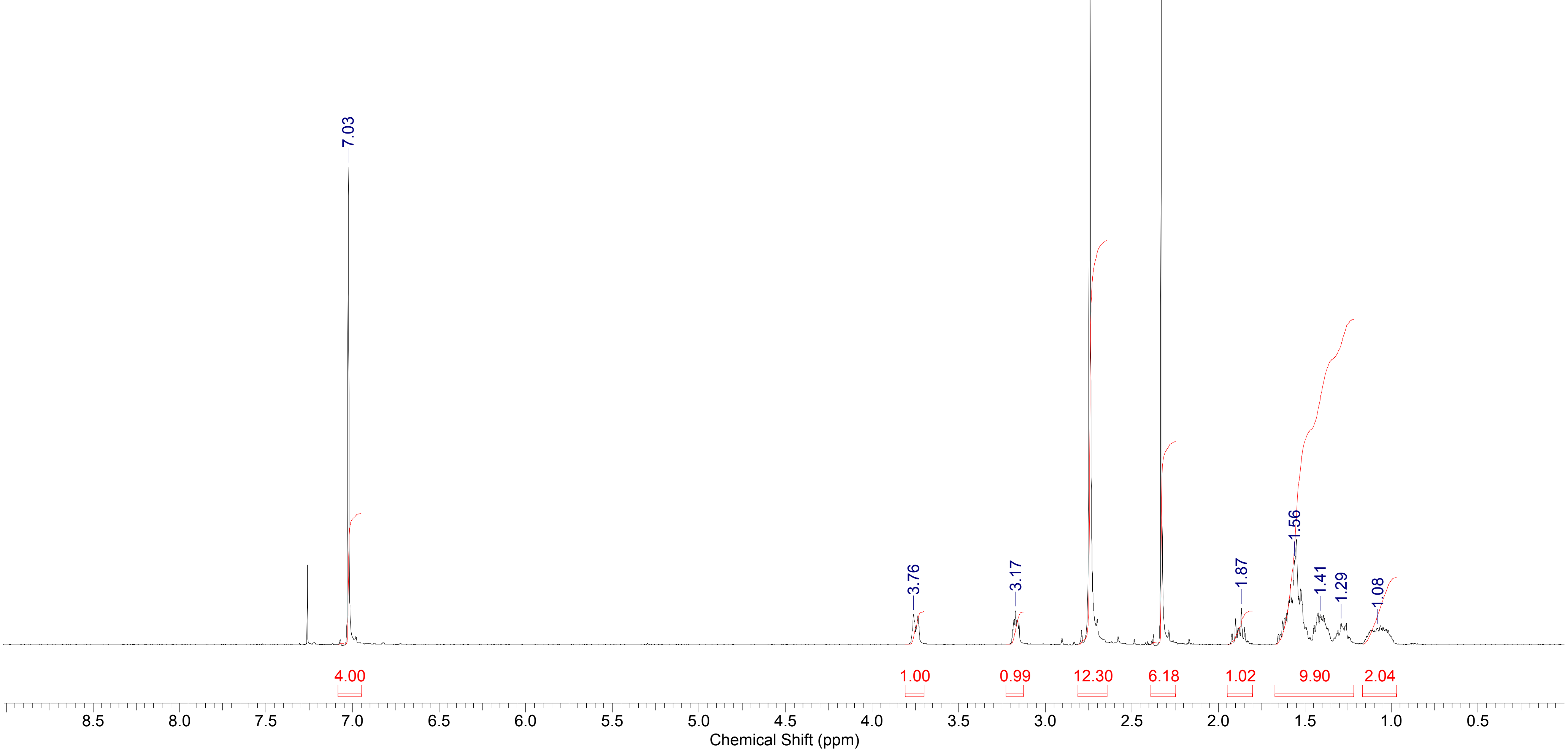

^{13}C NMR spectrum of compound **5e** ($CDCl_3$)

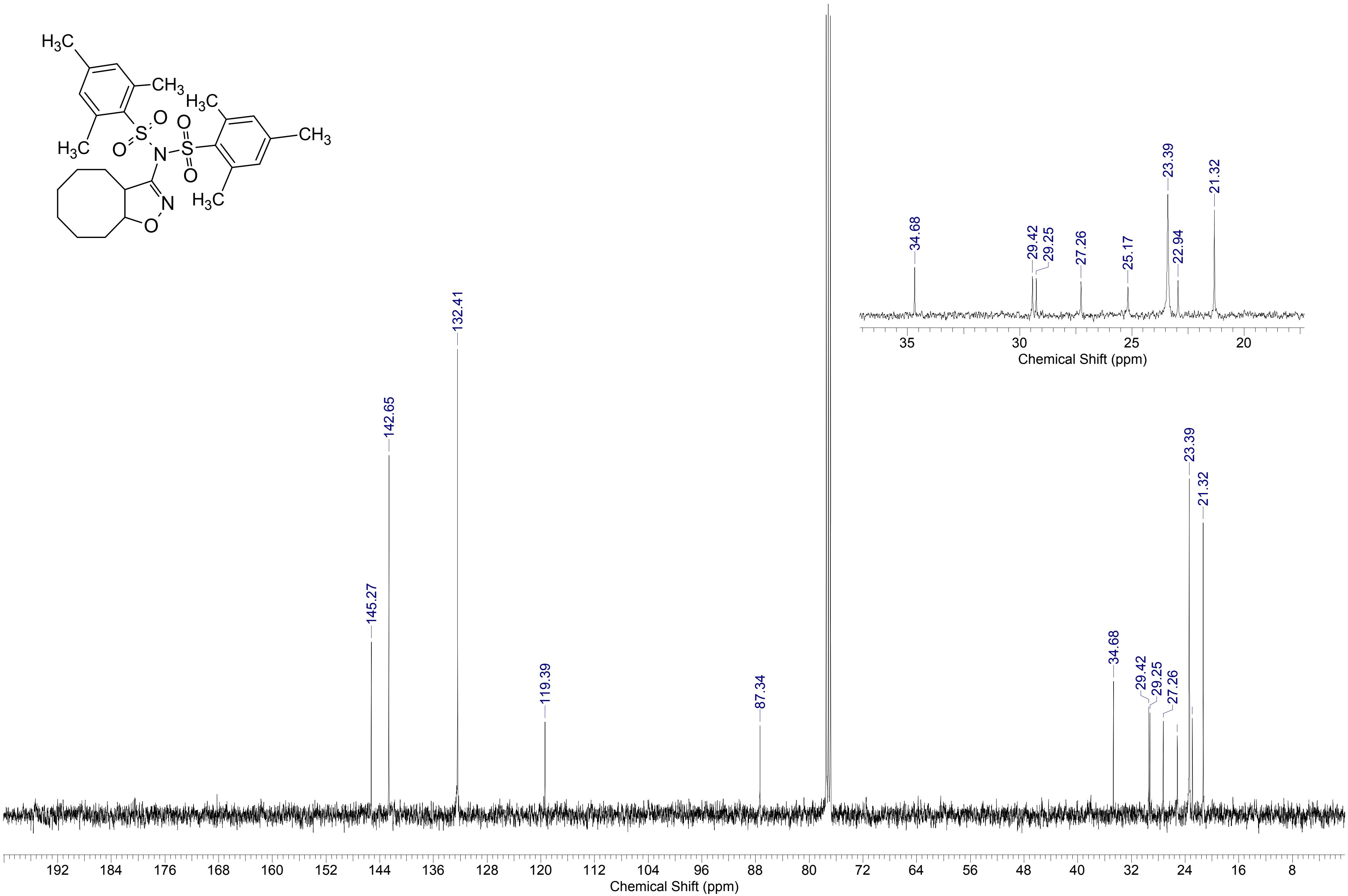

^{1}H NMR spectrum of compound **5f** ($CDCl_3$)

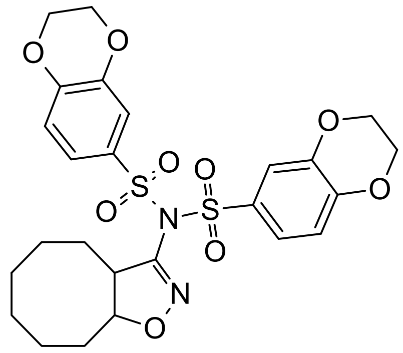

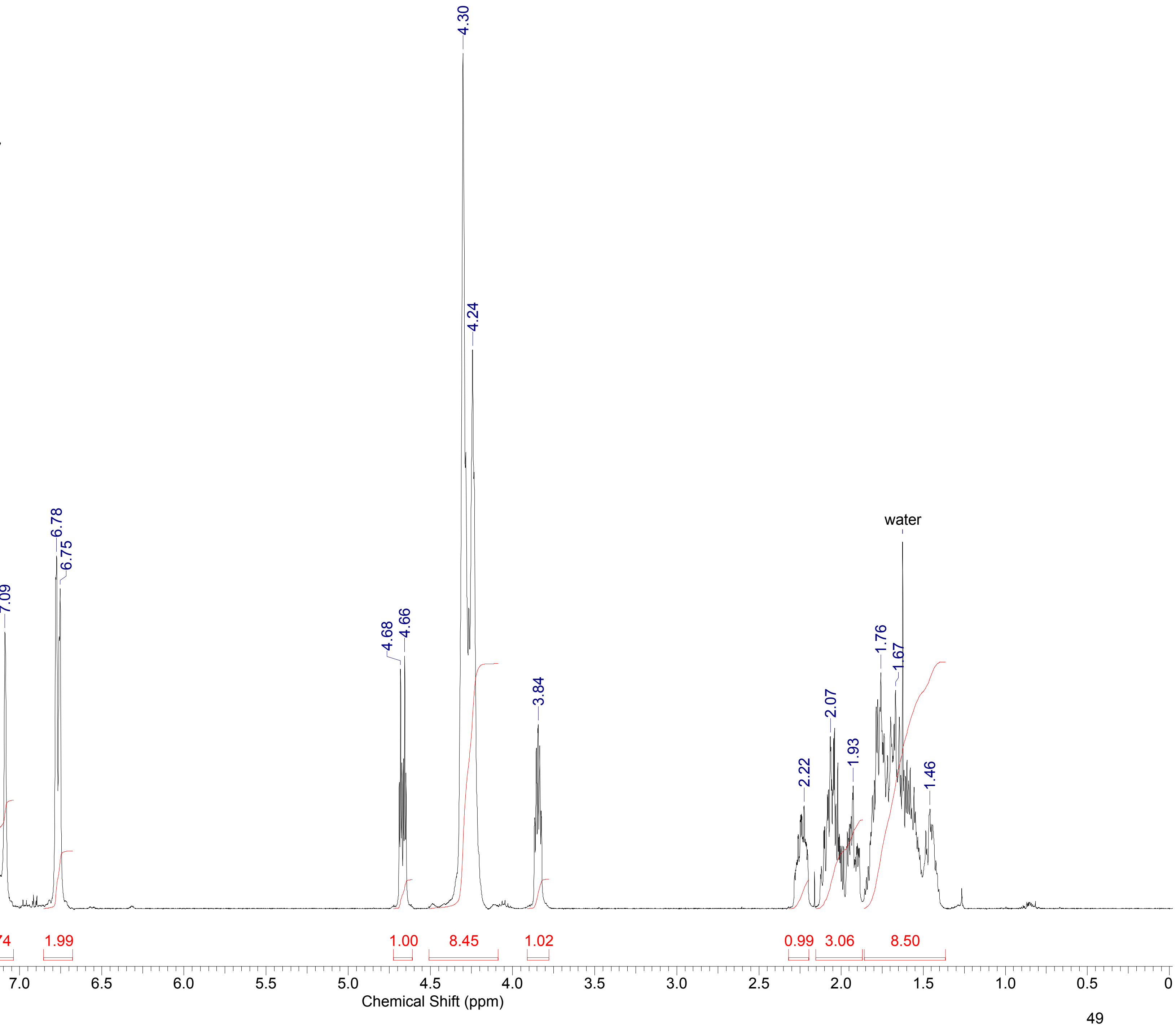

^{13}C NMR spectrum of compound **5f** ($CDCl_3$)

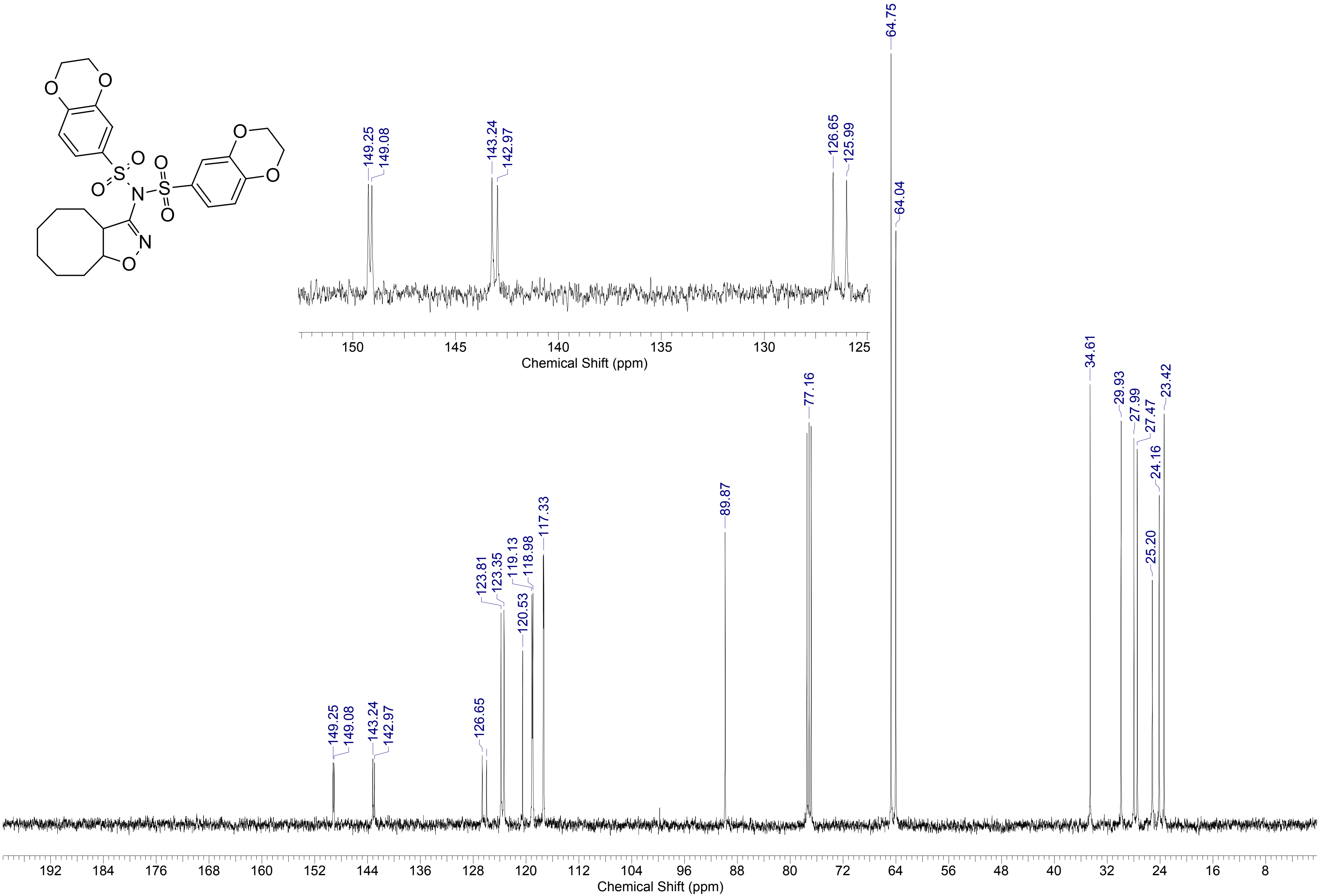

HSQC NMR spectrum of compound **5f** ($CDCl_3$)

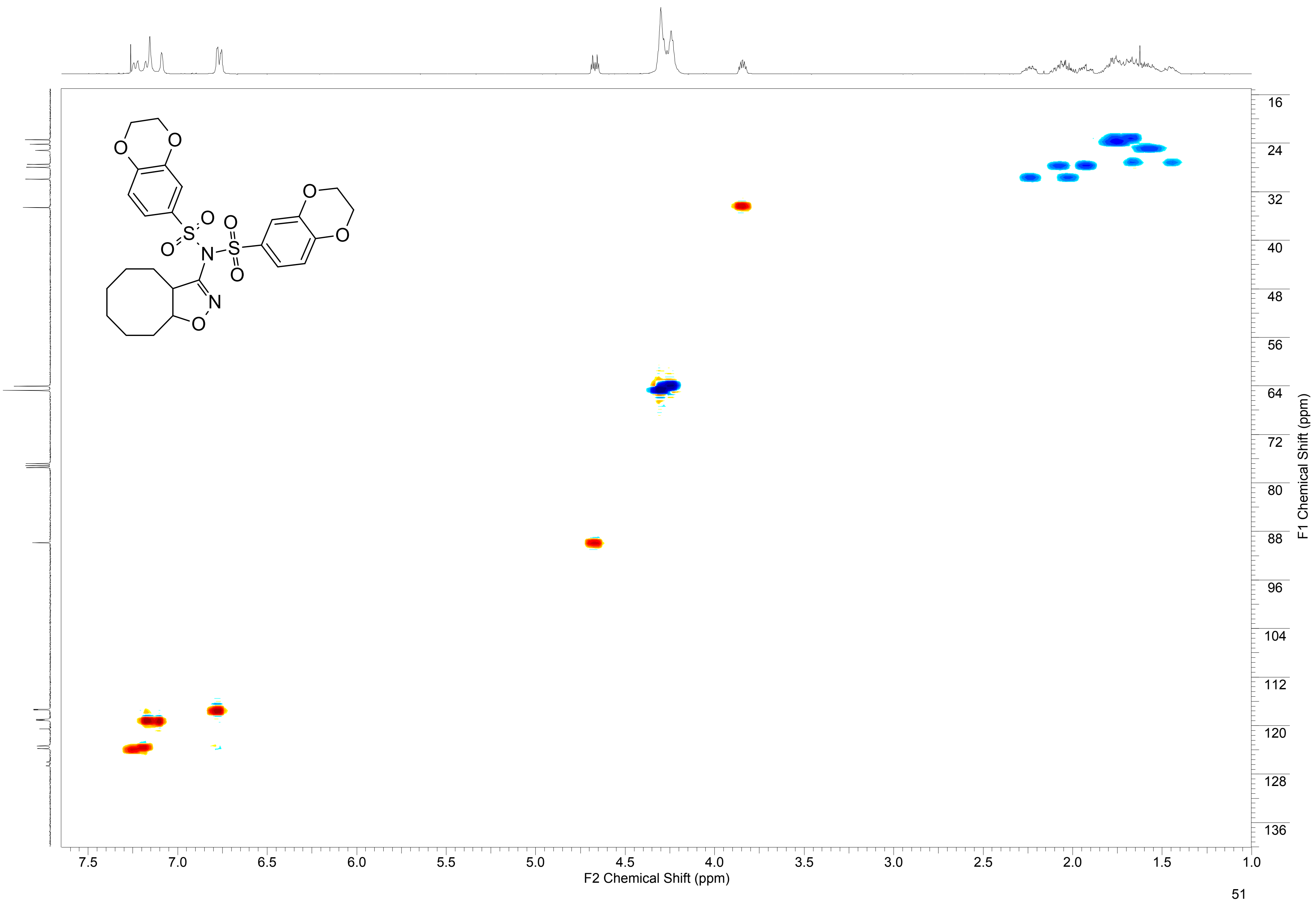

HMBC NMR spectrum of compound **5f** ($CDCl_3$)

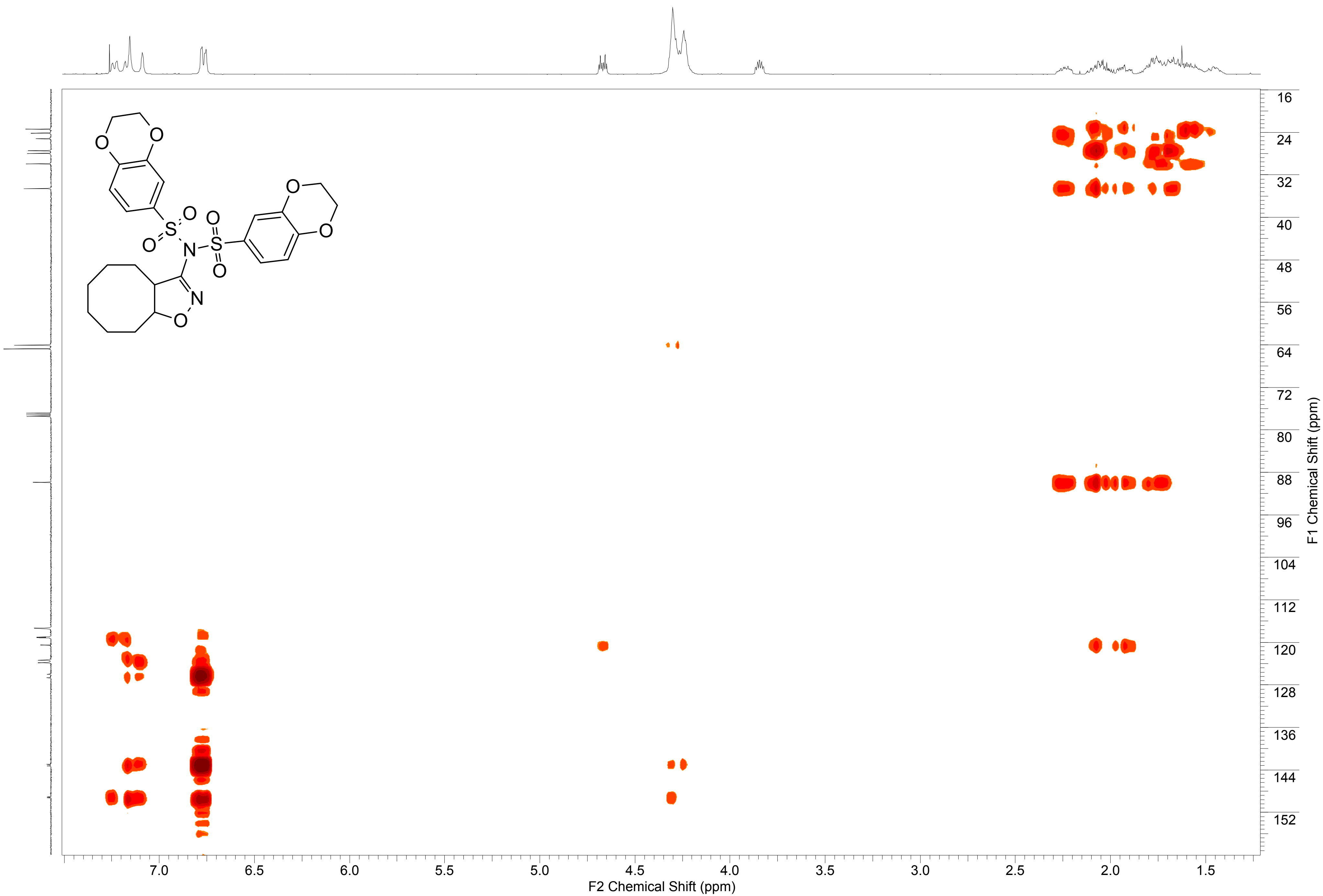

^{1}H NMR spectrum of compound **5g** ($CDCl_3$)

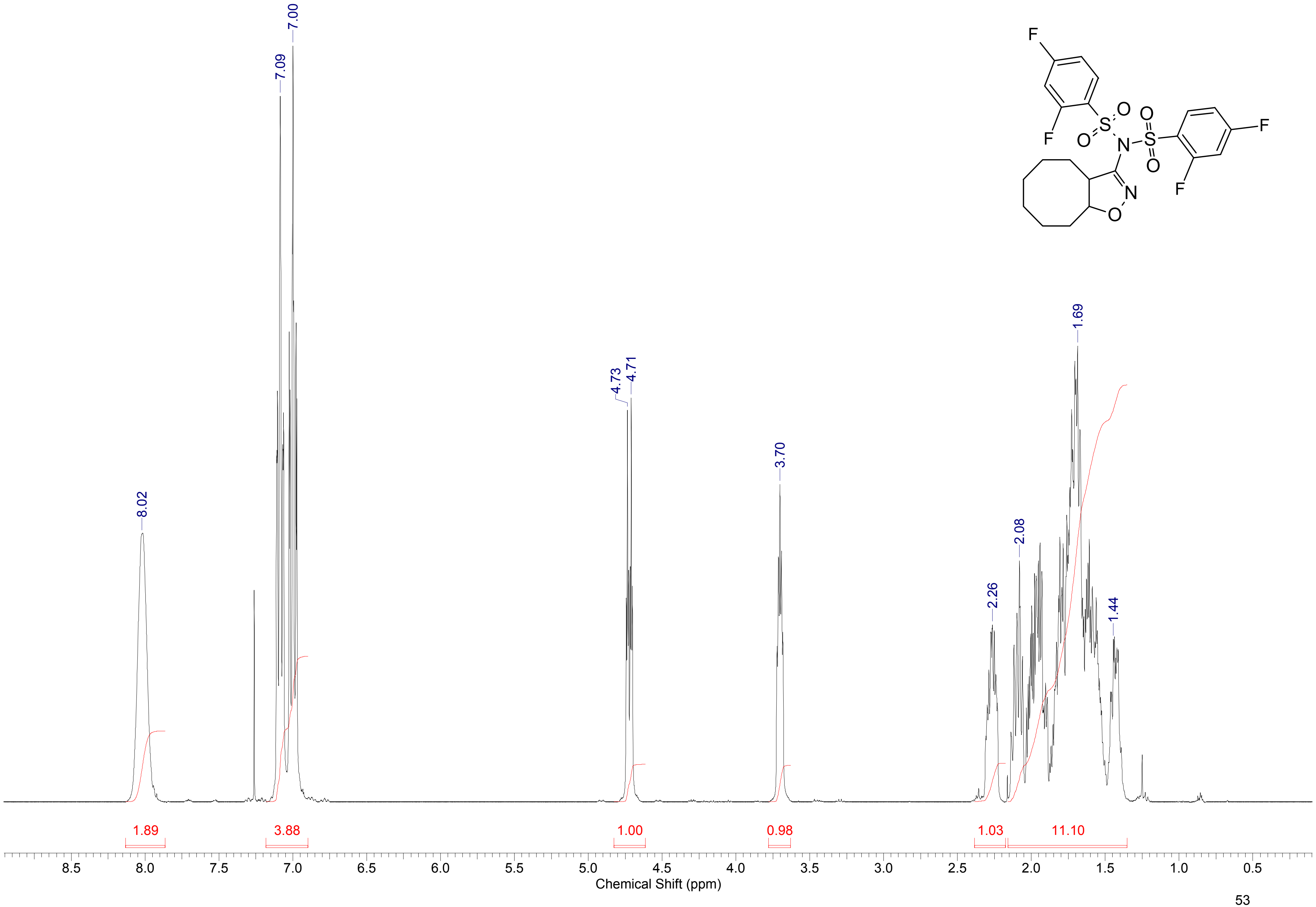

^{13}C NMR spectrum of compound **5g** ($CDCl_3$)

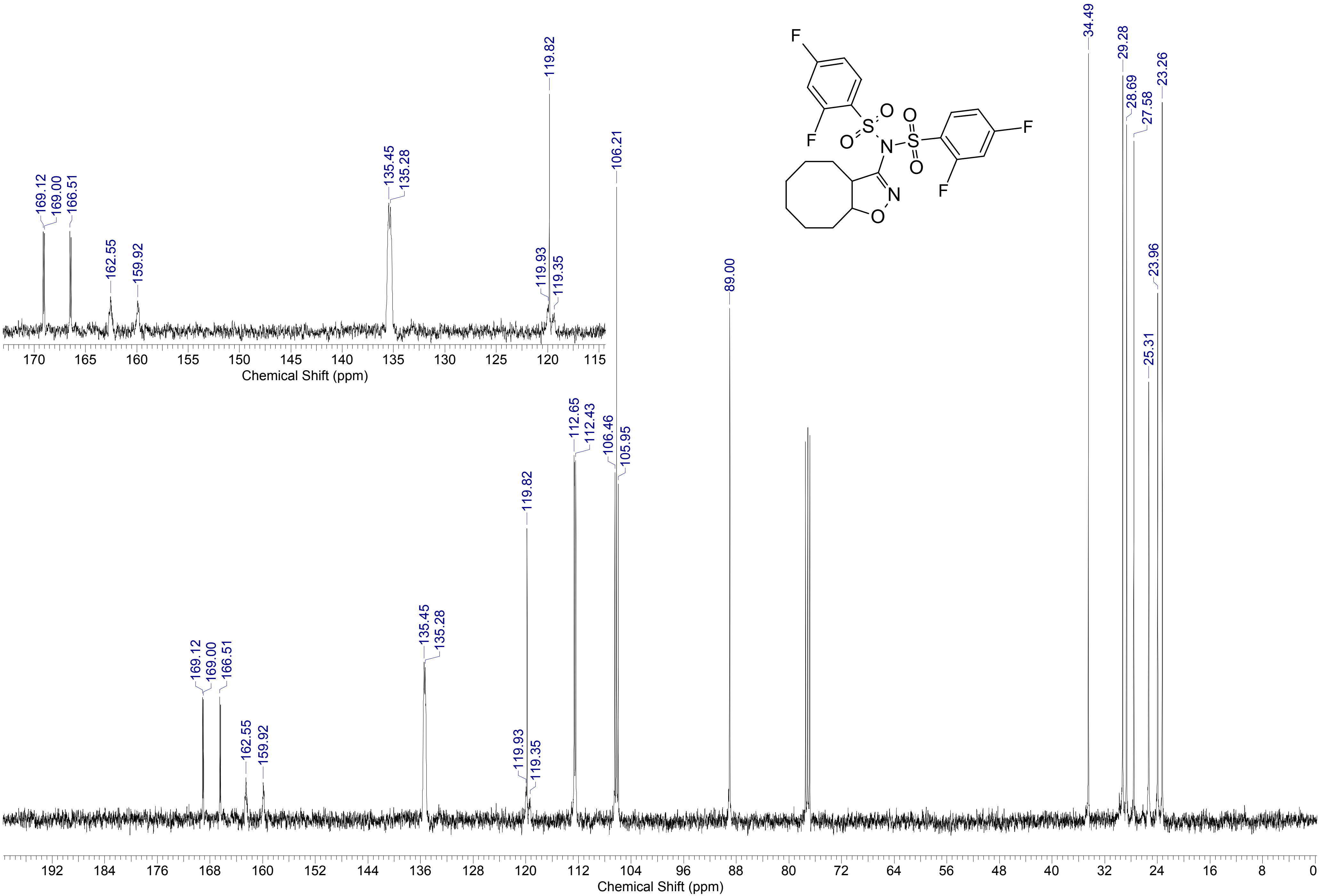

^{19}F NMR spectrum of compound **5g** ($CDCl_3$)

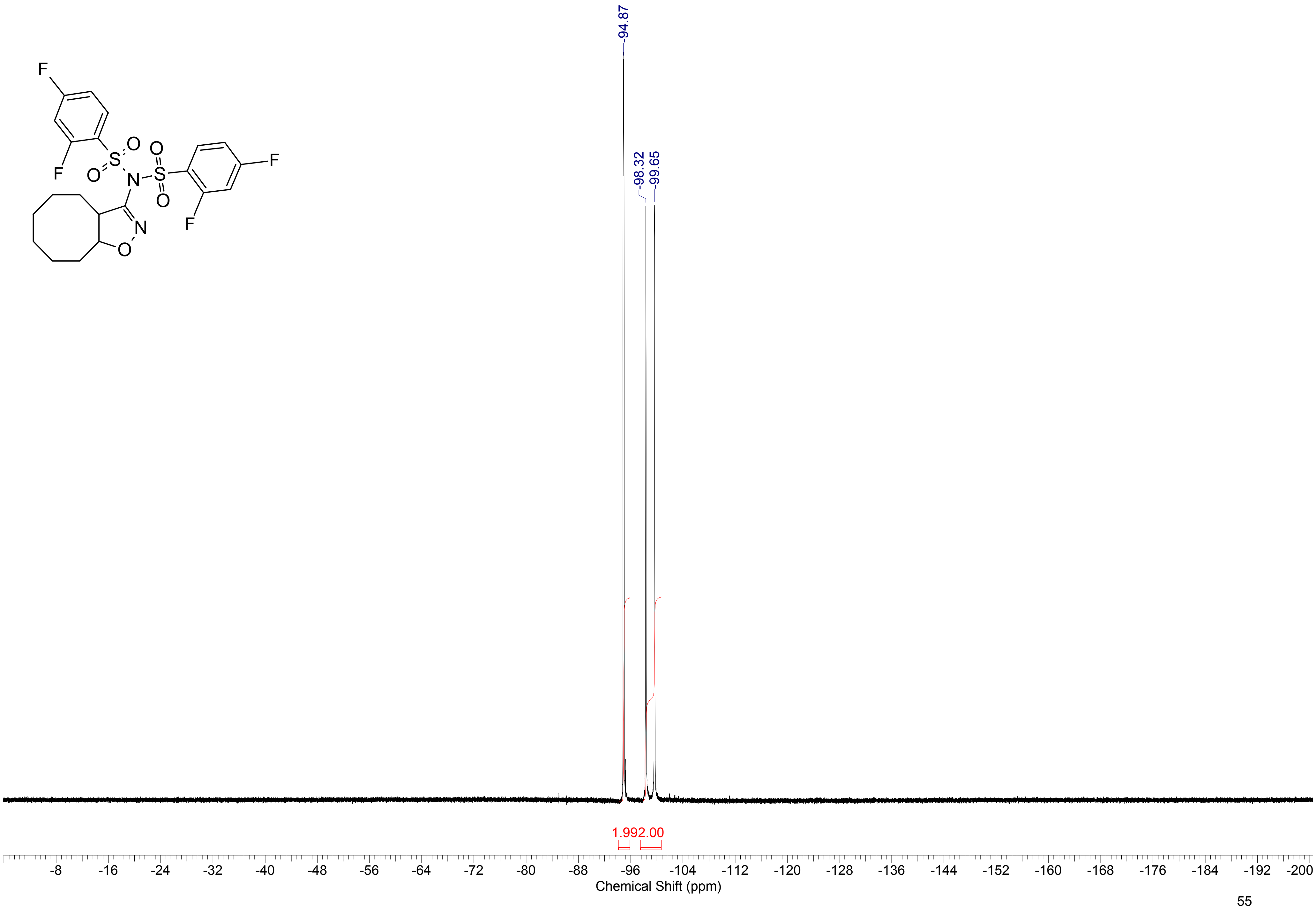

HSQC NMR spectrum of compound **5g**($CDCl_3$)

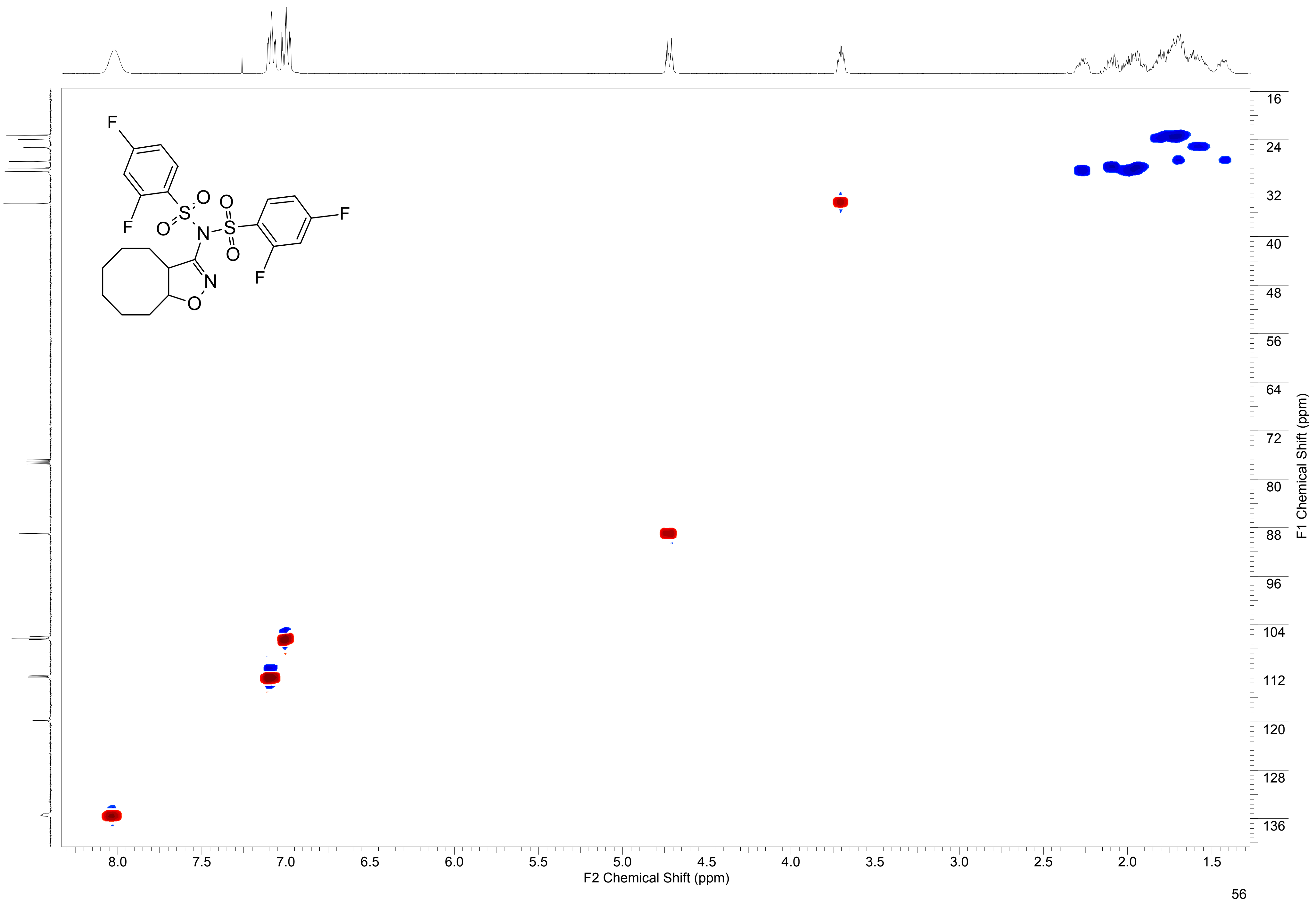

HMBC NMR spectrum of compound **5g** ($CDCl_3$)

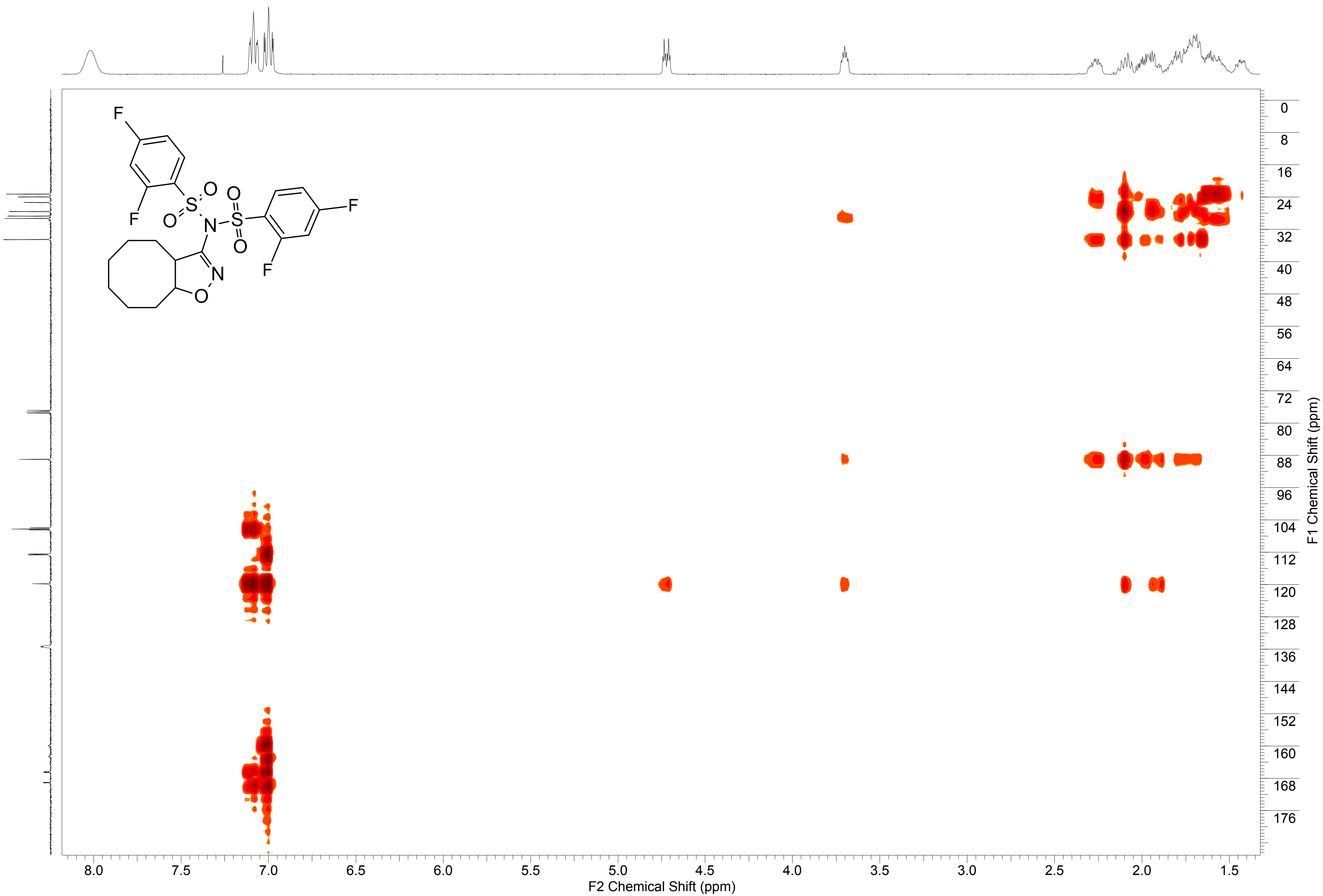

^{1}H NMR spectrum of compound **5h** ($CDCl_3$)

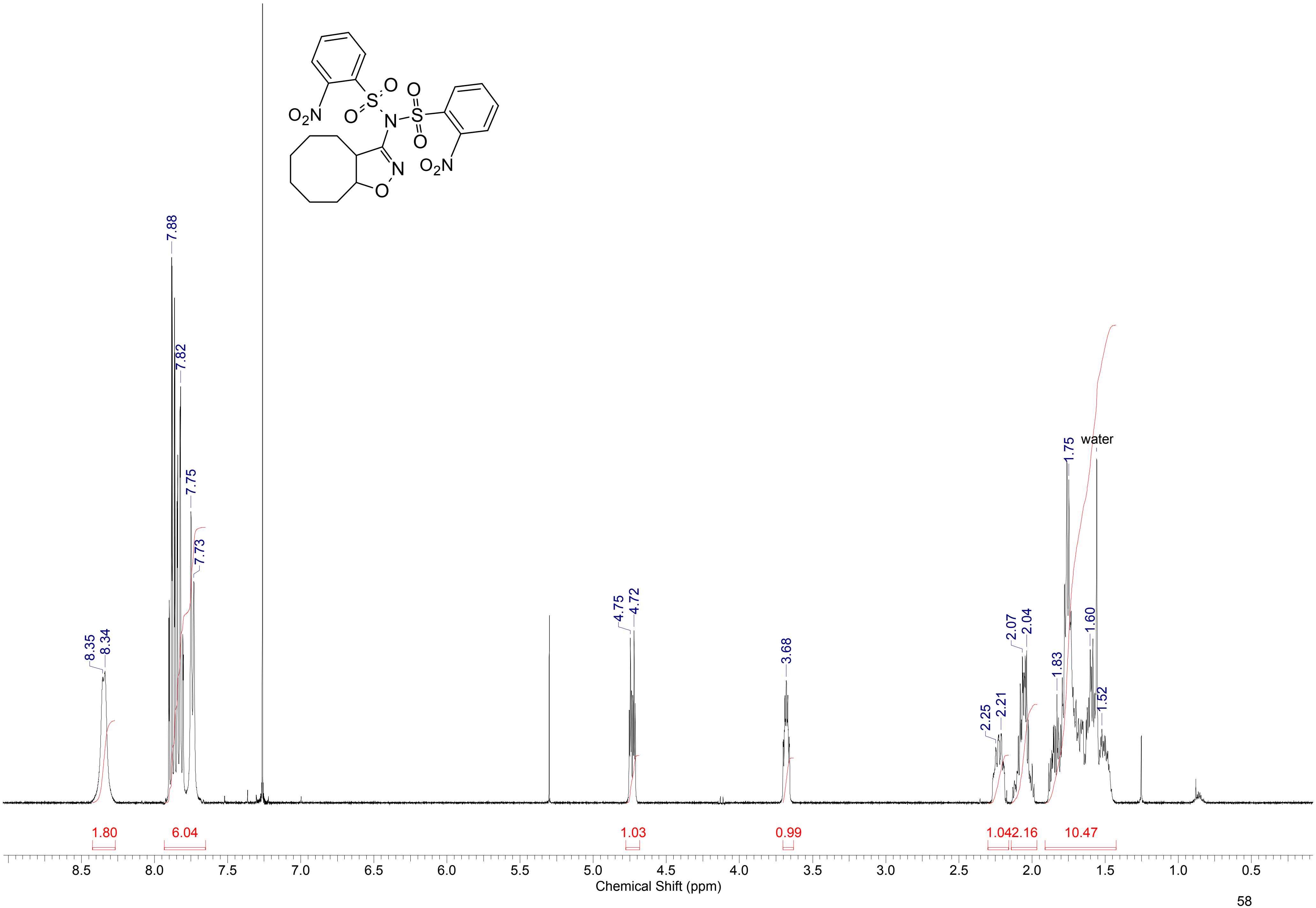

^{13}C NMR spectrum of compound **5h** ($CDCl_3$)

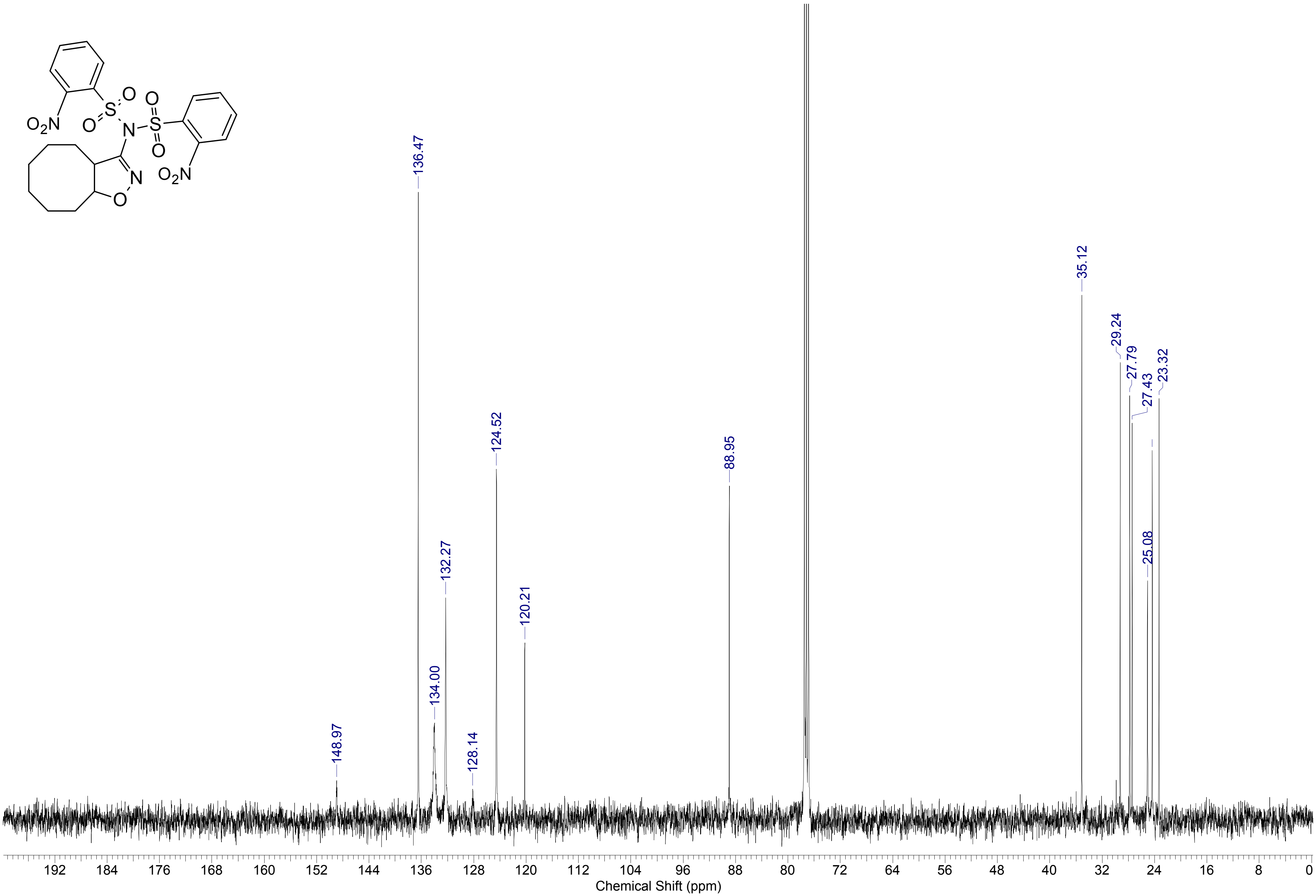

HMBC NMR spectrum of compound **5h** ($CDCl_3$)

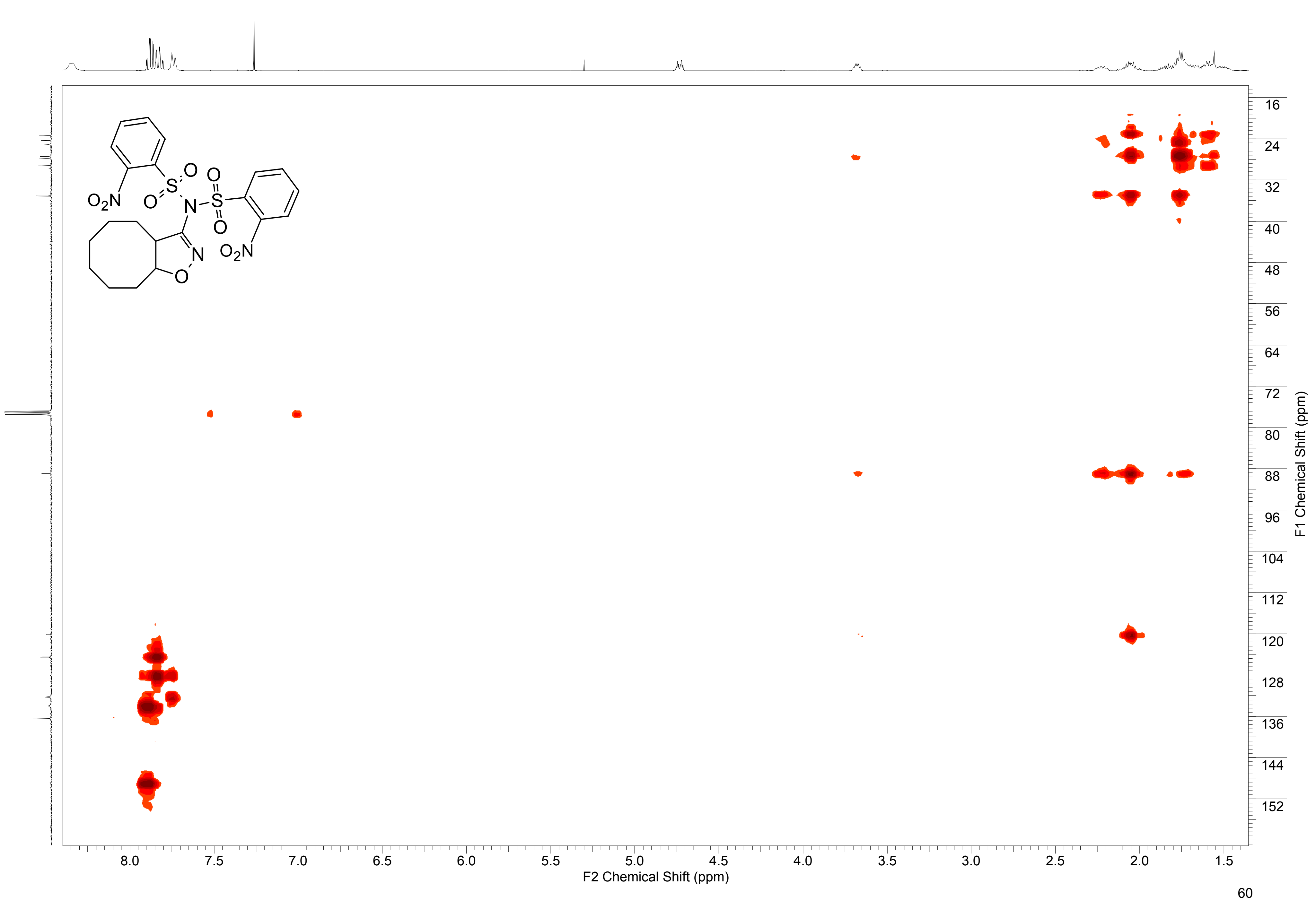

^{1}H NMR spectrum of compound **5i** ($CDCl_3$)

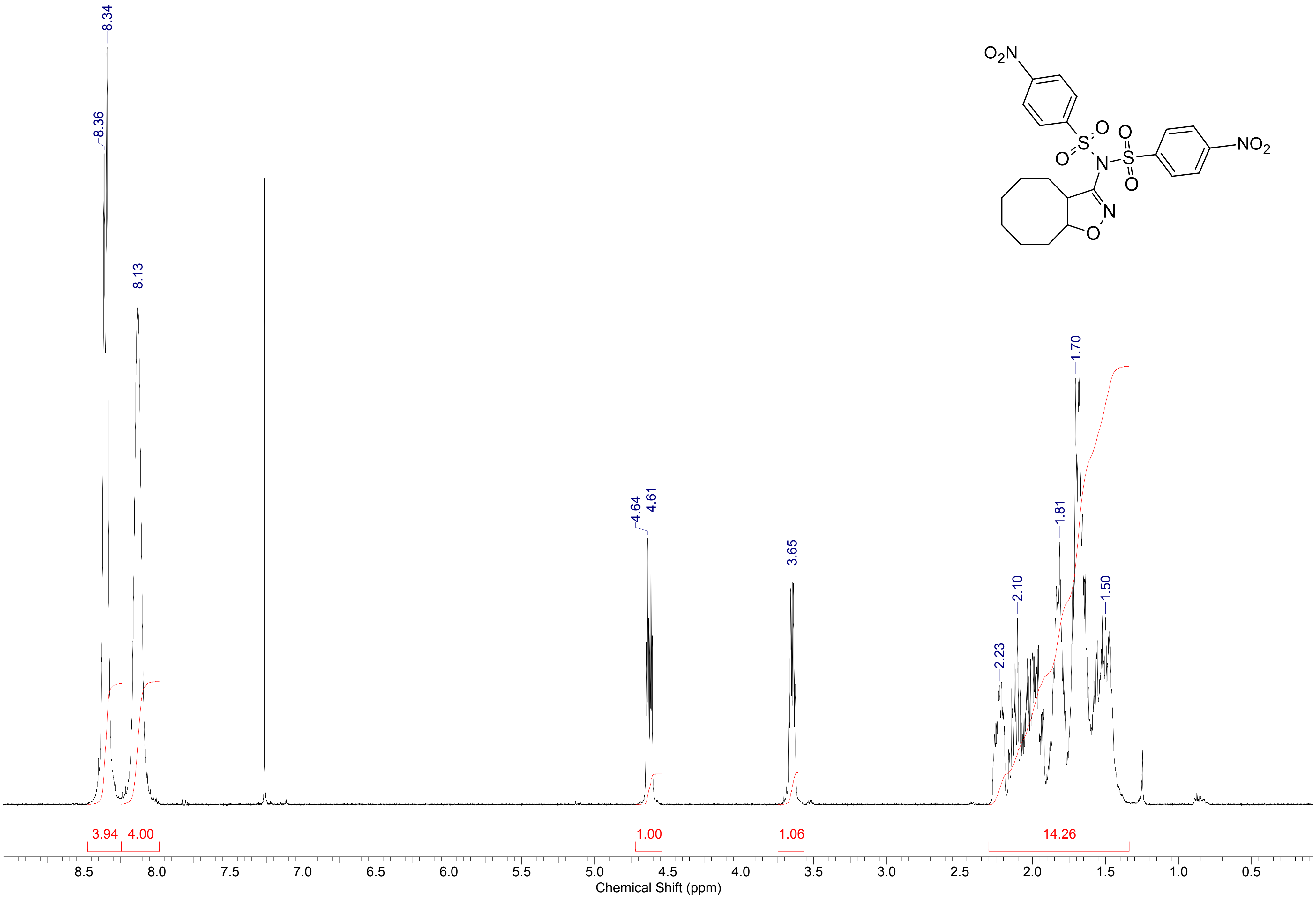

^{13}C NMR spectrum of compound **5i** ($CDCl_3$)

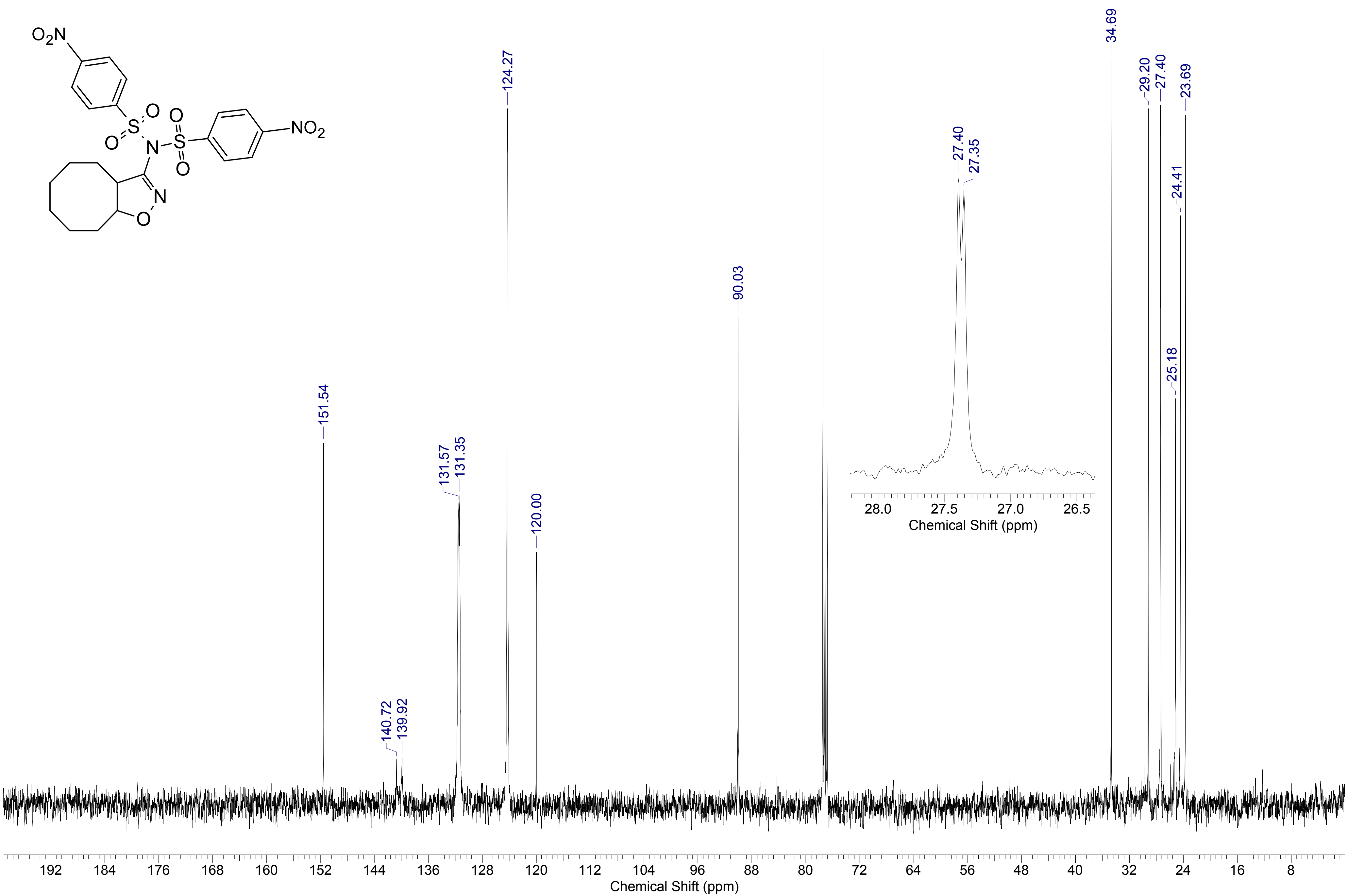

HSQC NMR spectrum of compound **5i** ($CDCl_3$)

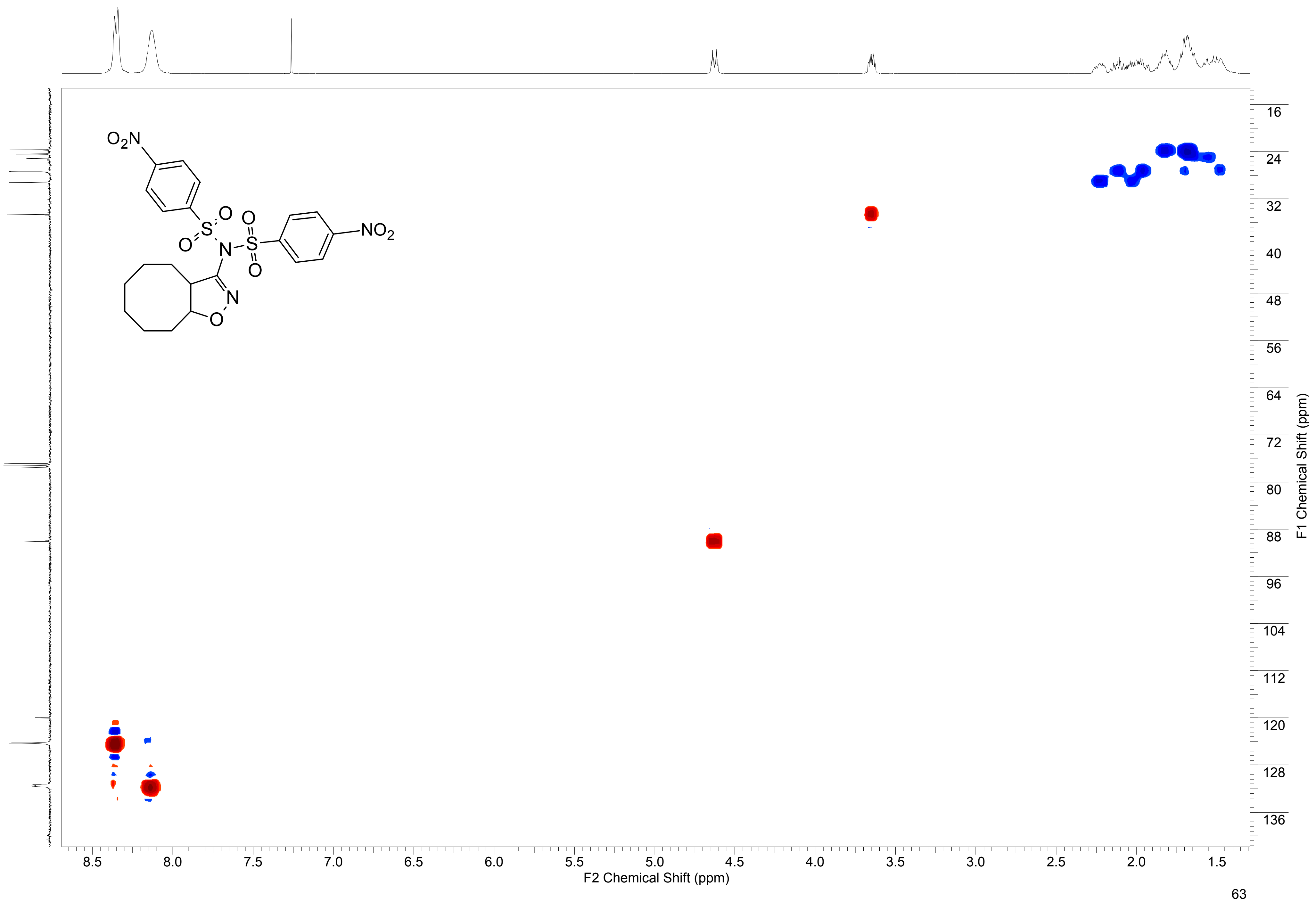

^{1}H NMR spectrum of compound **5j** ($CDCl_3$)

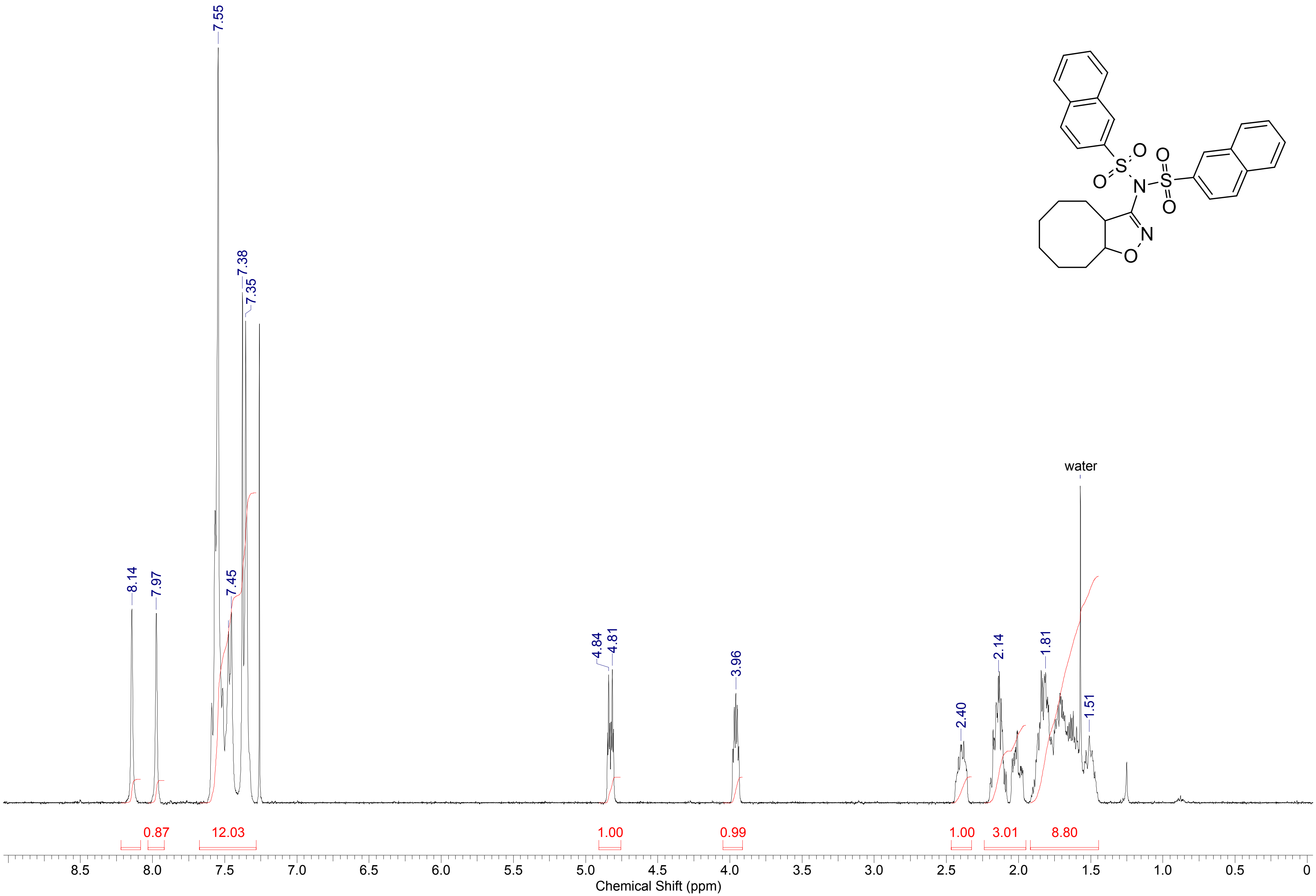

^{13}C NMR spectrum of compound **5j** ($CDCl_3$)

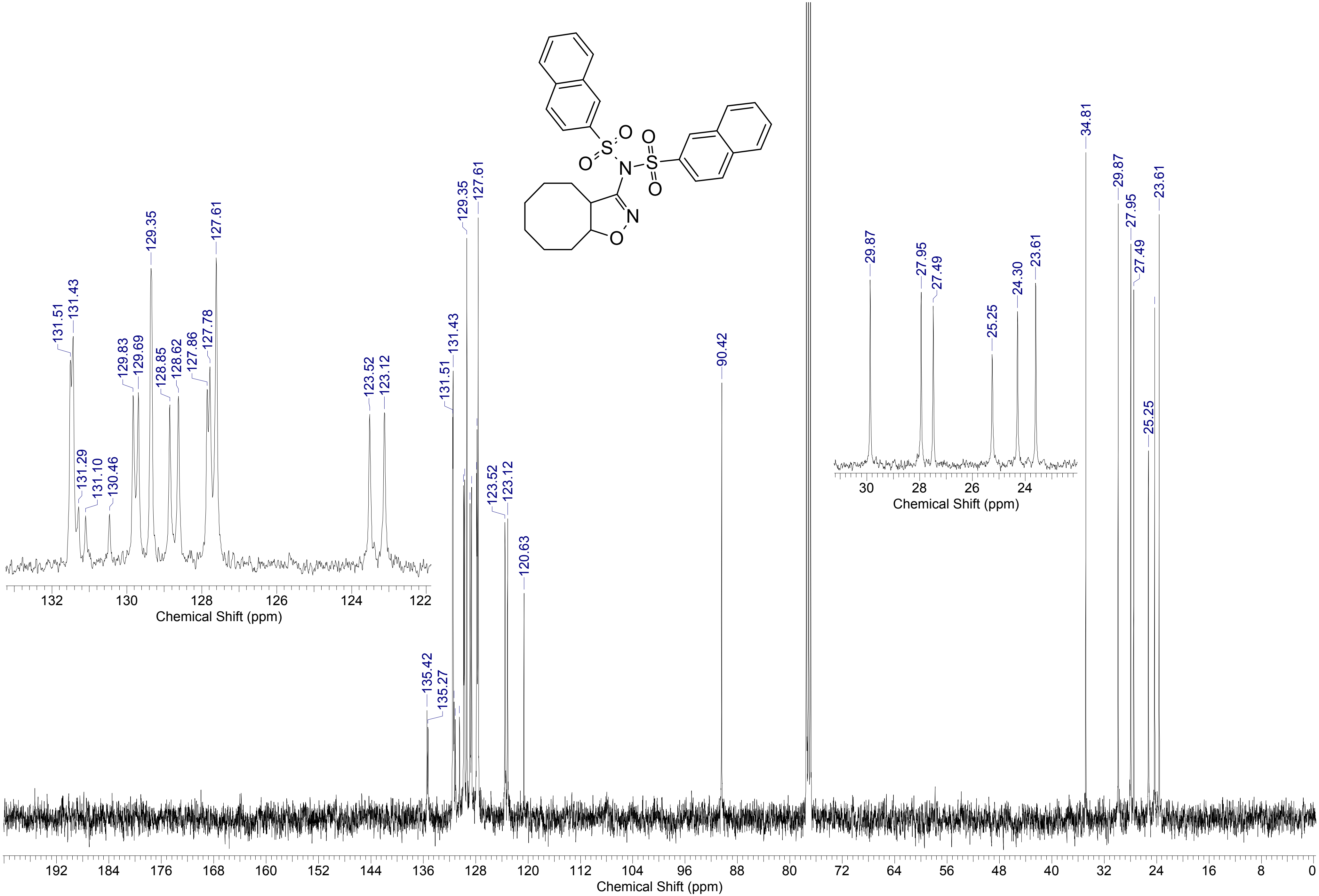

HSQC NMR spectrum of compound **5j** ($CDCl_3$)

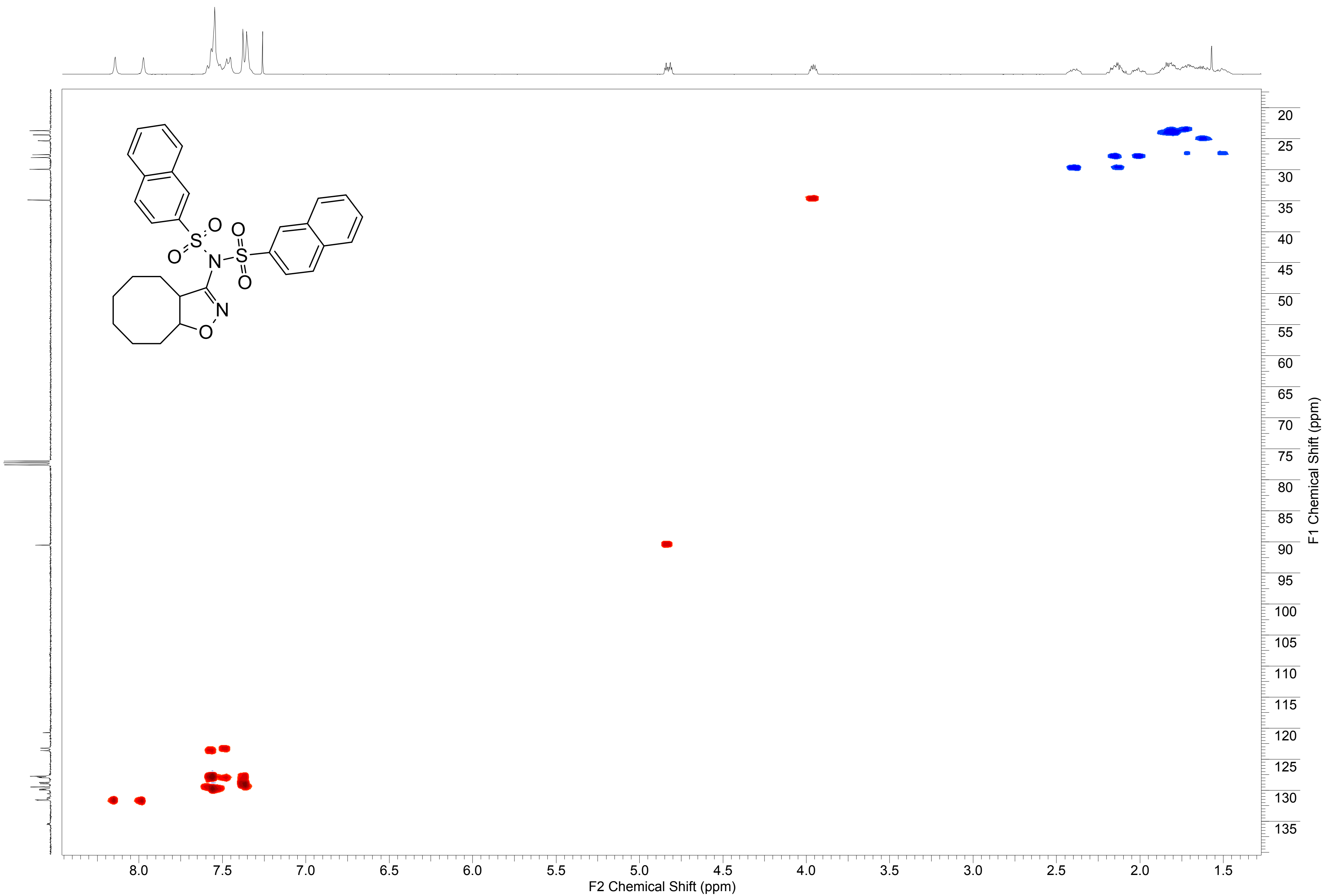

^1H NMR spectrum of compound **5k** ($CDCl_3$)

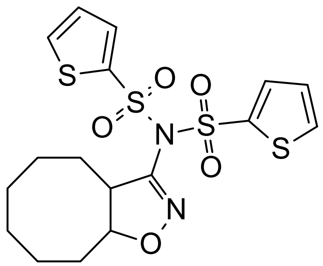

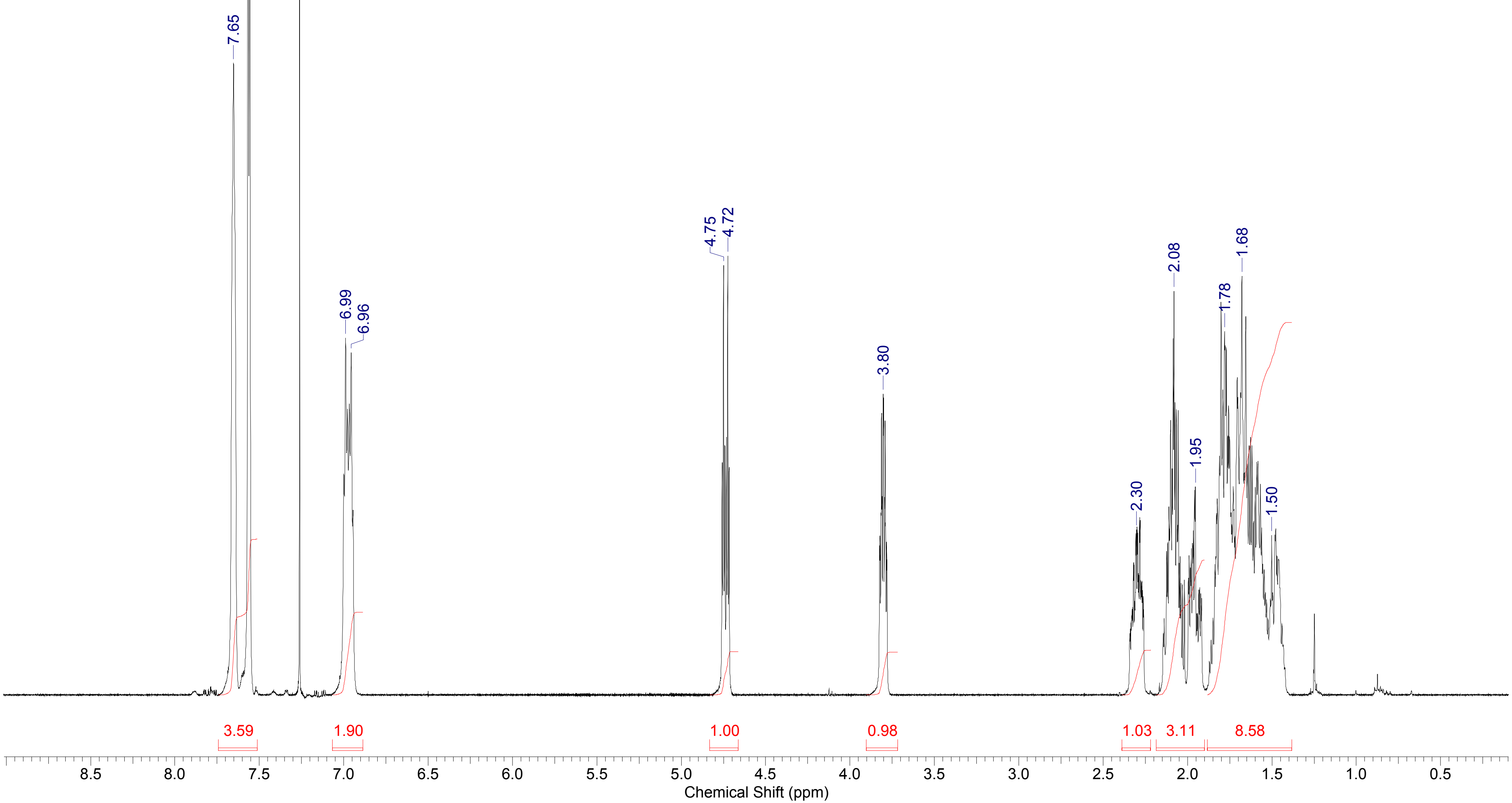

^{13}C NMR spectrum of compound **5k** ($CDCl_3$)

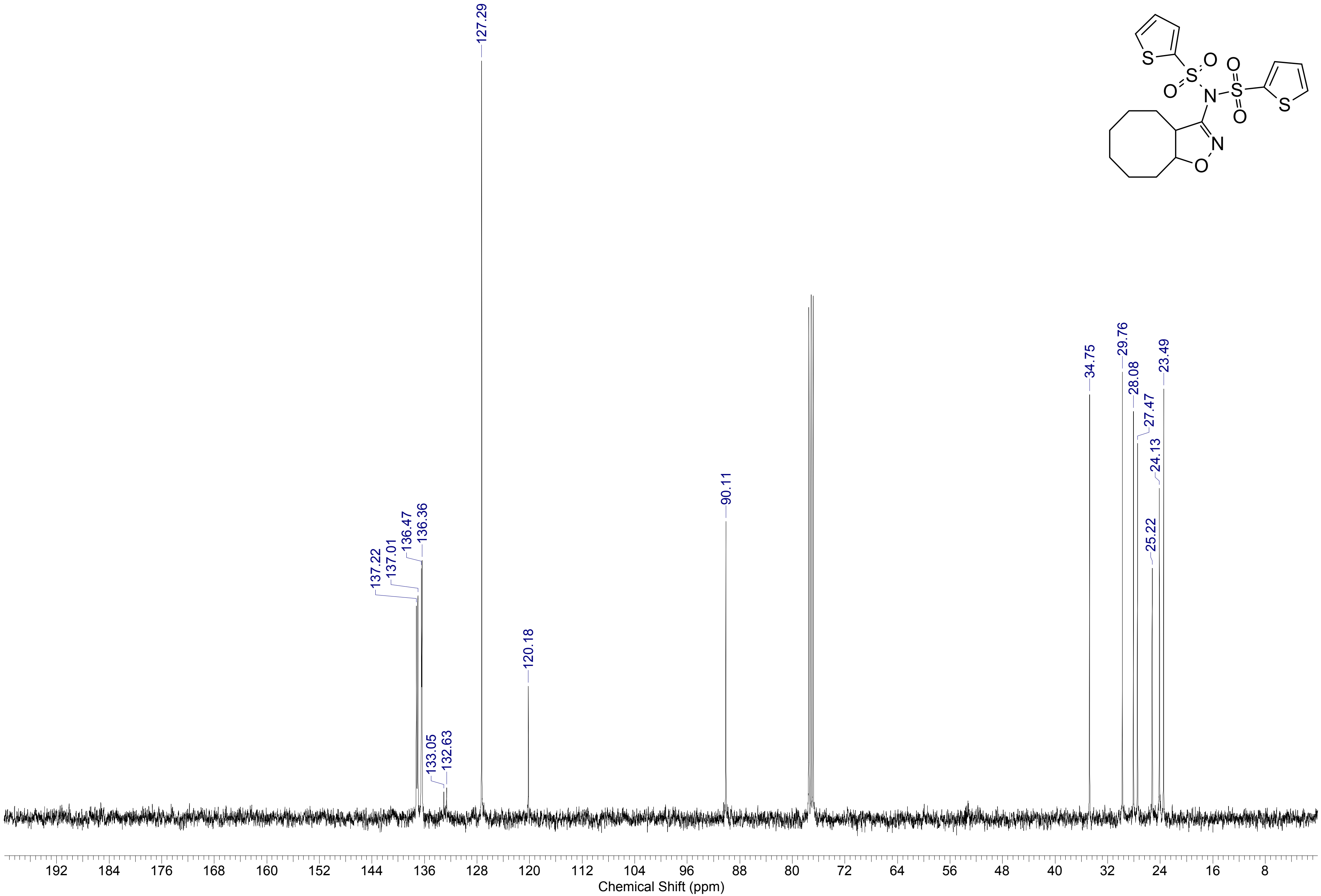

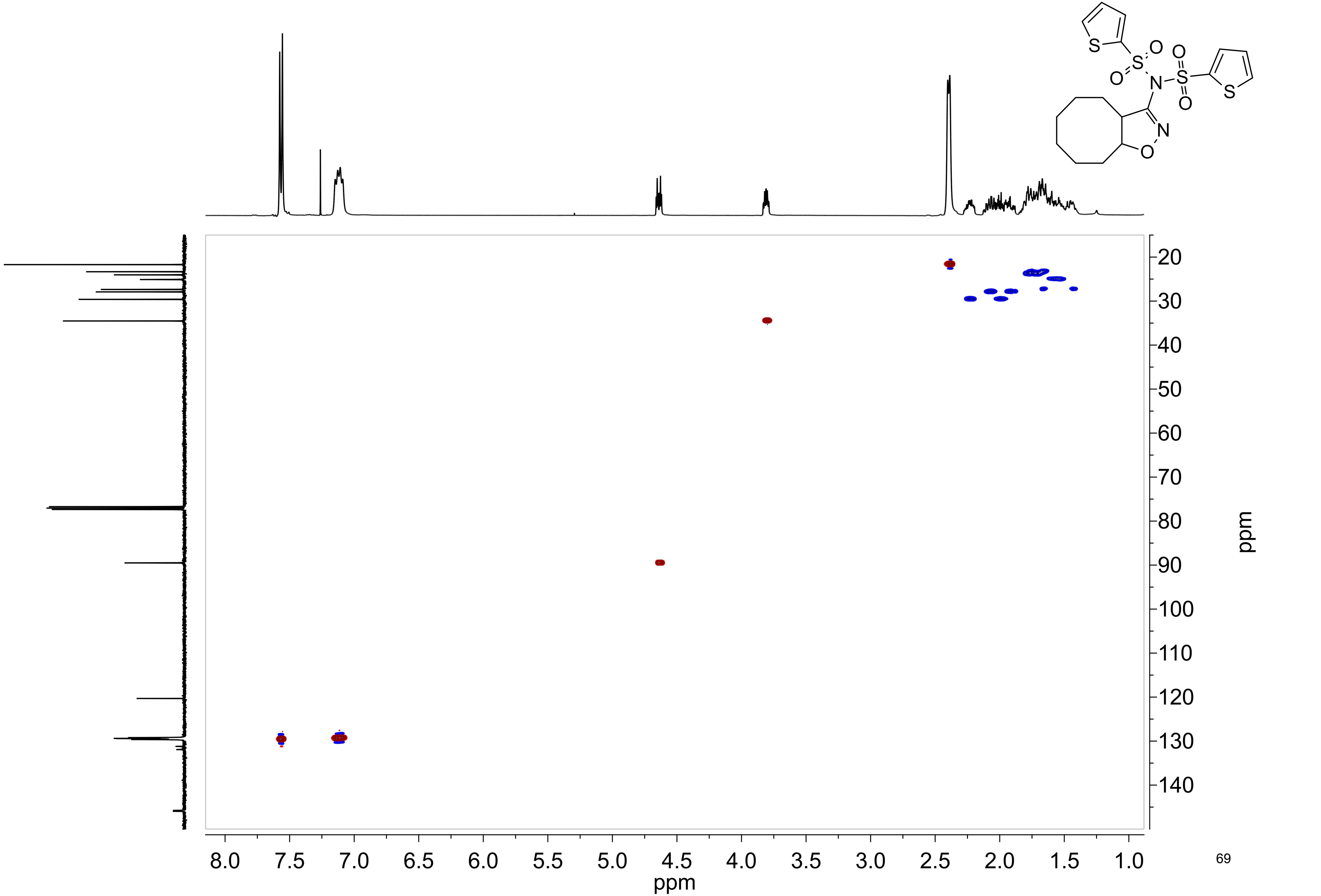

ppm
ppm

HMBC NMR spectrum of compound **5k** ($CDCl_3$)

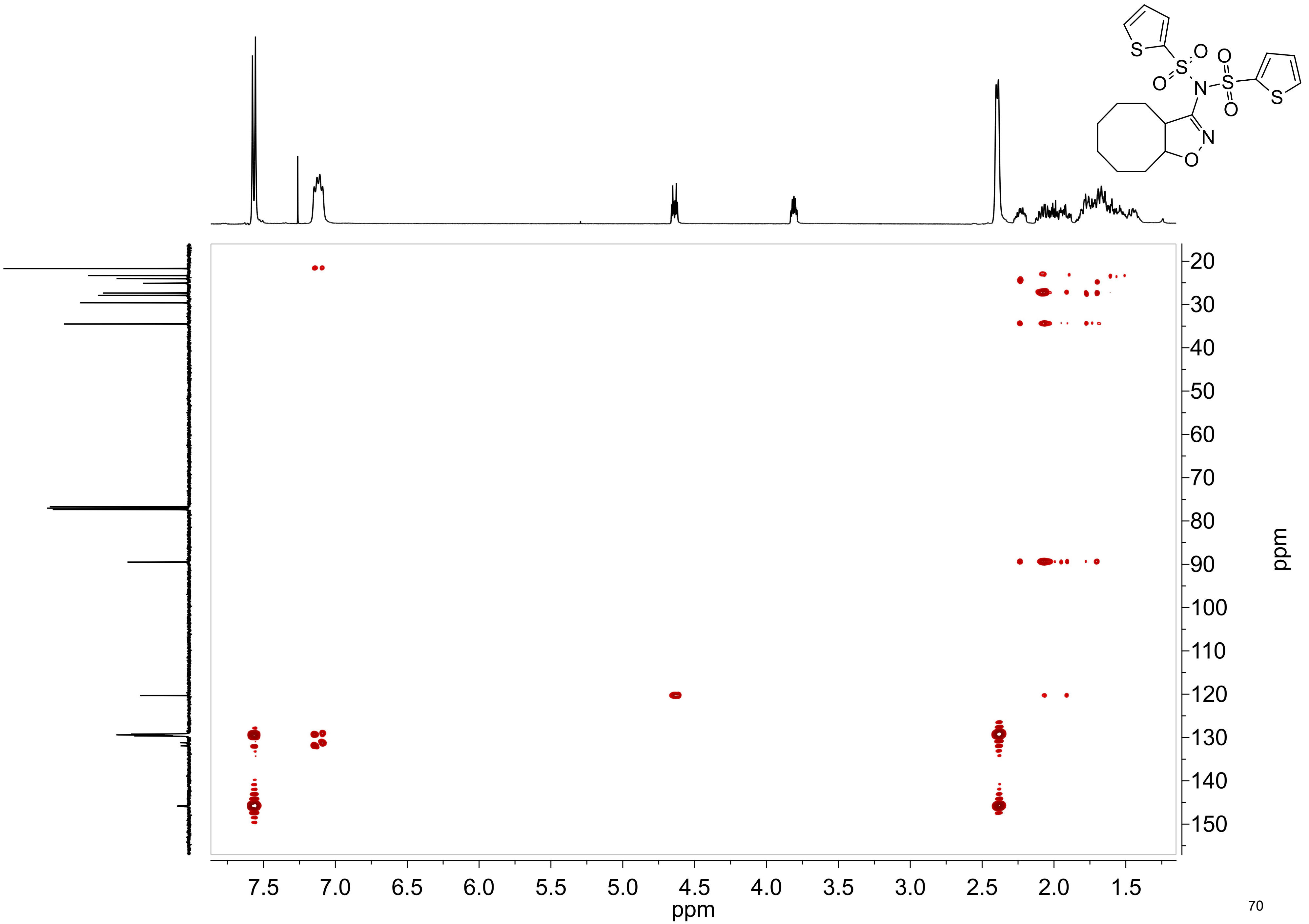

^{1}H NMR spectrum of compound **5l** ($CDCl_3$)

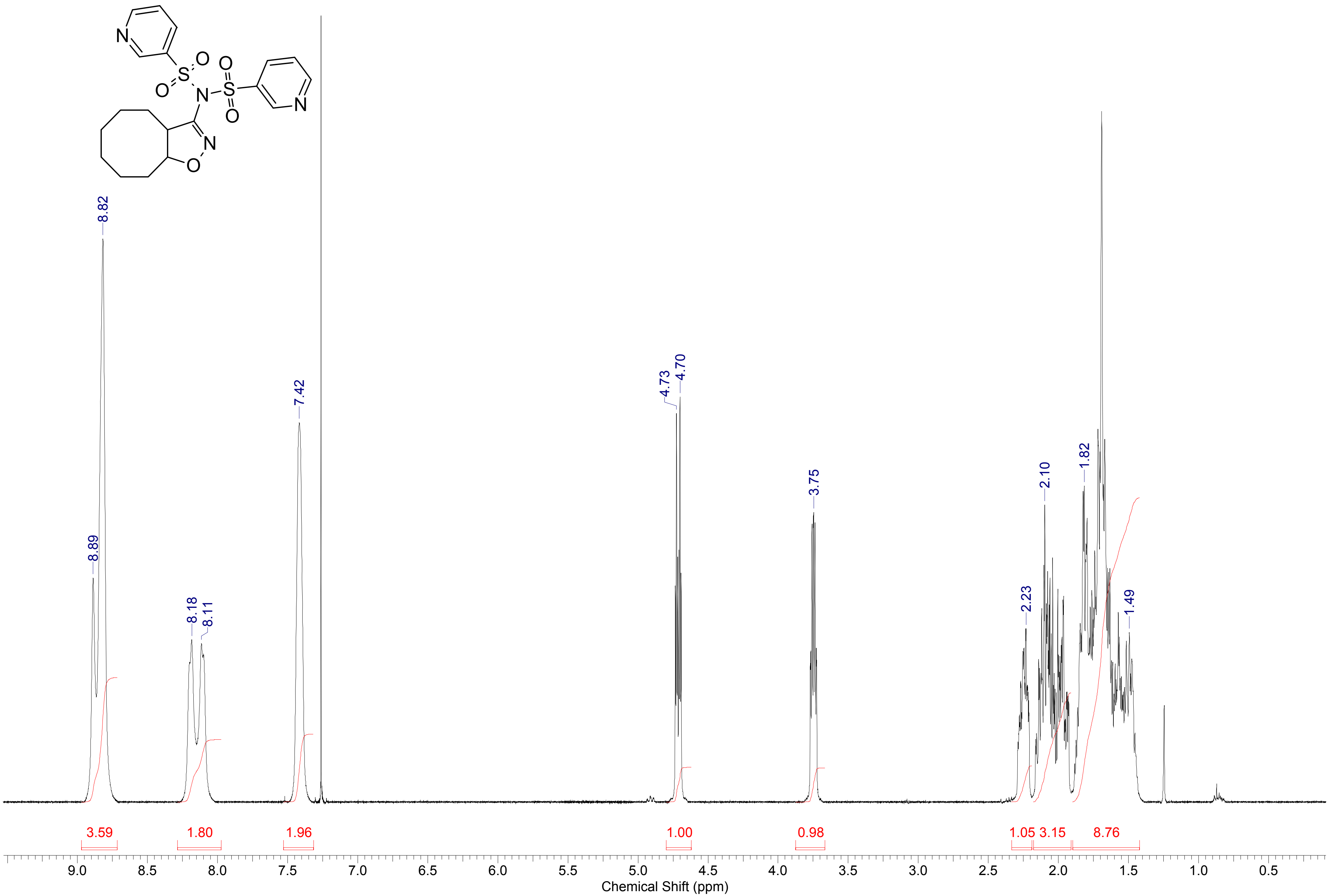

^{13}C NMR spectrum of compound **5l** ($CDCl_3$)

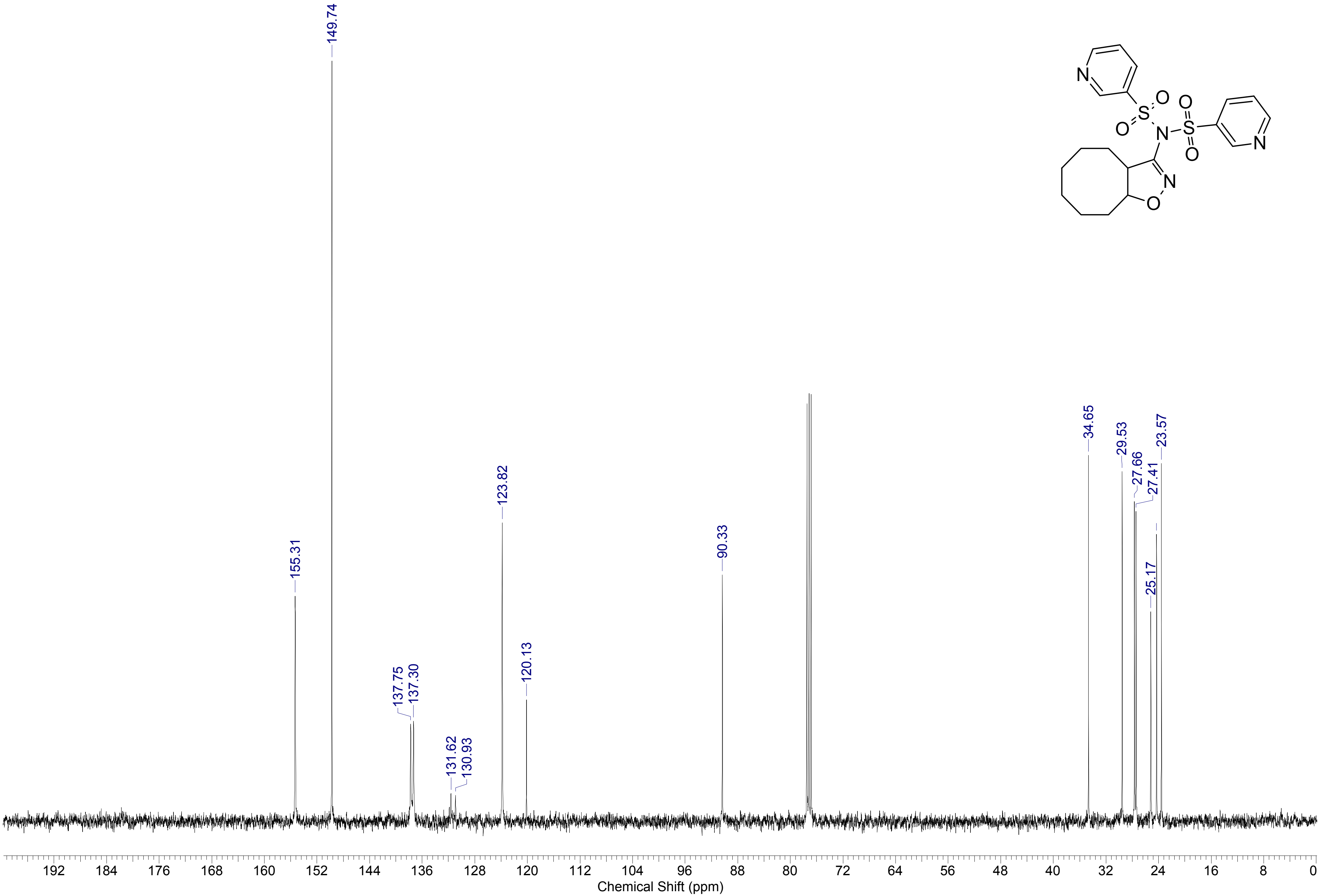

HSQC NMR spectrum of compound **5k** ($CDCl_3$)

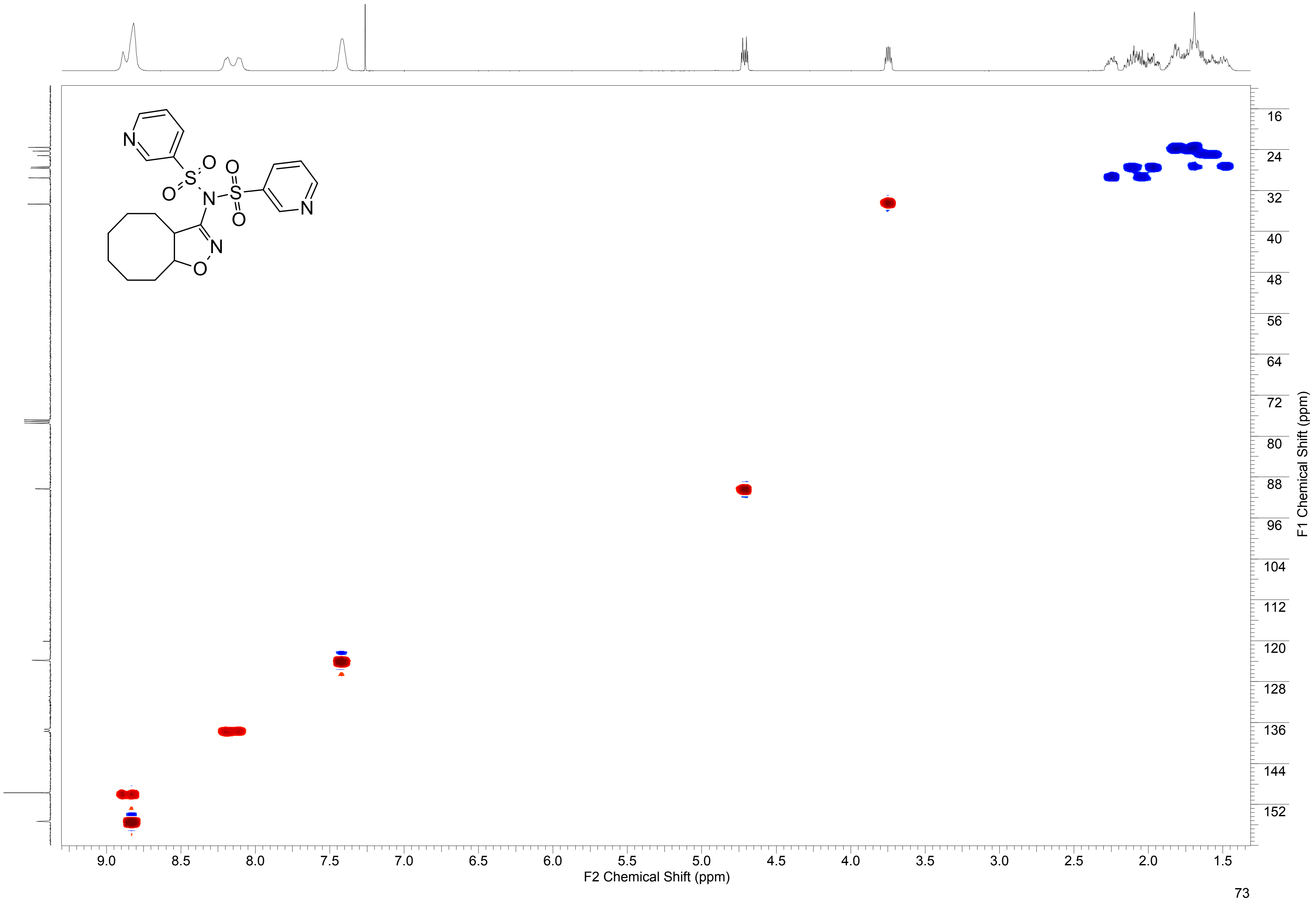

Supplement: Supplementary file 1 [file ijms-24-10758-s001.zip › ijms-2462697-supplementary.pdf]
